# Rh(iii)-catalyzed diastereoselective C–H bond addition/cyclization cascade of enone tethered aldehydes†
†Electronic supplementary information (ESI) available. CCDC 1413993 for **3b** and 1431932 for **10**. For ESI and crystallographic data in CIF or other electronic format see DOI: 10.1039/c5sc04138d


**DOI:** 10.1039/c5sc04138d

**Published:** 2015-12-01

**Authors:** Jeffrey A. Boerth, Jonathan A. Ellman

**Affiliations:** a Department of Chemistry , Yale University , Connecticut 06520 , USA . Email: jonathan.ellman@yale.edu ; Tel: +1-203-432-2647

## Abstract

The Rh(iii)-catalyzed cascade addition of a C–H bond across alkene and carbonyl π-bonds is reported. The reaction proceeds under mild reaction conditions with low catalyst loading. A range of directing groups were shown to be effective as was the functionalization of alkenyl in addition to aromatic C(sp^2^)–H bonds. When the enone and aldehyde electrophile were tethered together, cyclic β-hydroxy ketones with three contiguous stereocenters were obtained with high diastereoselectivity. The intermolecular three-component cascade reaction was demonstrated for both aldehyde and imine electrophiles. Moreover, the first X-ray structure of a cationic Cp*Rh(iii) enolate with interatomic distances consistent with an η^3^-bound enolate is reported.

## Abstract 2

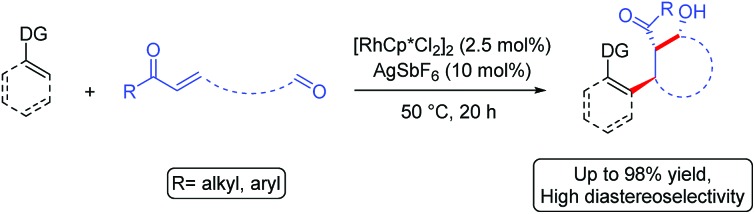
Rh(iii)-catalyzed cascade addition of C–H bonds across alkene and carbonyl π-bonds to form two new σ C–C bonds is accomplished.

## Introduction

**Fig. 1:**
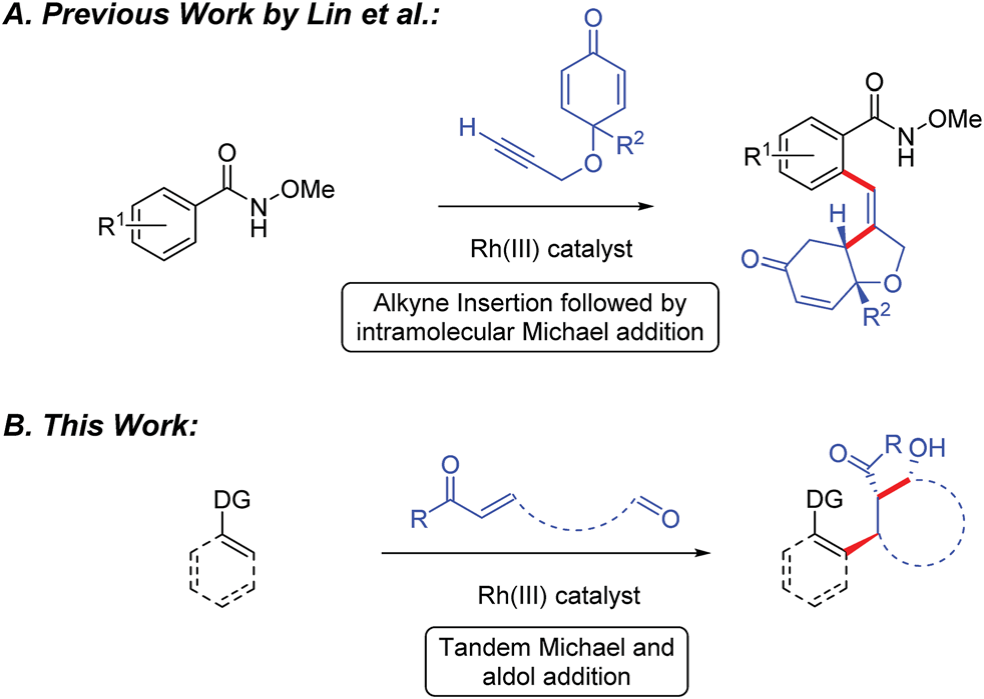
Rh(iii)-catalyzed cascade C–H bond addition across two different π-bonds.

Recently, the design and implementation of Rh(iii)-catalyzed C–H bond functionalization has led to a diverse array of structural motifs, including many that are present in drugs and natural products.^1^–^3^ In particular, Rh(iii)-catalyzed C–H bond additions to polarized π-bonds^3^ provides convergent entry to drug relevant amines,^4^ amides,^5^ alcohols,^6^ and oxygen or nitrogen heterocycles.^7^ While a variety of methods have been developed for direct C(sp^2^)–H bond addition into polarized π-bonds, cascade addition sequences would offer an attractive strategy for rapidly building complexity into organic structures. To date, Rh(iii)-catalyzed cascade C–H bond functionalization has primarily been reported for 5-membered ring synthesis by additions to an alkene or alkyne followed by cyclization upon the directing group for C–H bond activation.^8^ However, to the best of our knowledge, Lin and coworkers have reported the only example of Rh(iii)-catalyzed C–H bond cascade addition to a π-bond and an electrophile other than the directing group.^9^ In their study, Rh(iii)-catalyzed C–H bond addition to enones tethered to an alkyne proceeded to give substituted tetrahydrofurans (Fig. 1A). Herein, we demonstrate the Rh(iii)-catalyzed cascade addition of a C–H bond across alkene and carbonyl π-bonds. When the two electrophiles are tethered together, cyclic β-hydroxy ketone products incorporating three contiguous stereocenters are produced with high diastereoselectivity (Fig. 1B).^10^,^11^

## Results and discussion

For our initial exploration of this reaction we employed 2-phenylpyridine and the enone tethered aldehyde **2a** based on successful Rh(iii)-catalyzed hydroarylation of enones (Table 1).^12^ After considerable optimization, we found that β-hydroxy ketone **3a** could be obtained in high yield as a single diastereomer with only 2.5 mol% of Rh precatalyst and 10 mol% of AgSbF_6_ (entry 1). A 3 : 2 dioxane/H_2_O reaction solvent at 50 °C was found to be effective. Lower conversion was observed at 30 °C (entry 2). Solvents commonly used in Rh(iii)-catalyzed C–H functionalization such as dichloroethane (entry 3) and dioxane (entry 4) provided lower yields, and reducing the amount of H_2_O also was detrimental (entry 5). In contrast, acetic acid as the solvent proved to be optimal,7a,12a giving a near quantitative yield of the desired β-hydroxy ketone **3a** (entry 6). Additionally, **3a** was obtained in 57% yield when employing only the rhodium dimer [Cp*RhCl_2_]_2_ in the absence of a silver halide abstractor demonstrating that a pre-formed cationic Rh catalyst is not required for this transformation (entry 7). However, when rhodium was excluded, the desired product **3a** was not obtained (entries 8 and 9).

**Table 1:** Optimization conditions for the Rh(iii)-catalyzed cascade addition/cyclization reaction^*a*^ ^*b*^

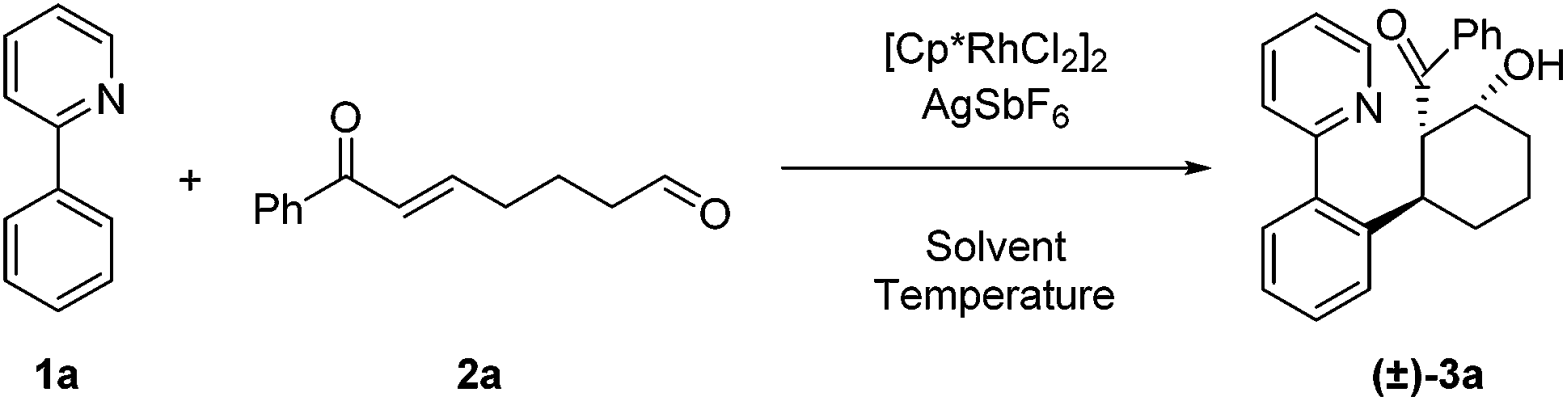				
Entry	Rh (mol%)/Ag (mol%)	Solvent	Temp (°C)	Yield **3a**^*b*^ (%)
1	(2.5)/(10)	3 : 2 Dioxane/H_2_O	50	92
2	(2.5)/(10)	3 : 2 Dioxane/H_2_O	30	67
3	(2.5)/(10)	DCE	50	58
4	(2.5)/(10)	Dioxane	50	35
5	(2.5)/(10)	95 : 5 Dioxane/H_2_O	50	61
6	(2.5)/(10)	Acetic acid	50	99
7	(2.5)/(0)	Acetic acid	50	57
8	(0)/(10)	Acetic acid	50	0
9	None	Acetic acid	50	0

^*a*^Conditions: **1a** (2.0 equiv.), **2a** (1.0 equiv.) using [Cp*RhCl_2_]_2_ and AgSbF_6_ for 20 h (0.2 M).

^*b*^Determined by NMR analysis relative to 1,3,5-trimethoxybenzene as an external standard.

After identifying optimal conditions for the formation of β-hydroxy ketone **3a**, we next explored the scope of the tethered electrophile substrate (Table 2). Pure **3a** was isolated in near quantitative yield from the parent substrate **2a**. Substrates with electron-donating and withdrawing substituents on the phenyl ring also afforded β-hydroxy ketones **3b**, **3c**, and **3d** in high yield. X-ray structural analysis of **3b** provided rigorous confirmation of product stereochemistry. An alkyl enone, which is a weaker acceptor than the corresponding aryl enone substrates, proved to be effective giving **3e** with only a modest reduction in yield. Introduction of oxygen and nitrogen heteroatoms into the tether were also acceptable substitutions, and provided the substituted tetrahydropyran **3f** and piperidine **3g**, respectively. Additionally, the use of an aromatic tether was well-tolerated and gave indane **3h** in good yield. However, for this substrate, 3 : 2 dioxane/H_2_O as solvent, a higher catalyst loading, and a lower reaction temperature were employed to minimize β-hydroxyl group elimination, which is particularly facile for β-hydroxy ketone **3h**.

**Table 2:** Scope for tethered electrophile partner^*a*^ ^*b*^

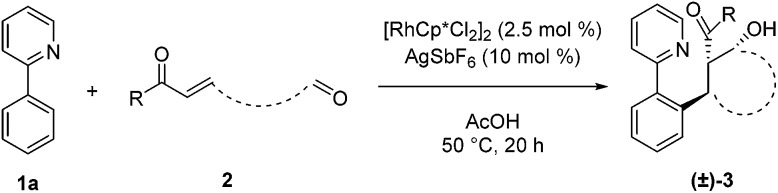
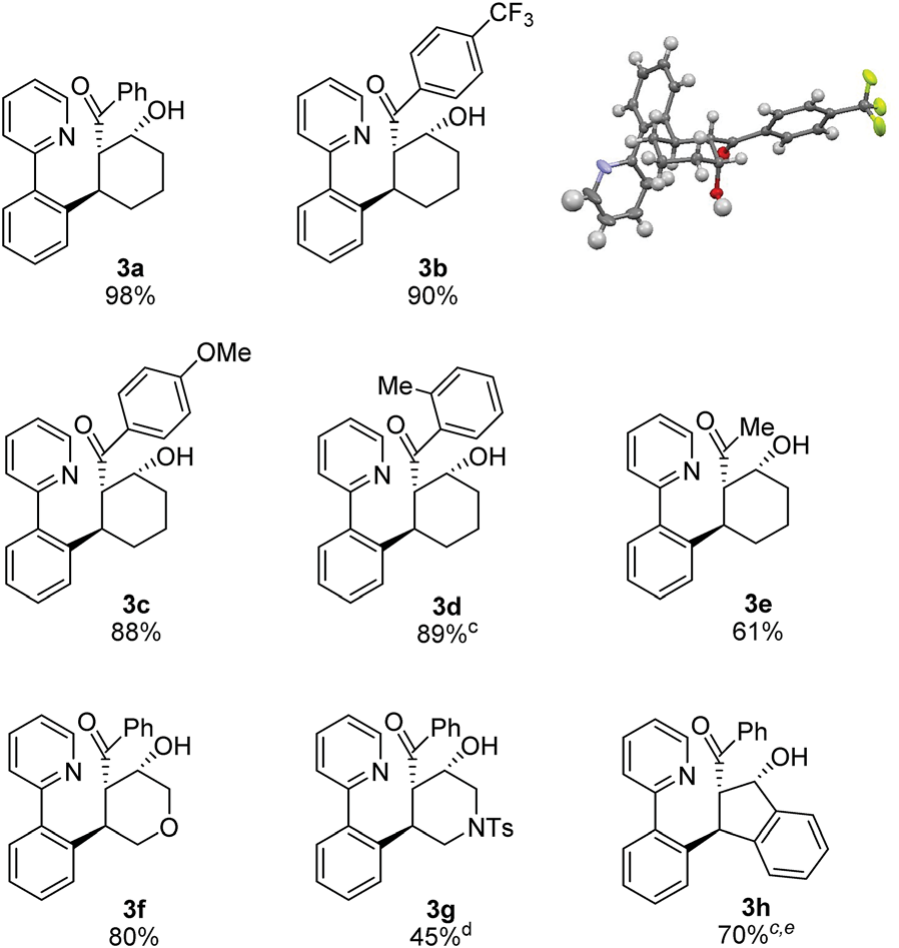

^*a*^Conditions: **1a** (2.0 equiv.), **2** (1.0 equiv.), at 0.2 M.

^*b*^Isolated yield after silica gel chromatography.

^*c*^Reaction conducted at 40 °C.

^*d*^Reaction conducted in 95 : 5 AcOH/H_2_O (0.2 M).

^*e*^Reaction conducted using 10 mol% [RhCp*Cl_2_]_2_ and 20 mol% AgSbF_6_ in 3 : 2 dioxane/H_2_O (0.2 M).

We next examined the scope for different C–H bond coupling partners (Table 3). Derivatives of 2-phenylpyridine with electron-donating and withdrawing substituents also provided high yields of the corresponding β-hydroxy ketones **3i**, **3j**, and **3k**. Notably, the applicability of alkenyl C(sp^2^–H) functionalization to this transformation was demonstrated with 2-cyclohexenylpyridine, which gave **3l** in good yield. Deviations from the pyridyl directing group are also noteworthy. The pyrazole and pyrimidine heterocycles were quite efficient in forming products **3m** and **3n**. *N*-Pyrimidylindole provided **3o**, highlighting C–H functionalization on a heteroaryl ring. In addition, the *N*-methoxybenzamide and *O*-methyl oxime directing groups provided access to the products **3p** and **3q** in moderate yields. For these substrates, higher yields were obtained with 3 : 2 dioxane/H_2_O rather than acetic acid as solvent.

**Table 3:** Scope for C–H bond partner^*a*^ ^*b*^

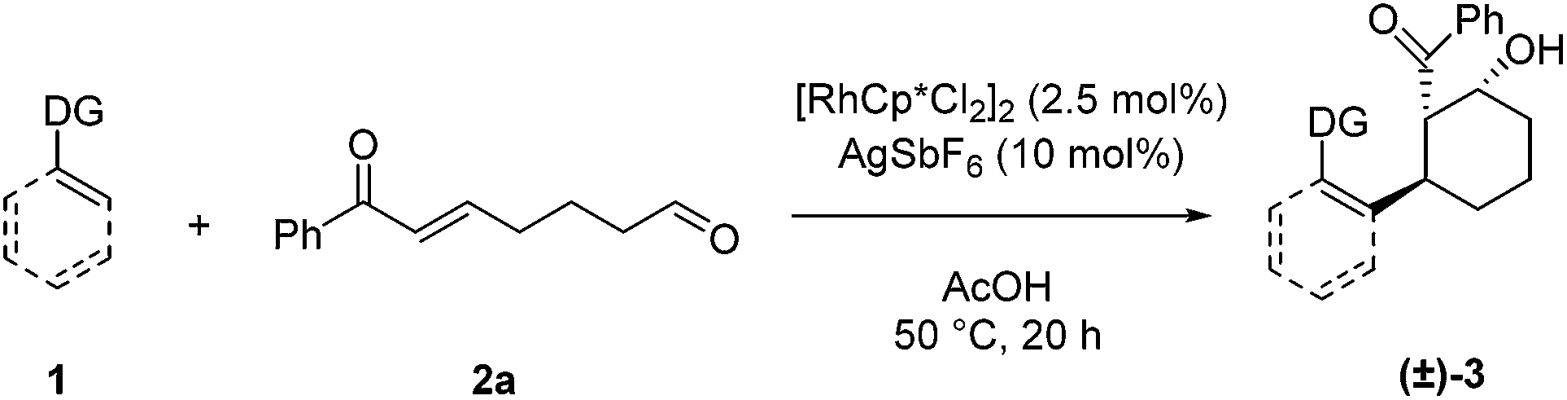
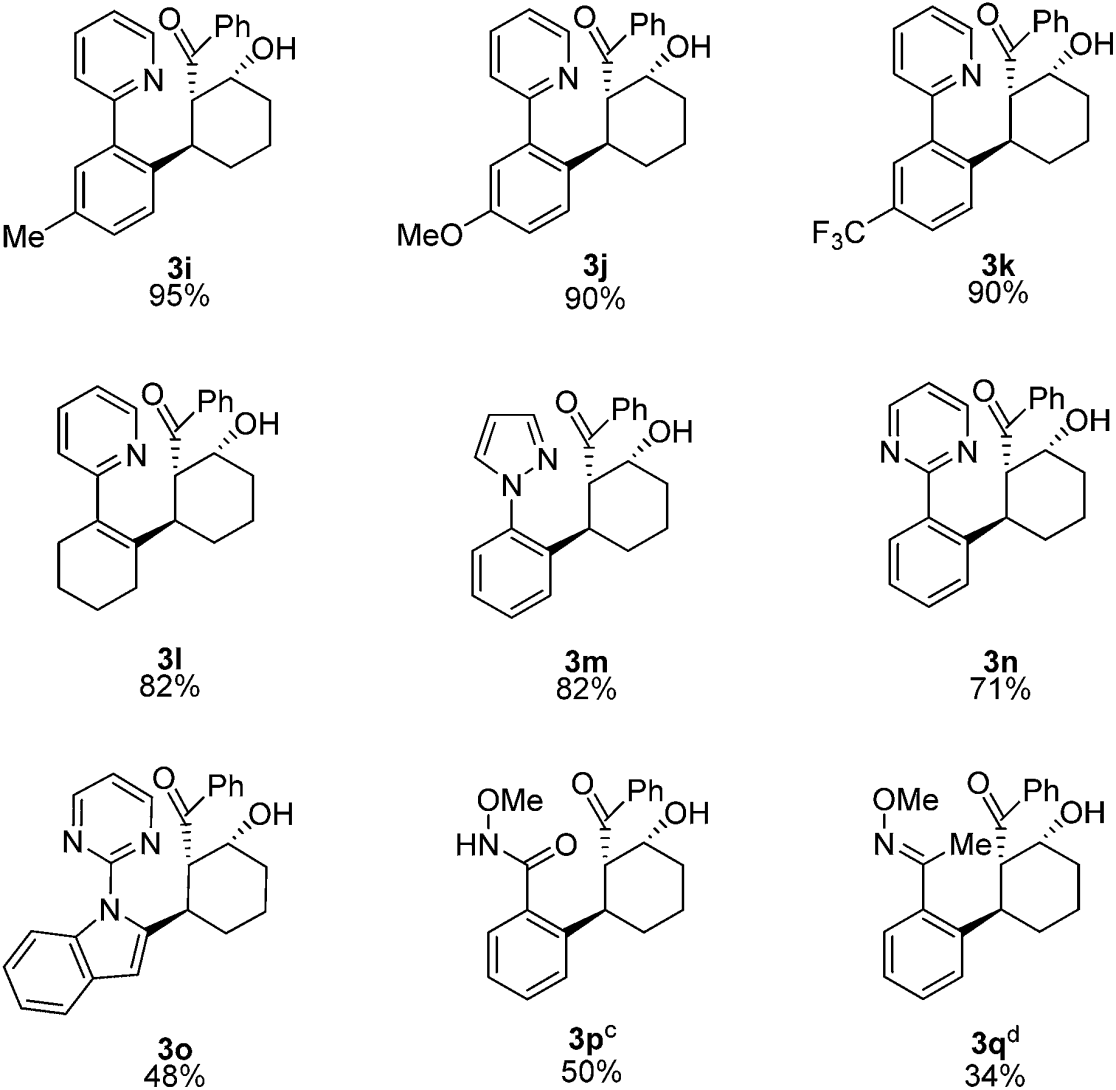

^*a*^Conditions: **1** (2.0 equiv.), **2a** (1.0 equiv.) at 0.2 M.

^*b*^Isolated yield after silica gel chromatography.

^*c*^Reaction conducted in 3 : 2 dioxane/H_2_O (0.5 M).

^*d*^Reaction conducted in 3 : 2 dioxane/H_2_O (0.2 M).

**Scheme 1:**
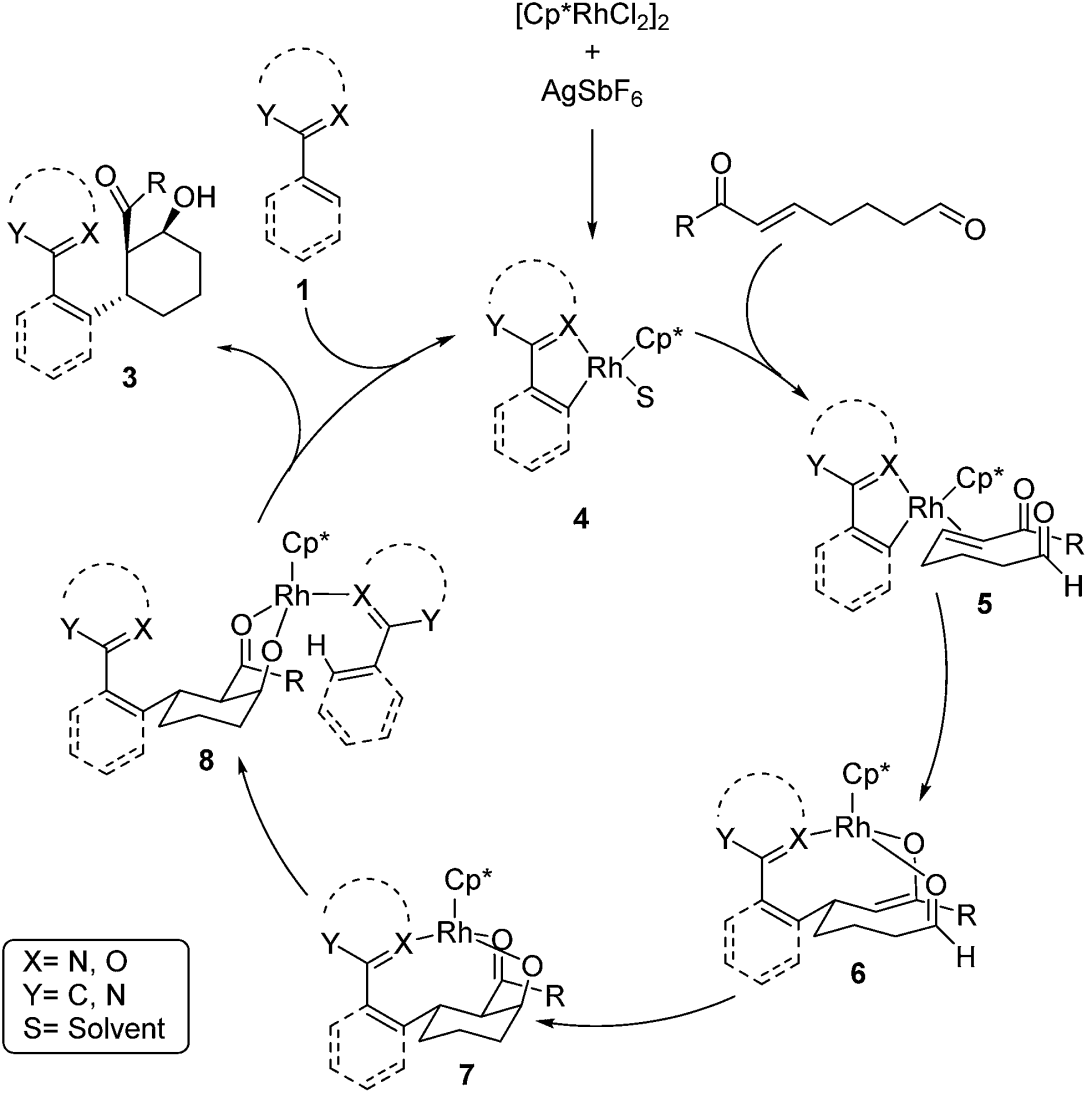
Proposed mechanism for transformation.

A mechanism for the Rh-catalyzed cascade C–H bond addition/aldol reaction is depicted in Scheme 1. The first step of this process proceeds *via* concerted metalation/deprotonation of **1** to generate rhodacycle **4**, which has previously been proposed as an intermediate in other Rh-catalyzed C–H functionalization reactions.^3^,^13^ Coordination of the enone π-bond provides **5**, which undergoes conjugate addition to give rhodium enolate **6**. The rhodium enolate **6** can then undergo an intramolecular aldol reaction with the tethered aldehyde to form rhodium alkoxide **7**.^14^ Coordination of another equivalent of **1** to the Rh–alkoxide complex then provides **8**, which undergoes concerted metalation/deprotonation to release the alcohol product **3** and regenerate the active rhodium species **4**.^15^

To ascertain whether or not cyclization upon the aldehyde carbonyl after enone addition proceeds *via* the proposed Rh-enolate intermediate **6** (Scheme 1), the independently prepared acyclic enone addition product **9a** was subjected to the reaction conditions with and without the cationic rhodium catalyst (Table 4). With dioxane/H_2_O as the solvent (entries 1 and 2), a low yield of β-hydroxy ketone **3a** was obtained even in the presence of the Rh precatalyst and AgSbF_6_ (entry 1). In addition, whether or not Rh was added, complete consumption of **9a** was observed, presumably as a result of selective formation of enolates or enols from the more acidic aldehyde functionality followed by unproductive side-reactions. These results are consistent with aldol cyclization proceeding *via* the Rh-enolate generated upon enone addition. When acetic acid was used as the solvent, a moderate yield of **3a** was obtained suggesting that acetic acid can mediate this cyclization step, although once again decomposition pathways competed with the desired cyclization pathway (entries 3 and 4).

**Table 4:** Control studies on reaction intermediate **9a**^*a*^

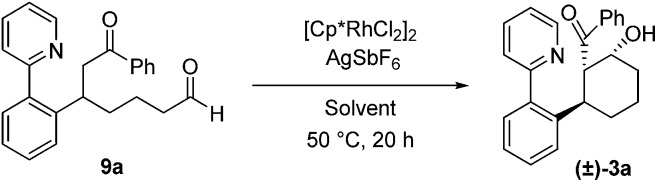				
Entry	Rh (mol%)/Ag (mol%)	Solvent	Yield **3a**^*a*^ (%)	Remaining **9a**^*a*^ (%)
1	(2.5)/(10)	3 : 2 Dioxane/H_2_O	11	1
2	None	3 : 2 Dioxane/H_2_O	12	1
3	(2.5)/(10)	Acetic acid	44	1
4	None	Acetic acid	45	<1

^*a*^Yield determined by NMR analysis relative to 1,3,5-trimethoxybenzene as an external standard.

**Fig. 2:**
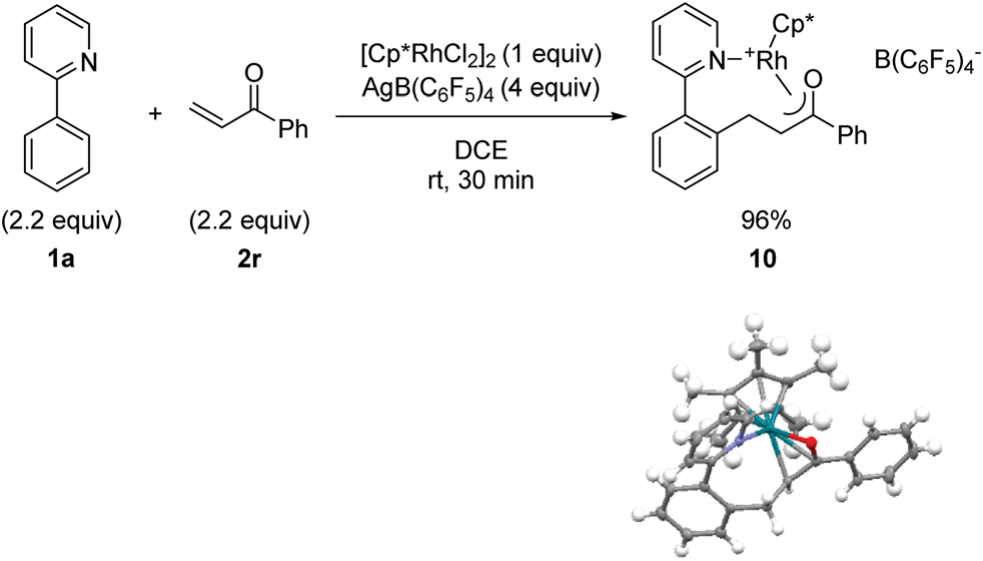
Preparation, isolation and X-ray structural characterization of a cationic Rh(iii)-enolate. The B(C_6_F_5_)_4_ ion has been omitted from the X-ray structure for clarity.

C–H bond addition to generate a rhodium-enolate intermediate was rigorously established using the simplified enone substrate phenyl vinyl ketone (Fig. 2). To facilitate crystallization of the Rh-enolate, tetrakis(pentafluorophenyl)borate was employed as the counterion with DCE as the solvent, which for the cascade reaction is only slightly less effective than acetic acid or dioxane/water as solvent, *vide infra* (see **3r** in Table 5). After only 30 min at rt, rhodacycle **10** was isolated in very high yield. The X-ray structure of **10** shows a coordinatively saturated Rh(iii) complex with interatomic distances consistent with an η^3^-bound enolate. Although rhodium enolates have been proposed as intermediates for a number of transformations,^16^ very few X-ray structures have been reported.^17^ To our knowledge, the only published example of a Cp*Rh(iii) enolate is for a neutral complex with an η^1^ C-bound enolate.17*a*

**Table 5:** Three-component coupling reaction^*a*^ ^*b*^

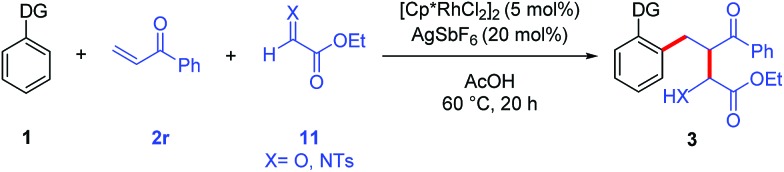
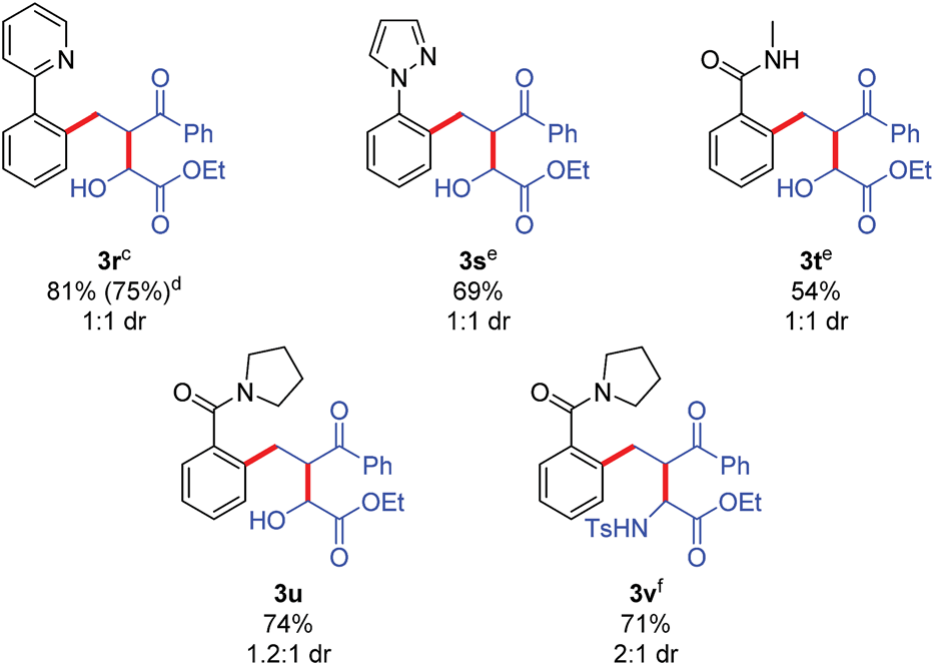

^*a*^Conditions: **1** (1.0 equiv.), **2r** (1.1 equiv.), and **11** (2.0 equiv.) at 2.0 M.

^*b*^Isolated yield after silica gel chromatography.

^*c*^Reaction conducted at 40 °C.

^*d*^Reaction conducted in DCE.

^*e*^Reaction conducted at 50 °C.

^*f*^Reaction conducted in DCE using crushed 3 Å molecular sieves.

The potential for performing an intermolecular three-component C–H activation/addition/aldol reaction cascade was also evaluated (Table 5). Several different directing groups provided three-component cascade addition products using enone **2r** along with either an activated aldehyde or imine. Coupling 2-phenylpyridine and *N*-phenyl pyrazole with enone **2r** and ethyl glyoxylate gave products **3r** and **3s** in good yields, respectively. Moreover, the synthetically more versatile secondary and tertiary amide directing groups provided products **3t** and **3u** in moderate to good yields. In addition, three-component coupling with the *N*-tosyl imine derived from ethyl glyoxylate efficiently provided amine **3v**, though the use of DCE as solvent was necessary to minimize the competitive imine hydrolysis that occurred in acetic acid.

The complete selectivity for initial C–H bond addition to the enone rather than the aldehyde is an interesting feature of this reaction. This outcome might result from kinetic control with C–H bond addition to the enone occurring much faster than to the aldehyde. Alternatively, thermodynamic control might be operative because C(sp^2^)–H bond addition to aldehydes is known to be reversible,^18^ although C–H bond addition products are favored for destabilized aldehydes such as ethyl glyoxylate.6*f*

## Conclusions

In summary, a Rh(iii)-catalyzed C–H bond addition/aldol cyclization cascade has been developed and represents the first C–H bond addition across an alkene π-bond and a carbonyl. This robust transformation can be carried out under mild conditions and for tethered substrates generates three contiguous stereocenters with high diastereoselectivity. An intermolecular three-component C–H bond addition/aldol reaction cascade has also been demonstrated. Preliminary mechanistic studies also provide the first X-ray structural characterization of a cationic Cp*Rh(iii) enolate. In continuing efforts we are broadening the scope of this class of cascade reactions to include different tethered electrophilic species. We are also actively investigating stereoselective intermolecular three-component reactions with different carbon–carbon π-bonds and carbonyl/imine electrophiles.

## Supplementary Material

Supplementary information

Crystal structure data
