# Supplementary material for: Rh(iii)-catalyzed diastereoselective C–H bond addition/cyclization cascade of enone tethered aldehydes
Source: Chem Sci. 2015 Dec 1;7(2):1474–9. doi: 10.1039/c5sc04138d (PMC4762265; doi:10.1039/c5sc04138d)

Electronic Supplementary Material (ESI) for Chemical Science.
This journal is © The Royal Society of Chemistry 2015

# Rh(III)-Catalyzed Diastereoselective C–H Bond Addition/Cyclization Cascade of Enone Tethered Aldehydes

Jeffrey A. Boerth and Jonathan A. Ellman*

## Supporting Information

I. General Information S-2

II. Preparation of Substrates S-2

III. Procedures for Rh(III)-Catalyzed Tandem Addition/Cyclization S-4

IV. Control Experiments with Intermediate **9a** S-20

V. Procedure for Rhodacycle Synthesis S-24

VI. X-Ray Crystallographic Data S-25

VII. References S-38

VIII. NMR Data S-39

## I. General Information:

Unless otherwise indicated, all Rh(III)-catalyzed reactions were set up in a $N_2$ filled glovebox, using glassware that was oven-dried (150 °C) and evacuated while hot prior to use. Unless otherwise indicated, all reactions for substrate preparation were carried out on the benchtop under a $N_2$ atmosphere. Solvents were purified by elution through a column of activated alumina under $N_2$ before use. Methanol was distilled from $CaH_2$ under nitrogen prior to use. Glacial acetic acid was sparged with $N_2$ before use. Unless otherwise noted, all reagents were purchased from commercial sources and used without further purification. Products and starting materials were visualized on TLC using UV-light or by staining with $KMnO_4$. Flash-column chromatography was preformed on SiliaFlash® P60 (230-400 mesh) silica gel, and preparative thin-layer chromatrography plates from Analtech (1 mm $SiO_2$, 20 x 20 cm) were used. NMR chemical shifts are reported in ppm relative to $CDCl_3$ (7.26 ppm for $^1$H and 77.16 ppm for $^{13}$C) or $CD_2Cl_2$ (5.32 ppm for $^1$H and 53.84 ppm for $^{13}$C). Trifluoroacetic acid (set to -76.55 ppm in $CDCl_3$) was used for standardizing $^{19}$F NMR chemical shifts. For IR spectra, only partial data are provided. Melting points are reported uncorrected. High-resolution mass spectra (HRMS) were obtained using electrospray ionization (ESI) on a time of flight (TOF) mass spectrometer.

## II. Preparation of Substrates:

### Catalysts:

$[Cp^*RhCl_2]_2$[S1] and $AgB(C_6F_5)_4(Et_2O)_2$[S2] were synthesized according to a published literature procedure.

### Substrates:

1-tosyl-2,5-dihydro-1*H*-pyrrole,[S3] (*E*)-7-oxo-7-phenylhept-5-enal,[S4] (*E*)-7-oxo-7-(4-(trifluoromethyl)phenyl)hept-5-enal,[S5] (*E*)-7-(methoxyphenyl)-7-oxohept-5-enal,[S6] (*E*)-7-oxo-7-(*o*-tolyl)hept-5-enal,[S7] (*E*)-7-oxooct-5-enal,[S8] (*E*)-2-(3-oxo-3-phenylprop-1-en-1-yl)benzaldehyde,[S9] 2-(*m*-tolyl)pyridine,[S10] 2-(3-methoxyphenyl)pyridine,[S10] 2-(3-(trifluoromethyl)phenyl)pyridine,[S10] 2-(cyclohex-1-en-1-yl)pyridine,[S11] 1-(pyrimidin-2-yl)-1*H*-indole,[S12] *N*-methoxybenzamide,[S13] (*E*)-1-phenylethan-1-one *O*-methyl oxime,[S14] phenyl vinyl

ketone,[S15] phenyl(pyrrolidin-1-yl)methanone,[S16] and (*E*)-ethyl-2-(tosylimino)acetate[S17] were synthesized according to literature procedure. All other C–H activation substrates were purchased from commercial sources and used without further purification.

**Procedure for Synthesis of (*E*)-2-((4-Oxo-4-phenylbut-2-en-1-yl)oxy)acetaldehyde:**

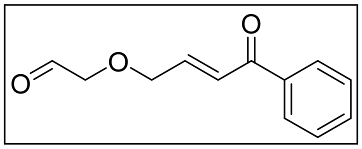

In a 500-mL round bottom flask equipped with a magnetic stir bar, 2,5-dihydrofuran (7.01 g, 0.100 mol, 10.0 equiv) was dissolved in 105 mL of $CH_2Cl_2$. The reaction mixture was cooled in a dry ice/acetone bath and ozone was bubbled in until a faint blue color persisted for 5 min. At this point the reaction mixture was purged with $N_2$ gas and kept under nitrogen for the duration of the reaction. Once the blue color had dissipated, triphenylphosphine (26.3 g, 0.100 mol, 10.0 equiv) was added, and the reaction mixture was placed in an ice/acetone bath and stirred for 1 h. At this point, (benzoylmethylene)triphenylphosphorane (3.81 g, 10.0 mmol, 1.00 equiv) was added and the reaction mixture was stirred for an additional 4 h. The reaction mixture was then warmed to ambient temperature and concentrated. The crude material was triturated with diethyl ether until no product remained in the residual solid as indicated by TLC. The combined ether washes were concentrated, dissolved in a minimal amount of $CH_2Cl_2$, and purified by silica gel chromatography using 1:1 ethyl acetate/hexane to obtain the aldehyde (**2f**) as a colorless oil (0.762 g, 37%). IR (film): 2856, 1734, 1671, 1624, 1282, 1017, 960.5, 765.0 $cm^{-1}$; $^1H$ NMR (400 MHz, $CDCl_3$) δ 9.78 (s, 1H), 7.98-7.96 (m, 2H), 7.60-7.56 (m, 1H), 7.49 (t, *J* = 7.5 Hz, 2H), 7.22 (dt, *J* = 15.5, 1.9 Hz, 1H), 7.04 (dt, 15.5, 4.1 Hz, 1H), 4.39 (dd, *J* = 4.1, 1.9 Hz, 2H), 4.21 (s, 2H). $^{13}C\{^1H\}$ NMR ($CDCl_3$, 126 MHz): δ 199.62, 190.15, 142.71, 137.76, 133.14, 128.80, 125.92, 76.29, 70.92; HRMS (ESI/[M+H]+) calcd. for $C_{12}H_{12}O_3$: 205.0859 Found 205.0857.

**Procedure for Synthesis of (*E*)-4-methyl-*N*-(4-oxo-4-phenylbut-2-en-1-yl)-*N*-(2-oxoethyl)benzenesulfonamide:**

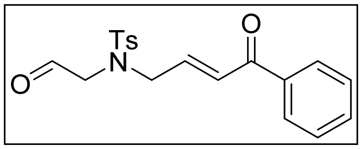


In a 500-mL round bottom flask equipped with a magnetic stir bar, 1-tosyl-2,5-dihydro-1*H*-pyrrole[S3] (3.90 g, 17.5 mol, 5.00 equiv) was dissolved in 60 mL of $CH_2Cl_2$. The reaction mixture was cooled in a dry ice/acetone bath and ozone was bubbled in until a faint blue color persisted for 5 min. At this point the reaction mixture was purged with $N_2$ gas and kept under nitrogen for the duration of the reaction. Once the blue color had dissipated, triphenylphosphine (26.3 g, 0.100 mol, 10.0 equiv) was added, and the reaction mixture was placed in an ice/acetone bath and stirred for 1 h. At this point, (benzoylmethylene)triphenylphosphorane (4.59 g, 17.5 mmol, 5.00 equiv) was added and the reaction mixture was stirred for an additional 4 h. The reaction mixture was then warmed to ambient temperature and concentrated. Afterwards the crude reaction product was dissolved in a minimal amount of $CH_2Cl_2$, and purified by a silica plug using 1:1 ethyl acetate/hexane to separate the desired product from triphenylphosphine oxide. The desired fractions were then concentrated and purified by four preparative TLC plates using a 40/60 mixture of ethyl acetate/hexane obtain the product as a pale yellow waxy solid (**2g**), likely the hydrate/oligomer (0.601 g, 48%). $^1$H NMR (500 MHz, $CDCl_3$) crude shown in NMR Data Section; HRMS (ESI/[M+H]+) calcd. for $C_{19}H_{19}NO_4S$: 358.1108 Found 358.1115.

## III. Procedures for Rh(III)-Catalyzed Tandem Addition/Cyclization:

### General Procedure:

In a $N_2$-filled glove box, a 2-5 mL microwave vial was charged with $AgSbF_6$ (6.9 mg, 0.020 mmol, 0.10 equiv), $[Cp^*RhCl_2]_2$[S1] (3.1 mg, 0.0050 mmol, 0.025 equiv), the corresponding aldehyde (0.20 mmol, 1.0 equiv), and the C–H activation substrate (0.40 mmol, 2.0 equiv). Acetic acid (1.0 mL) was then added and the vial was equipped with a stir bar. The reaction vial was sealed and then outside the glove box, the vial was heated at 50 °C in a preset oil bath for 20 h with stirring. The reaction mixture was then cooled to room temperature, and filtered over a plug of celite (1 cm

celite in a glass pipette) with $CH_2Cl_2$. The filtrate then was concentrated and purified by flash column chromatography using an ethyl acetate/hexanes solvent system to afford the desired product.

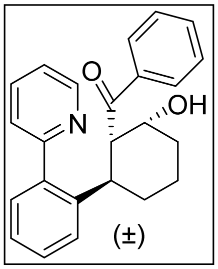

**(±)-((1*S*,2*R*,6*R*)-2-Hydroxy-6-(2-(pyridin-2-yl)phenyl)cyclohexyl)(phenyl)methanone (3a):** The general procedure using (*E*)-7-oxo-7-phenylhept-5-enal[S4] (40.5 mg, 0.200 mmol, 1.0 equiv), and 2-phenylpyridine (62.1 mg, 0.400 mmol, 2.0 equiv) was followed. Chromatography eluting with a 1:2 solution of ethyl acetate/hexanes provided the product **3a** (70.1 mg, 98% yield) as a white solid (mp: 138-140 °C). IR (film): 3226, 2926, 2857, 1682, 1589, 996, 751, 702 $cm^{-1}$; $^1$H NMR (500 MHz, $CD_2Cl_2$) δ 8.70 (d, *J* = 4.3 Hz, 1H), 7.83 (dt, *J* = 7.7, 1.6 Hz, 1H), 7.78 (d, *J* = 7.5 Hz, 2H), 7.53-7.50 (m, 2H), 7.39 (t, *J* = 7.7 Hz, 2H), 7.34-7.31 (m, 2H), 7.20-7.15 (m, 2H), 7.10 (t, *J* = 7.4 Hz, 1H), 4.17 (s, 1H), 4.01 (d, *J* = 11.5 Hz, 1H), 3.74 (dt, *J* = 11.8, 3.5 Hz, 1H), 2.57 (s, 1H), 2.04 (apparent d, *J* = 11.0 Hz, 1H), 1.92 (apparent d, *J* = 13.1 Hz, 1H), 1.80-1.71 (m, 1H), 1.63 (apparent d, *J* = 13.6 Hz, 1H), 1.59-1.52 (m, 2H). $^{13}$C{$^1$H} NMR ($CD_2Cl_2$, 126 MHz): δ 204.30, 160.54, 149.48, 142.71, 141.65, 137.58, 136.37, 133.54, 130.49, 128.95, 128.54, 128.52, 126.67, 126.23, 124.81, 122.12, 67.51, 54.08, 35.97, 35.07, 32.55, 20.24; HRMS (ESI/[M+H]+) calcd. for $C_{24}H_{23}NO_2$: 358.1802 Found 358.1792.

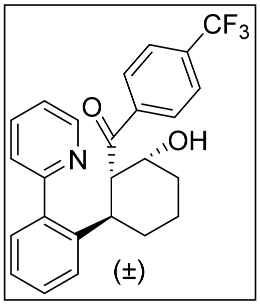

**(±)-((1S,2R,6R)-2-Hydroxy-6-(2-(pyridin-2-yl)phenyl)cyclohexyl)(4-(trifluoromethyl)phenyl)methanone (3b):** The general procedure using (*E*)-7-oxo-7-(4-(trifluoromethyl)phenyl)hept-5-enal[S5] (54.1 mg, 0.200 mmol, 1.00 equiv), and 2-phenylpyridine (62.1 mg, 0.400 mmol, 2.00 equiv) was followed. Chromatography eluting with a 1:1 solution of ethyl acetate/hexanes provided the product **3b** (76.6 mg, 90% yield) as a white solid (148-150 °C). IR (film): 3223, 2930, 1691, 1325, 1159, 1131, 1065, 753 $cm^{-1}$; $^1$H NMR (500 MHz, $CDCl_3$) δ 8.72 (d, *J* = 4.8 Hz, 1H), 7.84-7.79 (m, 3H), 7.61 (d, *J* = 8.0 Hz, 2H), 7.46 (d, *J* = 7.8 Hz, 1H), 7.30 (t, *J* = 6.8 Hz, 2H), 7.20 (d, *J* = 7.5 Hz, 1H), 7.14 (t, *J* = 7.4 Hz, 1H), 7.09 (t, *J* = 7.3 Hz, 1H), 4.20 (s, 1H), 3.94 (d, *J* = 11.8 Hz, 1H), 3.77 (dt, *J* = 11.9, 3.3 Hz, 1H), 2.85 (s, 1H), 2.09-2.05 (m, 1H), 1.99 (apparent d, *J* = 12.3 Hz, 1H), 1.86-1.77 (m, 1H), 1.60-1.55 (m, 3H). $^{13}$C{$^1$H} NMR ($CDCl_3$), 126 MHz): δ 204.12, 159.84, 149.28, 141.40, 141.06, 140.09, 136.28, 134.35 (q, *J* = 32.7 Hz), 130.33, 128.52, 128.36, 126.39, 125.60 (q, *J* = 3.6 Hz), 124.47, 123.64

(q, J = 272.8 Hz), 121.97, 66.95, 54.41, 35.88, 34.43, 32.07, 19.75; $^{19}$F NMR ($CDCl_3$, 470 MHz): δ -64.37 (s, 3F); HRMS (ESI/[M+H]+) calcd. for $C_{25}H_{22}F_3NO_2$: 426.1675 Found 426.1667.

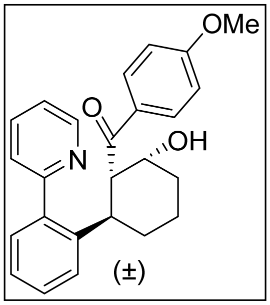


**(±)-((1S,2R,6R)-2-Hydroxy-6-(2-(pyridin-2-yl)phenyl)cyclohexyl)(4-methoxyphenyl)methanone (3c):** The general procedure using (*E*)-7-(methoxyphenyl)-7-oxohept-5-enal[S6] (46.5 mg, 0.200 mmol, 1.00 equiv), and 2-phenylpyridine (62.1 mg, 0.400 mmol, 2.00 equiv) was followed. Chromatography eluting with a 40/60 solution of ethyl acetate/hexanes provided the product **3c** (68.5 mg, 88% yield) as a white solid (mp: 140-142 °C). IR (film): 3458, 2932, 2858, 1673, 1598, 1253, 1170, 750 $cm^{-1}$; $^1$H NMR (400 MHz, $CDCl_3$) δ 8.71 (d, *J* = 4.1 Hz, 1H), 7.82-7.78 (m, 3H), 7.52 (d, *J* = 7.8 Hz, 1H), 7.34-7.32 (m, 1H), 7.30-7.28 (m, 1H), 7.21-7.14 (m, 2H), 7.09 (t, *J* = 7.3 Hz, 1H), 6.84 (d, *J* = 8.6 Hz, 2H), 4.14 (s, 1H), 3.95 (d, *J* = 11.5 Hz, 1H), 3.83 (s, 3H), 3.76 (dt, *J* = 11.9, 3.5 Hz, 1H), 3.14 (s, 1H), 2.03-1.95 (m, 2H), 1.80 (apparent q, *J* = 14.3 Hz, 1H), 1.58-1.50 (m, 3H). $^{13}C\{^1H\}$ NMR ($CDCl_3$), 101 MHz): δ 203.09, 163.86, 149.31, 141.71, 141.09, 136.15, 130.81, 130.58, 130.20, 130.00, 128.24, 126.15, 124.59, 121.86, 113.81, 113.63, 67.11, 55.61, 52.74, 35.71, 34.76, 31.95, 19.90; HRMS (ESI/[M+H]+) calcd. for $C_{25}H_{25}NO_3$: 388.1907 Found 388.1897.

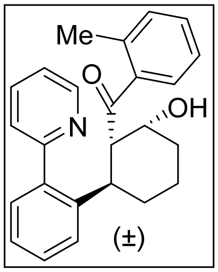


**(±)-((1S,2R,6R)-2-Hydroxy-6-(2-(pyridin-2-yl)phenyl)cyclohexyl)(*o*-tolyl)methanone (3d):** A modification to the general procedure was used. (*E*)-7-oxo-7-(*o*-tolyl)hept-5-enal[S7] (43.3 mg, 0.200 mmol, 1.00 equiv), and 2-phenylpyridine (62.1 mg, 0.400 mmol, 2.00 equiv) was added and stirred at 40 °C instead of 50 °C for 20 h. Chromatography eluting with a 40/60 solution of ethyl acetate/hexanes provided the product **3d** (66.2 mg, 89% yield) as a white solid (mp: 58-60 °C). IR (film): 3497, 2928, 2858, 1690, 1426, 994, 749, 730 $cm^{-1}$; $^1$H NMR (500 MHz, $CDCl_3$) δ 8.71 (d, *J* = 4.7 Hz, 1H), 7.81 (t, *J* = 7.6 Hz, 1H), 7.53 (d, *J* = 7.5 Hz, 1H), 7.43 (d, *J* = 7.7 Hz, 1H), 7.30-7.28 (m, 3H), 7.20-7.13 (m, 3H), 7.08 (t, *J* = 8.7 Hz, 2H), 4.31 (s, 1H), 3.81 (d, *J* = 11.7 Hz, 1H), 3.69 (dt, *J* = 11.8, 3.2 Hz, 1H), 3.15 (s, 1H), 2.06 (apparent d, *J* = 12.7 Hz, 1H), 1.99 (s, 3H), 1.99-1.96 (m, 1H), 1.83-1.75 (m, 1H), 1.56-1.46 (m, 3H). $^{13}C\{^1H\}$ NMR ($CDCl_3$), 126 MHz): δ 209.92, 159.90, 149.37, 141.28, 141.17, 139.63, 137.85, 136.25, 131.63, 131.16, 130.22, 128.14, 127.49, 126.72, 126.26, 125.49, 124.48, 121.92, 67.36, 56.84, 36.45, 35.17, 31.98, 20.00, 19.89; HRMS (ESI/[M+H]+) calcd. for $C_{25}H_{25}NO_2$: 372.1958 Found 372.1968.

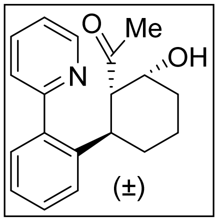


**(±)-1-((1S,2R,6R)-2-Hydroxy-6-(2-(pyridin-2-yl)phenyl)cyclohexyl)ethan-1-one (3e):** The general procedure using (*E*)-7-oxooct-5-enal[S8] (28.0 mg, 0.200 mmol, 1.00 equiv), and 2-phenylpyridine (62.1 mg, 0.400 mmol, 2.00 equiv) was followed. Chromatography eluting with a 70/30 solution of ethyl acetate/hexanes provided the product **3e** (36.1 mg, 61% yield) as an off-white solid (mp: 46-47 °C). IR (film): 3452, 2929, 1692, 1585, 1425, 1083, 795, 749 cm$^{-1}$; $^{1}$H NMR (500 MHz, $CDCl_3$) δ 8.67 (d, *J* = 4.8 Hz, 1H), 7.75 (dt, *J* = 7.7, 1.5 Hz, 1H), 7.43-7.37 (m, 3H), 7.32 (d, *J* = 7.2 Hz, 1H), 7.28-7.24 (m, 2H), 4.16 (s, 1H), 3.50 (dt, *J* = 11.9, 3.3 Hz, 1H), 3.16 (s, 1H), 2.99 (d, *J* = 11.6 Hz, 1H), 1.97 (apparent d, *J* = 12.0 Hz, 1H), 1.89 (apparent d, *J* = 14.0 Hz, 1H), 1.80 (s, 3H), 1.77-1.69 (m, 1H), 1.52-1.41 (m, 3H). $^{13}C\{^{1}H\}$ NMR ($CDCl_3$, 126 MHz): δ 214.74, 159.61, 149.29, 141.86, 141.10, 136.23, 130.45, 128.61, 126.50, 124.34, 121.96, 66.62, 59.08, 35.63, 34.63, 32.15, 31.93, 19.73; HRMS (ESI/[M+H]+) calcd. for $C_{19}H_{21}NO_2$: 296.1645 Found 296.1626.

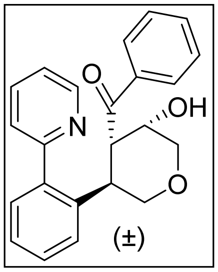


**(±)-((3S,4S,5R)-3-Hydroxy-5-(2-(pyridin-2-yl)phenyl)tetrahydro-2*H*-pyran-4-yl)(phenyl)methanone (3f):** The general procedure using (*E*)-2-((4-oxo-4-phenylbut-2-en-1-yl)oxy)acetaldehyde (40.8 mg, 0.200 mmol, 1.00 equiv), and 2-phenylpyridine (62.1 mg, 0.400 mmol, 2.00 equiv) was followed. Chromatography eluting with a 70/30 solution of ethyl acetate/hexanes provided the product **3f** (57.7 mg, 80% yield) as a white solid (mp: 179-181 °C). IR (film): 3365, 2962, 1674, 1587, 1092, 1023, 758, 697 cm$^{-1}$; $^{1}$H NMR (600 MHz, $CDCl_3$) δ 8.71 (d, *J* = 4.5 Hz, 1H), 7.87-7.84 (m, 3H), 7.69 (d, *J* = 7.7 Hz, 1H), 7.54 (t, *J* = 7.4 Hz, 1H), 7.42 (t, *J* = 7.7 Hz, 2H), 7.34-7.31 (m, 2H), 7.28-7.27 (m, 1H), 7.25-7.24 (m, 1H), 7.20 (t, *J* = 7.4 Hz, 1H), 4.21 (dd, *J* = 11.7, 2.9 Hz, 2H), 4.09 (s, 1H), 4.03-3.99 (m, 2H), 3.71 (d, *J* = 12.0 Hz, 1H), 3.45 (t, *J* = 11.4 Hz, 1H), 2.71 (s, 1H). $^{13}C\{^{1}H\}$ NMR ($CDCl_3$, 151 MHz): δ 200.37, 159.56, 149.23, 142.02, 136.83, 136.77, 136.48, 133.50, 130.63, 128.87, 128.57, 128.33, 127.03, 126.25, 124.65, 122.31, 72.88, 72.04, 66.58, 51.36, 34.89; HRMS (ESI/[M+H]+) calcd. for $C_{23}H_{21}NO_3$: 360.1594 Found 360.1584.

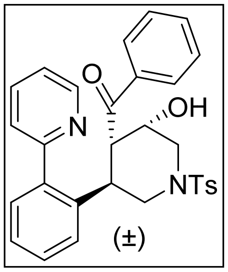

**(±)-((3S,4S,5R)-3-Hydroxy-5-(2-(pyridin-2-yl)phenyl)-1-tosylpiperidin-4-yl)(phenyl)methanone (3g):** A modification to the general procedure was used. ((*E*)-4-methyl-*N*-(4-oxo-4-phenylbut-2-en-1-yl)-*N*-(2-oxoethyl)benzenesulfonamide (71.5 mg, 0.200 mmol, 1.00 equiv), and 2-phenylpyridine (62.1 mg, 0.400 mmol, 2.00 equiv) was added, followed by the addition of a 95:5 acetic acid/water mixture (1.00 mL) instead of acetic acid. Chromatography eluting with a 40/60 solution of ethyl acetate/hexanes provided the product **3g** (46.1 mg, 45% yield) as an off-white solid (mp: 110-112 °C). IR (film): 3513, 3066, 1729, 1683, 1596, 1157, 753, 679 $cm^{-1}$; $^{1}H$ NMR (500 MHz, $CDCl_3$) δ 8.71 (d, *J* = 3.6 Hz, 1H), 7.83 (t, *J* = 7.6 Hz, 1H), 7.76 (d, *J* = 7.5 Hz, 2H), 7.65-7.62 (m, 3H), 7.51 (t, *J* = 7.4 Hz, 1H), 7.38 (t, *J* = 7.7 Hz, 2H), 7.32-7.29 (m, 4H), 7.22-7.16 (m, 3H), 4.30 (d, *J* = 11.1 Hz, 1H), 4.25 (d, *J* = 5.2 Hz, 1H), 3.98-3.89 (m, 3H), 2.71 (d, *J* = 12.2 Hz 1H), 2.53 (d, *J* = 7.0 Hz, 1H), 2.45 (s, 3H), 2.43-2.39 (m, 1H). $^{13}C\{^{1}H\}$ NMR ($CDCl_3$, 126 MHz): δ 199.30, 159.51, 149.43, 143.73, 142.17, 137.69, 136.73, 136.38, 133.91, 133.42, 130.59, 129.84, 128.86, 128.46, 128.18, 127.89, 127.14, 124.21, 122.26, 65.95, 51.90, 51.81, 51.75, 34.60, 21.71; HRMS (ESI/[M+H]+) calcd. For $C_{30}H_{28}N_2O_4S$: 513.1843 Found 513.1853.

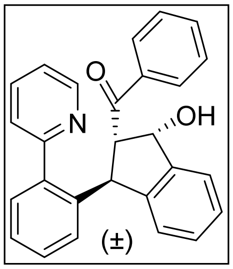

**(±)-((1S,2S,3R)-1-Hydroxy-3-(2-(pyridin-2-yl)phenyl)-2,3-dihydro-1*H*-inden-2-yl)(phenyl)methanone (3h):** A modification to the general procedure was used. A 2-5 mL microwave vial was charged with $[Cp^*RhCl_2]_2$[S1] (12.4 mg, 0.200 mmol, 1.00 equiv), $AgSbF_6$ (13.7 mg, 0.400 mmol, 2.00 equiv), (*E*)-2-(3-oxo-3-phenylprop-1-en-1-yl)benzaldehyde[S9] (47.3 mg, 0.20 mmol, 1.0 equiv), and 2-phenylpyridine (62.1 mg, 0.40 mmol, 2.0 equiv), followed by the addition of a 3:2 dioxane/water mixture (1.00 ml) instead of acetic acid. The reaction mixture was stirred at 40 °C instead of 50 °C for 20 h. Chromatography with a 1:1 solution of ethyl acetate/hexanes provided the product **3h** (39.0 mg, 50% yield) as a white solid (mp: 183-185 °C). IR (film): 3252, 3063, 1668, 1591, 1363, 1061, 754, 706 $cm^{-1}$; $^{1}H$ NMR (500 MHz, $CDCl_3$) δ 8.39 (d, *J* = 4.3 Hz, 1H), 7.80 (d, *J* = 7.6 Hz, 2H), 7.56 (t, *J* = 7.6 Hz, 1H), 7.51 (t, *J* = 7.4 Hz, 1H), 7.39-7.33 (m, 4H), 7.32-7.28 (m, 3H), 7.26-7.24 (m, 2H), 7.11 (dd, *J* = 7.6, 4.9 Hz, 1H), 7.06 (d, *J* = 7.0 Hz, 1H), 6.98 (d, *J* = 7.8 Hz, 1H), 5.47 (t, *J* = 6.1 Hz, 1H), 5.08 (d, *J* = 7.0 Hz, 1H), 4.40 (t, *J* = 6.2 Hz, 1H), 2.53 (s, 1H). $^{13}C\{^{1}H\}$ NMR ($CDCl_3$), 151 MHz): δ 199.98, 159.40, 148.82, 145.09, 143.14, 142.18, 141.19, 137.12, 136.24, 133.14, 129.91, 129.21, 129.09, 128.93, 128.48,

127.61, 126.85, 125.75, 124.28, 124.12, 121.87, 78.79, 67.05, 48.82; HRMS (ESI/[M+H]+) calcd. for $C_{27}H_{21}NO_2$: 392.1645 Found 392.1624.

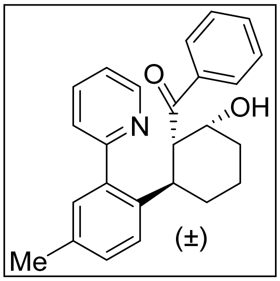


**(±)-((1S,2R,6R)-2-Hydroxy-6-(4-methyl-2-(pyridin-2-yl)phenyl)cyclohexyl)(phenyl)methanone (3i):** The general procedure using (*E*)-7-oxo-7-phenylhept-5-enal[S4] (40.5 mg, 0.200 mmol, 1.00 equiv), and 2-(*m*-tolyl)pyridine[S10] (67.7 mg, 0.400 mmol, 2.00 equiv) was followed. Chromatography eluting with a 40/60 solution of ethyl acetate/hexanes provided the product **3i** (70.5 mg, 95% yield) as a white solid (63-65 $^{\circ}$C). IR (film): 3495, 2929, 2859, 1681, 1588, 1200, 993, 700 cm$^{-1}$; $^{1}$H NMR (500 MHz, $CDCl_3$) δ 8.72 (d, *J* = 4.8 Hz, 1H), 7.81-7.79 (m, 3H), 7.55-7.50 (m, 2H), 7.38 (t, *J* = 7.8 Hz, 2H), 7.28 (ddd, *J* = 8.0, 5.2, 1.4 Hz, 1H), 7.20 (d, *J* = 8.0 Hz, 1H), 7.03 (s, 1H), 6.95 (d, *J* = 7.9 Hz, 1H), 4.18 (s, 1H), 4.00 (d, *J* = 11.4 Hz, 1H), 3.72 (dt, *J* = 11.8, 3.2 Hz, 1H), 2.90 (s, 1H), 2.20 (s, 3H), 1.98 (apparent t, *J* = 11.0 Hz, 2H), 1.82-1.73 (m, 1H), 1.61-1.53 (m, 2H), 1.52-1.46 (m, 1H). $^{13}$C{$^{1}$H} NMR ($CDCl_3$), 151 MHz): δ 204.83, 159.97, 149.38, 140.88, 138.57, 137.14, 136.10, 135.65, 133.33, 130.85, 129.04, 128.63, 128.37, 126.10, 124.54, 121.82, 67.05, 53.55, 35.27, 34.92, 31.99, 20.94, 19.91; HRMS (ESI/[M+H]+) calcd. for $C_{25}H_{25}NO_2$: 372.1958 Found 372.1938.

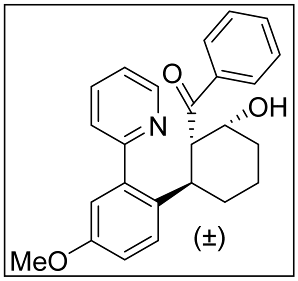


**(±)-((1S,2R,6R)-2-Hydroxy-6-(4-methoxy-2-(pyridin-2-yl)phenyl)cyclohexyl)(phenyl)methanone (3j):** The general procedure using (*E*)-7-oxo-7-phenylhept-5-enal[S4] (40.5 mg, 0.200 mmol, 1.00 equiv), and 2-(3-methoxyphenyl)pyridine[S10] (74.1 mg, 0.400 mmol, 2.00 equiv) was followed. Chromatography eluting with a 40/60 solution of ethyl acetate/hexanes provided the product **3j** (69.7 mg, 90% yield) as a white solid (mp: 59-61 $^{\circ}$C). IR (film): 3482, 2932, 1680, 1589, 1470, 1214, 791, 700 cm$^{-1}$; $^{1}$H NMR (500 MHz, $CDCl_3$) δ 8.72 (d, *J* = 4.2 Hz, 1H), 7.84-7.78 (m, 3H), 7.53-7.49 (m, 2H), 7.38 (t, *J* = 7.7 Hz, 2H), 7.30 (ddd, *J* = 7.5, 4.9, 1.2 Hz, 1H), 7.22 (d, *J* = 8.6 Hz, 1H), 6.74 (d, *J* = 2.7 Hz, 1H), 6.70 (dd, *J* = 8.6, 2.7 Hz, 1H), 4.17 (s, 1H), 3.96 (d, *J* = 11.4 Hz, 1H), 3.69 (s, 3H), 3.68-3.63 (m, 1H), 2.99 (s, 1H), 1.99-1.95 (m, 2H), 1.82-1.73 (m, 1H), 1.57-1.46 (m, 3H). $^{13}$C{$^{1}$H} NMR ($CDCl_3$), 151 MHz): δ 205.08, 159.67, 157.50, 149.32, 142.01, 137.18, 136.25, 133.79,

133.37, 128.64, 128.33, 127.45, 124.52, 122.04, 114.82, 114.67, 67.04, 55.36, 53.65, 35.13, 34.94, 31.95, 19.90; HRMS (ESI/[M+H]+) calcd. for $C_{25}H_{25}NO_3$: 388.1907 Found 388.1886.

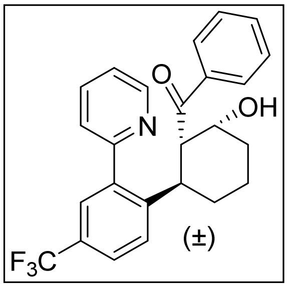

**(±)-((1S,2R,6R)-2-Hydroxy-6-(2-(pyridin-2-yl)-4-(trifluoromethyl)phenyl)cyclohexyl)(phenyl)methanone (3k):** The general procedure using (*E*)-7-oxo-7-phenylhept-5-enal[S4] (40.5 mg, 0.200 mmol, 1.00 equiv), and 2-(3-(trifluoromethyl)phenyl)pyridine[S10] (89.3 mg, 0.400 mmol, 2.00 equiv) was followed. Chromatography eluting with a 40/60 solution of ethyl acetate/hexanes provided the product **3k** (76.9 mg, 90% yield) as a white solid (mp: 64-66 °C). IR (film): 3483, 2935, 1681, 1337, 1165, 1117, 1079, 688 cm$^{-1}$; $^{1}$H NMR (500 MHz, $CDCl_3$) δ 8.74 (d, *J* = 4.8 Hz, 1H), 7.86 (dt, *J* = 7.7, 1.6 Hz, 1H), 7.79 (d, *J* = 7.4 Hz, 2H), 7.61 (d, *J* = 7.6 Hz, 1H), 7.53 (t, *J* = 7.4 Hz, 1H), 7.49 (s, 1H), 7.41-7.34 (m, 5H), 4.24 (s, 1H), 4.01 (d, *J* = 11.6 Hz, 1H), 3.81 (dt, *J* = 11.8, 3.2 Hz, 1H), 2.67 (s, 1H), 2.06-2.03 (m, 1H), 2.00-1.97 (m, 1H), 1.82-1.73 (m, 1H), 1.64-1.57 (m, 2H), 1.50 (apparent dq, *J* = 12.9, 3.4 Hz, 1H). $^{13}$C{$^{1}$H} NMR ($CDCl_3$), 151 MHz): δ 203.98, 158.49, 149.53, 146.10, 141.63, 136.87, 136.60, 133.64, 128.81, 128.42 (q, *J* = 32.6 Hz), 128.23, 127.22 (q, *J* = 3.7 Hz), 126.60, 125.03 (q, *J* = 3.6 Hz), 124.58, 124.057 (q, *J* = 272.0 Hz), 122.53, 67.06, 53.59, 35.70, 34.57, 32.04, 19.79; $^{19}$F NMR ($CDCl_3$, 470 MHz): δ -63.73 (s, 3F); HRMS (ESI/[M+H]+) calcd. for $C_{25}H_{22}F_3NO_2$: 426.1675 Found 426.1659.

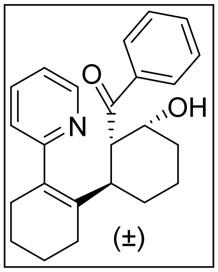

**(±)-((1R,2S,3R)-3-Hydroxy-2'-(pyridin-2-yl)-[1,1'-bi(cyclohexan)]-1'-en-2-yl)(phenyl)methanone (3l):** The general procedure using (*E*)-7-oxo-7-phenylhept-5-enal[S4] (40.5 mg, 0.200 mmol, 1.00 equiv), and 2-(cyclohex-1-en-1-yl)pyridine[S11] (63.7 mg, 0.400 mmol, 2.00 equiv) was followed. Chromatography eluting with a 40/60 solution of ethyl acetate/hexanes provided the product **3l** (57.9 mg, 80% yield) as a pale yellow solid (47-49 °C). IR (film): 3462, 2928, 2857, 1682, 1585, 1446, 1000, 780 cm$^{-1}$; $^{1}$H NMR (500 MHz, $CD_2Cl_2$) δ 8.58 (d, *J* = 4.8 Hz, 1H), 7.91 (d, *J* = 7.9 Hz, 2H), 7.70 (dt, *J* = 7.6, 1.8 Hz, 1H), 7.61 (t, *J* = 7.4 Hz, 1H), 7.51 (t, *J* = 7.7 Hz, 2H), 7.23 (d, *J* = 7.8 Hz, 1H), 7.16 (dd, *J* = 7.4, 5.0 Hz, 1H), 4.10 (s, 1H), 3.62 (d, *J* = 11.4 Hz, 1H), 3.20 (dt, *J* = 11.7, 2.7 Hz, 1H), 3.12 (s, 1H), 2.12-2.04 (m, 3H), 1.81 (apparent d, *J* = 13.6 Hz, 1H), 1.74-1.69 (m, 1H), 1.67-1.61 (m, 2H), 1.55-1.50 (m, 1H), 1.49-1.45 (m, 2H), 1.43-1.38 (m, 2H), 1.26-1.19 (m, 1H), 1.06-0.99 (m, 1H). $^{13}$C{$^{1}$H} NMR ($CD_2Cl_2$), 126 MHz): δ 205.98,

162.31, 149.45, 138.22, 136.08, 135.20, 134.98, 133.48, 129.03, 128.49, 123.61, 121.45, 67.11, 50.39, 37.94, 32.17, 31.10, 30.74, 25.06, 23.06, 22.77, 19.60; HRMS (ESI/[M+H]+) calcd. for $C_{24}H_{27}NO_2$: 362.2115 Found 362.2104.

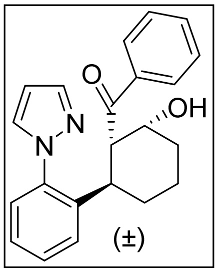


**(±)-((1S,2R,6R)-2-(2-(1*H*-Pyrazol-1-yl)phenyl)-6-hydroxycyclohexyl)(phenyl)methanone (3m):** The general procedure using (*E*)-7-oxo-7-phenylhept-5-enal[S4] (40.5 mg, 0.200 mmol, 1.00 equiv), and 1-phenyl-1*H*-pyrazole (57.7 mg, 0.400 mmol, 2.00 equiv) was followed. Chromatography eluting with a 40/60 solution of ethyl acetate/hexanes provided the product **3m** (57.0 mg, 82% yield) as a white solid (mp: 56-58 °C). IR (film): 3490, 2931, 1679, 1396, 1201, 938, 758, 694 cm$^{-1}$; $^1$H NMR (500 MHz, $CDCl_3$) δ 7.83-7.81 (m, 3H), 7.75 (s, 1H), 7.52 (t, *J* = 7.3 Hz, 1H), 7.40 (t, *J* = 7.4 Hz, 2H), 7.32 (d, *J* = 7.8 Hz, 1H), 7.19 (t, *J* = 7.6 Hz, 2H), 7.12 (t, *J* = 7.5 Hz, 1H), 6.50 (s, 1H), 4.23 (s, 1H), 4.00 (d, *J* = 11.5 Hz, 1H), 3.44 (dt, *J* = 11.7, 3.4 Hz, 1H), 2.64 (s, 1H), 2.02-1.97 (m, 2H), 1.85-1.76 (m, 1H), 1.61-1.56 (m, 2H), 1.48-1.41 (m, 1H). $^{13}C\{^1H\}$ NMR ($CDCl_3$), 126 MHz): δ 203.86, 140.68, 140.42, 139.85, 136.79, 133.53, 131.79, 128.84, 128.76, 128.31, 128.19, 127.50, 126.86, 106.33, 67.04, 53.38, 34.64, 33.88, 32.05, 19.83; HRMS (ESI/[M+H]+) calcd. for $C_{22}H_{22}N_2O_2$: 347.1754 Found 347.1747.

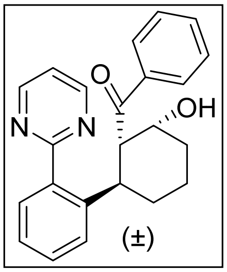


**(±)-((1S,2R,6R)-2-Hydroxy-6-(2-(pyrimidin-2-yl)phenyl)cyclohexyl)(phenyl)methanone (3n):** The general procedure using (*E*)-7-oxo-7-phenylhept-5-enal[S4] (40.5 mg, 0.200 mmol, 1.00 equiv), and 2-phenylpyrimidine (62.5 mg, 0.400 mmol, 2.00 equiv) was followed. Chromatography eluting with a 1:1 solution of ethyl acetate/hexanes provided the product **3n** (52.1 mg, 71% yield) as a white solid (mp: 120-122 °C). IR (film): 3242, 2930, 1685, 1557, 1414, 748, 685, 665 cm$^{-1}$; $^1$H NMR (500 MHz, $CDCl_3$) δ 8.90 (d, *J* = 4.8 Hz, 2H), 7.67 (d, *J* = 7.7 Hz, 2H), 7.55 (d, *J* = 7.6 Hz, 1H), 7.44 (t, *J* = 7.4 Hz, 1H), 7.38 (d, *J* = 7.8 Hz, 1H), 7.32-7.27 (m, 3H), 7.18 (t, *J* = 7.5 Hz, 1H), 7.09 (t, *J* = 7.5 Hz, 1H), 4.27 (t, *J* = 11.1 Hz, 1H), 4.18 (s, 1H), 3.91 (d, *J* = 10.6 Hz, 1H), 3.19 (s, 1H), 2.23 (apparent d, *J* = 11.1 Hz, 1H), 2.03-1.95 (m, 2H), 1.70-1.63 (m, 2H), 1.60-1.57 (m, 1H). $^{13}C\{^1H\}$ NMR ($CDCl_3$), 151 MHz): δ 205.25, 167.75, 156.99, 142.58, 138.84, 137.28, 133.14, 130.87, 129.28, 128.42, 128.24, 128.06, 126.21, 118.76, 67.03, 53.96, 35.45, 33.97, 32.02, 19.89; HRMS (ESI/[M+H]+) calcd. for $C_{23}H_{22}N_2O_2$: 359.1754 Found 359.1744.

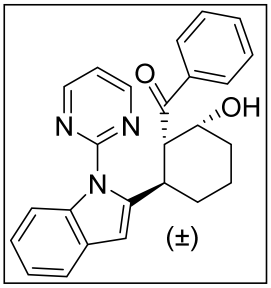

**(±)-((1S,2R,6R)-2-Hydroxy-6-(1-(pyrimidin-2-yl)-1*H*-indol-2-yl)cyclohexyl)(phenyl)methanone (3o):** The general procedure using (*E*)-7-oxo-7-phenylhept-5-enal[S4] (40.5 mg, 0.200 mmol, 1.00 equiv), and 1-(pyrimidin-2-yl)-1*H*-indole[S12] (78.1 mg, 0.400 mmol, 2.00 equiv) was followed. Chromatography eluting with a 1:1 solution of ethyl acetate/hexanes provided the product **3o** (38.4 mg, 48% yield) as an off-white solid (68-70 °C). IR (film): 3462, 2932, 1679, 1563, 1454, 1420, 746, 697 cm$^{-1}$; $^{1}$H NMR (500 MHz, $CDCl_3$) δ 8.85 (d, *J* = 4.8 Hz, 2H), 7.99 (d, *J* = 8.2 Hz, 1H), 7.79 (d, *J* = 7.6 Hz, 2H), 7.45 (t, *J* = 7.4 Hz, 1H), 7.33 (apparent t, *J* = 7.9 Hz, 3H), 7.21 (t, J = 4.8 Hz, 1H), 7.10 (t, *J* = 7.6 Hz, 1H), 7.05 (t, *J* = 7.3 Hz, 1H), 6.40 (s, 1H), 4.65 (dt, *J* = 11.7, 3.2 Hz, 1H), 4.25 (s, 1H), 3.97 (d, *J* = 11.5 Hz, 1H), 3.01 (s, 1H), 2.32 (apparent d, *J* = 13.8 Hz, 1H), 2.07-1.95 (m, 2H), 1.67-1.58 (m, 3H). $^{13}$C{$^{1}$H} NMR ($CDCl_3$), 126 MHz): δ 204.37, 158.35, 158.12, 144.95, 136.70, 136.67, 133.40, 128.91, 128.58, 128.31, 122.70, 121.62, 119.76, 117.62, 113.53, 104.06, 67.20, 54.40, 33.98, 32.72, 32.12, 19.91; HRMS (ESI/[M+H]+) calcd. for $C_{25}H_{23}N_3O_2$: 398.1863 Found 398.1848.

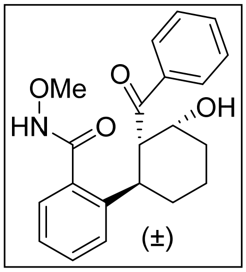

**(±)-2-((1R,2S,3R)-2-Benzoyl-3-hydroxycyclohexyl)-*N*-methoxybenzamide (3p):** A modification to the general procedure was used. (*E*)-7-oxo-7-phenylhept-5-enal[S4] (40.5 mg, 0.200 mmol, 1.00 equiv), and *N*-methoxybenzamide[S13] (60.5 mg, 0.400 mmol, 2.00 equiv) was added, followed by the addition of a 3:2 mixture of dioxane/water (0.500 mL) instead of acetic acid. Chromatography eluting with a 70/30 solution of ethyl acetate/hexanes provided the product **3p** (35.4 mg, 50% yield) as a white solid (mp: 71-73 °C). IR (film): 3435, 3212, 2933, 1658, 1597, 1447, 1030, 690 cm$^{-1}$; $^{1}$H NMR (500 MHz, $CDCl_3$) δ 10.51 (s, 1H), 7.88 (d, *J* = 7.7 Hz, 2H), 7.57 (t, *J* = 7.1 Hz, 1H), 7.45 (t, *J* = 7.4 Hz, 2H), 7.41 (d, *J* = 7.4 Hz, 1H), 7.24-7.20 (m, 2H), 7.14 (t, *J* = 7.0 Hz, 1H), 4.38 (s, 1H), 4.07 (d, *J* = 10.2 Hz, 1H), 4.02 (s, 3H), 3.83 (t, *J* = 10.8 Hz, 1H), 2.00 (apparent t, *J* = 15.9 Hz, 2H), 1.91-1.88 (m, 1H), 1.74 (apparent t, *J* = 13.8 Hz, 1H), 1.66-1.63 (m, 2H), 1.51-1.44 (m, 1H). $^{13}$C{$^{1}$H} NMR ($CDCl_3$), 126 MHz): δ 204.02, 167.94, 141.81, 136.09, 134.18, 133.99, 130.49, 128.99, 128.58, 128.51, 126.65, 125.41, 67.83, 64.37, 55.34, 35.34, 35.21, 32.84, 19.93; HRMS (ESI/[M+H]+) calcd. for $C_{21}H_{23}NO_4$: 354.1700 Found 354.1706.

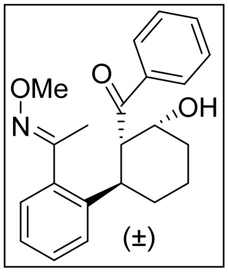

**(±)-((1S,2R,6R)-2-Hydroxy-6-(2-((*E*)-1-(methoxyimino)ethyl)phenyl)cyclohexyl)(phenyl)methanone (3q):** A modification to the general procedure was used. (*E*)-7-oxo-7-phenylhept-5-enal[S4] (40.5 mg, 0.200 mmol, 1.00 equiv), and (*E*)-1-phenylethan-1-one *O*-methyl oxime[S14] (59.7 mg, 0.400 mmol, 2.00 equiv) was added, followed by the addition of a 3:2 mixture of dioxane/water (1.00 mL) instead of acetic acid. Chromatography eluting with a 20/80 solution of ethyl acetate/hexanes provided the product **3q** (24.2 mg, 34% yield) as a waxy white solid. IR (film): 3462, 2934, 1682, 1597, 1446, 1201, 1041, 752 $cm^{-1}$; $^{1}H$ NMR (500 MHz, $CDCl_3$) δ 7.77 (d, *J* = 7.8 Hz, 2H), 7.51-7.46 (m, 1H), 7.36 (t, *J* = 7.6 Hz, 2H), 7.23 (d, *J* = 7.7 Hz, 1H), 7.07-7.00 (m, 3H), 4.26 (s, 1H), 4.02-3.99 (m, 1H), 3.99 (s, 3H), 3.75 (dt, *J* = 11.8, 3.3 Hz, 1H), 3.08 (s, 1H), 2.28 (s, 3H), 2.06 (apparent d, *J* = 12.7 Hz, 2H), 2.01-1.94 (m, 1H), 1.65-1.60 (m, 2H), 1.55-1.47 (m, 1H). $^{13}C\{^{1}H\}$ NMR ($CDCl_3$), 151 MHz): δ 204.57, 157.06, 141.97, 137.82, 137.10, 133.38, 128.65, 128.55, 128.38, 128.31, 128.26, 126.24, 67.05, 61.89, 53.45, 36.26, 34.97, 32.08, 19.85, 16.85; HRMS (ESI/[M+H]+) calcd. for $C_{22}H_{25}NO_3$: 352.1907 Found 352.1905.

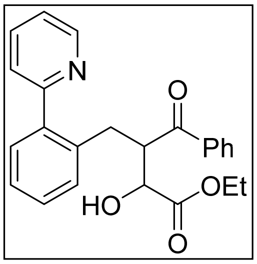

**Ethyl-2-hydroxy-4-oxo-4-phenyl-3-(2-(pyridin-2-yl)benzyl)butanoate (3r):** In a $N_2$-filled glove box, a 2-5 mL microwave vial was charged with $AgSbF_6$ (13.8 mg, 0.0402 mmol, 0.200 equiv), $[Cp^*RhCl_2]_2$[S1] (6.2 mg, 0.010 mmol, 0.050 equiv), 2-phenylpyridine (31.0 mg, 0.200 mmol, 1.0 equiv), phenyl vinyl ketone[S15] (29.1 mg, 0.220 mmol, 1.1 equiv), and ethyl glyoxylate (40.8 mg, 0.400 mmol, 2.0 equiv). Acetic acid (0.1 mL) was then added and the vial was equipped with a stir bar. The reaction vial was sealed and then outside the glove box, the vial was heated at 40 °C in a preset oil bath for 20 h with stirring. The reaction mixture was then filtered over a plug of celite (1 cm celite in a glass pipette) eluting with $CH_2Cl_2$ and concentrated. Chromatography eluting with a 40/60 solution of ethyl acetate/hexanes provided the product as a mixture of diastereomers **3r** as a colorless oil. The mixture was then separated by preparatory TLC using 70/30 ethyl ether/pentane to obtain the diastereomer with higher $R_f$ (31.7 mg) as a colorless oil and the diastereomer with lower $R_f$ (31.5 mg) as a colorless oil (63.2 mg total, 1:1 dr, 81% yield).

Diastereomer 1 (Higher $R_f$): IR (film): 3527, 3064, 2983, 1741, 1678, 1208, 1024, 754 $cm^{-1}$; $^1H$ NMR (500 MHz, $CDCl_3$) δ 8.67 (d, $J$ = 4.3 Hz, 1H), 7.82 (dt, $J$ = 7.7, 1.5 Hz, 1H), 7.67 (d, $J$ = 7.4 Hz, 2H), 7.53-7.45 (m, 3H), 7.37-7.30 (m, 6H), 4.27 (dt, $J$ = 7.7, 3.2 Hz, 1H), 4.24 (d, $J$ = 3.2 Hz, 1H), 3.90 (q, $J$ = 6.8 Hz, 2H), 3.26 (d, $J$ = 7.6 Hz, 2H), 1.03 (t, $J$ = 7.1 Hz, 3H). $^{13}C\{^1H\}$ NMR ($CDCl_3$, 126 MHz): δ 203.33, 173.23, 159.89, 148.87, 140.39, 137.28, 136.97, 136.51, 133.32, 131.43, 130.54, 128.95, 128.57, 128.56, 127.11, 124.71, 122.23, 71.03, 61.33, 50.42, 31.59, 14.02; HRMS (ESI/[M+H]+) calcd. for $C_{24}H_{23}NO_4$: 390.1700 Found 390.1701.

Diastereomer 2 (Lower $R_f$): IR (film): 3507, 3062, 2983, 1729, 1679, 1206, 1024, 726 $cm^{-1}$; $^1H$ NMR (500 MHz, $CDCl_3$) δ 8.66 (d, $J$ = 4.5 Hz, 1H), 7.96 (d, $J$ = 7.7 Hz, 2H), 7.83 (dt, $J$ = 7.8, 1.8 Hz, 1H), 7.55 (t, $J$ = 7.3 Hz, 1H), 7.46-7.43 (m, 3H), 7.39 (d, $J$ = 7.8 Hz, 1H), 7.34-7.31 (m, 3H), 7.28-7.27 (m, 1H), 6.77 (s, 1H), 4.64 (d, $J$ = 4.2 Hz, 1H), 4.49 (dt, $J$ = 9.3, 4.5 Hz, 1H), 3.79 (q, $J$ = 7.1 Hz, 2H), 3.42 (dd, $J$ = 14.5, 9.7 Hz, 1H), 3.03 (dd, $J$ = 14.5, 4.8 Hz, 1H), 1.08 (t, $J$ = 7.1 Hz, 3H). $^{13}C\{^1H\}$ NMR ($CDCl_3$, 126 MHz): δ 201.10, 173.25, 159.46, 148.13, 140.01, 137.63, 136.70, 136.23, 133.18, 131.06, 130.50, 128.79, 128.73, 128.58, 126.83, 124.99, 122.25, 71.97, 61.46, 51.76, 29.87, 13.99; HRMS (ESI/[M+H]+) calcd. for $C_{24}H_{23}NO_4$: 390.1700 Found 390.1710.

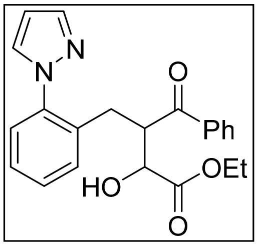


**Ethyl-3-(2-(1H-pyrazol-1-yl)benzyl)-2-hydroxy-4-oxo-4-phenylbutanoate (3s):**

In a $N_2$-filled glove box, a 2-5 mL microwave vial was charged with $AgSbF_6$ (13.8 mg, 0.0402 mmol, 0.201 equiv), $[Cp^*RhCl_2]_2$[S1] (6.2 mg, 0.010 mmol, 0.050 equiv), 1-phenylpyrazole (28.8 mg, 0.200 mmol, 1.00 equiv), phenyl vinyl ketone[S15] (29.1 mg, 0.220 mmol, 1.10 equiv), and ethyl glyoxylate (40.8 mg, 0.400 mmol, 2.00 equiv). Acetic acid (0.1 mL) was then added and the vial was equipped with a stir bar. The reaction vial was sealed and then outside the glove box, the vial was heated at 50 °C in a preset oil bath for 20 h with stirring. The reaction mixture was then filtered over a plug of celite (1 cm celite in a glass pipette) eluting with $CH_2Cl_2$ and concentrated. Chromatography eluting with a 30/70 solution of ethyl acetate/hexanes provided two separate diastereomers, with the higher $R_f$ diastereomer pure as a colorless waxy solid (26.2 mg). The lower $R_f$ diastereomer was further purified by preparatory TLC using 4:1 dichloromethane/diethyl ether to obtain the lower $R_f$ diastereomer (25.9 mg) as a colorless waxy solid (52.1 mg total, 1:1 dr, 69% yield).

Diastereomer 1 (Higher $R_f$): IR (film): 3490, 1739, 1679, 1394, 1211, 1022, 938, 758 $cm^{-1}$; $^1H$ NMR (500 MHz, $CDCl_3$) δ 7.77-7.76 (m, 1H), 7.72 (d, *J* = 7.5 Hz, 2H), 7.65 (d, *J* = 1.9 Hz, 1H), 7.54-7.51 (m, 2H), 7.37 (t, *J* = 7.7 Hz, 2H), 7.35-7.29 (m, 2H), 7.28-7.27 (m, 1H), 6.51 (t, *J* = 2.1 Hz, 1H), 4.18-4.15 (m, 2H), 4.12-4.11 (m, 1H), 3.94 (q, *J* = 7.1 Hz, 2H), 3.18 (dd, *J* = 13.5, 5.6 Hz, 1H), 3.07 (dd, J = 13.5, 9.7 Hz, 1H), 1.04 (t, *J* = 7.1 Hz, 3H). $^{13}C\{^1H\}$ NMR ($CDCl_3$, 126 MHz): δ 203.84, 173.13, 140.89, 140.00, 136.54, 134.20, 133.60, 132.57, 131.08, 128.94, 128.81, 128.57, 127.90, 126.74, 106.95, 71.10, 61.48, 49.07, 31.07, 14.01; HRMS (ESI/[M+H]+) calcd. for $C_{22}H_{22}N_2O_4$: 379.1652 Found 379.1641.

Diastereomer 2 (Lower $R_f$): IR (film): 3492, 1729, 1677, 1395, 1211, 1022, 938, 759 $cm^{-1}$; $^1H$ NMR (500 MHz, $CDCl_3$) δ 7.83 (d, *J* = 7.4 Hz, 2H), 7.75 (d, *J* = 1.9 Hz, 1H), 7.62 (d, *J* = 1.8 Hz, 1H), 7.52 (t, *J* = 7.4 Hz, 1H), 7.40 (t, *J* = 7.7 Hz, 2H), 7.37-7.35 (m, 1H), 7.28-7.25 (m, 2H), 7.24-7.21 (m, 1H), 6.48 (t, *J* = 2.2 Hz, 1H), 4.45 (d, *J* = 4.5 Hz, 1H), 4.31 (s, 1H), 4.23 (dt, *J* = 7.3, 4.8 Hz, 1H), 4.01-3.89 (m, 2H), 3.18 (dd, *J* = 14.2, 7.9 Hz, 1H), 3.12 (dd, *J* = 14.2, 6.8 Hz, 1H), 1.13 (t, *J* = 7.1 Hz, 3H). $^{13}C\{^1H\}$ NMR ($CDCl_3$, 126 MHz): δ 201.10, 173.11, 140.72, 139.94, 136.48, 134.65, 133.32, 132.05, 131.14, 128.72, 128.70, 128.59, 127.59, 126.55, 106.88, 71.44, 61.87, 50.74, 29.58, 14.02; HRMS (ESI/[M+H]+) calcd. for $C_{22}H_{22}N_2O_4$: 379.1652 Found 379.1639.

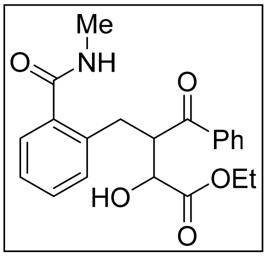


**Ethyl-2-hydroxy-3-(2-(methylcarbamoyl)benzyl)-4-oxo-4-phenylbutanoate (3t):** In a $N_2$-filled glove box, a 2-5 mL microwave vial was charged with $AgSbF_6$ (13.8 mg, 0.0402 mmol, 0.201 equiv), $[Cp^*RhCl_2]_2$[S1] (6.2 mg, 0.010 mmol, 0.050 equiv), N-methylbenzamide (27.1 mg, 0.200 mmol, 1.00 equiv), phenyl vinyl ketone[S15] (29.1 mg, 0.220 mmol, 1.10 equiv), and ethyl glyoxylate (40.8 mg, 0.400 mmol, 2.00 equiv). Acetic acid (0.1 mL) was then added and the vial was equipped with a stir bar. The reaction vial was sealed and then outside the glove box, the vial was heated at 50 °C in a preset oil bath for 20 h with stirring. The reaction mixture was then filtered over a plug of celite (1 cm celite in a glass pipette) eluting with $CH_2Cl_2$ and concentrated. Chromatography eluting with a 95:5 solution of methyl *tert*-butyl ether/hexanes provided the product as a mixture of diastereomers **3t** as a colorless waxy solid. The mixture was then separated by preparatory TLC using 2:1 dichloromethane/ethyl acetate to obtain the diastereomer with higher $R_f$ (20.1 mg) as a colorless waxy solid and the diastereomer with lower $R_f$ (19.9 mg) as a colorless waxy solid (40.0 mg total, 1:1 dr, 54% yield).

Diastereomer 1 (Higher $R_f$): IR (film): 3348, 1737, 1632, 1597, 1447, 1210, 1093, 690 $cm^{-1}$; $^1H$ NMR (500 MHz, $CDCl_3$) δ 7.97 (d, *J* = 7.6 Hz, 2H), 7.54 (t, *J* = 7.3 Hz, 1H), 7.43 (t, *J* = 7.7 Hz, 2H), 7.38 (t, *J* = 7.7 Hz, 2H), 7.29 (t, *J* = 7.4 Hz, 1H), 7.21 (t, *J* = 7.4 Hz, 1H), 6.21 (s, 1H), 4.53 (t, *J* = 6.3 Hz, 1H), 4.21 (s, 1H), 4.15 (s, 1H), 4.02-3.93 (m, 2H), 3.28 (dd, *J* = 13.5, 6.4 Hz, 1H), 3.16 (dd, J = 13.5, 9.3 Hz, 1H), 3.01 (d, *J* = 4.8 Hz, 3H), 1.05 (t, *J* = 7.1 Hz, 3H). $^{13}C\{^1H\}$ NMR ($CDCl_3$, 126 MHz): δ 203.98, 173.11, 170.77, 137.15, 136.79, 136.33, 133.65, 131.57, 130.22, 128.89, 128.72, 127.39, 127.06, 71.29, 61.53, 50.11, 32.58, 26.89, 14.06; HRMS (ESI/[M+H]+) calcd. for $C_{21}H_{23}NO_5$: 370.1649 Found 370.1643.

Diastereomer 2 (Lower $R_f$): IR (film): 3371, 1737, 1631, 1597, 1447, 1210, 1096, 689 $cm^{-1}$; $^1H$ NMR (500 MHz, $CDCl_3$) δ 7.92 (d, *J* = 7.3 Hz, 2H), 7.52 (t, *J* = 7.4 Hz, 1H), 7.41 (t, *J* = 7.7 Hz, 2H), 7.31 (d, *J* = 7.5 Hz, 1H), 7.22 (d, *J* = 3.9 Hz, 2H), 7.17-7.13 (m, 1H), 6.31 (s, 1H), 4.55-4.50 (m, 2H), 4.19 (s, 1H), 3.98-3.87 (m, 2H), 3.26 (dd, *J* = 14.0, 7.0 Hz, 1H), 3.20 (dd, *J* = 14.0, 7.0 Hz, 1H), 3.00 (d, *J* = 4.9 Hz, 3H), 1.13 (t, *J* = 7.2 Hz, 3H). $^{13}C\{^1H\}$ NMR ($CDCl_3$, 126 MHz): δ 201.47, 173.00, 170.93, 137.19, 136.65, 133.38, 131.17, 129.87, 128.76, 128.72, 127.31, 126.81, 71.57, 61.90, 51.40, 30.94, 26.88, 14.02; HRMS (ESI/[M+H]+) calcd. for $C_{21}H_{23}NO_5$: 370.1649 Found 370.1648.

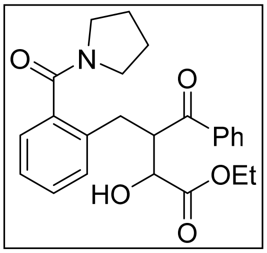


**Ethyl-2-hydroxy-4-oxo-4-phenyl-3-(2-(pyrrolidine-1-carbonyl)benzyl)butanoate (3u):** In a $N_2$-filled glove box, a 2-5 mL microwave vial was charged with $AgSbF_6$ (13.8 mg, 0.0402 mmol, 0.200 equiv), $[Cp^*RhCl_2]_2$[S1] (6.2 mg, 0.010 mmol, 0.050 equiv), phenyl(pyrrolidin-1-yl)methanone[S16] (35.1 mg, 0.200 mmol, 1.00 equiv), phenyl vinyl ketone[S15] (29.1 mg, 0.220 mmol, 1.10 equiv), and ethyl glyoxylate (40.8 mg, 0.400 mmol, 2.00 equiv). Acetic acid (0.1 mL) was then added and the vial was equipped with a stir bar. The reaction vial was sealed and then outside the glove box, the vial was heated at 60 °C in a preset oil bath for 20 h with stirring. The reaction mixture was then filtered over a plug of celite (1 cm celite in a glass pipette) eluting with $CH_2Cl_2$ and concentrated. Chromatography eluting with a 1:1 solution of ethyl acetate/hexanes provided the product as a mixture of diastereomers **3u** as a colorless waxy solid. The mixture was then separated by preparatory TLC using 4:1 dichloromethane/ethyl acetate to obtain the diastereomer with higher $R_f$ (32.9 mg) as a colorless

waxy solid and the diastereomer with lower $R_f$ (27.4 mg) as a colorless waxy solid (60.3 mg total, 1.2:1 dr, 74% yield).

Diastereomer 1 (Higher $R_f$): IR (film): 3400, 1742, 1681, 1615, 1595, 1448, 1210, 729 $cm^{-1}$; $^1H$ NMR (500 MHz, $CDCl_3$) δ 8.00 (d, *J* = 7.6 Hz, 2H), 7.55 (t, *J* = 7.3 Hz, 1H), 7.46-7.42 (m, 3H), 7.29-7.24 (m, 3H), 4.44-4.40 (m, 1H), 4.18 (s, 1H), 4.02-3.93 (m, 2H), 3.72 (dt, *J* = 13.3, 6.9 Hz, 1H), 3.64 (dt, *J* = 12.5, 6.8 Hz, 1H), 3.16-3.04 (m, 4H), 1.99-1.81 (m, 4H), 1.03 (t, *J* = 7.1 Hz, 3H). $^{13}C\{^1H\}$ NMR ($CDCl_3$, 126 MHz): δ 203.91, 173.17, 169.80, 138.06, 136.63, 134.69, 133.64, 131.55, 129.23, 128.98, 128.73, 127.09, 126.39, 70.91, 61.44, 49.87, 48.93, 45.76, 32.60, 26.17, 24.69, 14.05; HRMS (ESI/[M+H]+) calcd. for $C_{24}H_{27}NO_5$: 410.1962 Found 410.1967.

Diastereomer 2 (Lower $R_f$): IR (film): 3326, 1732, 1677, 1610, 1595, 1447, 1209, 727 $cm^{-1}$; $^1H$ NMR (500 MHz, $CDCl_3$) δ 7.94 (d, *J* = 7.8 Hz, 2H), 7.50 (t, *J* = 7.4 Hz, 1H), 7.40 (t, *J* = 7.6 Hz, 2H), 7.20-7.15 (m, 4H), 4.49 (t, *J* = 5.9 Hz, 1H), 4.46-4.42 (m, 1H), 4.18 (d, *J* = 6.6 Hz, 1H), 4.03-3.92 (m, 2H), 3.74-3.68 (m, 1H), 3.66-3.61 (m, 1H), 3.20-3.07 (m, 4H), 2.04-1.91 (m, 2H), 1.89-1.83 (m, 2H), 1.11 (t, *J* = 7.2 Hz, 3H). $^{13}C\{^1H\}$ NMR ($CDCl_3$, 126 MHz): δ 201.30, 173.12, 169.96, 137.76, 136.67, 135.27, 133.28, 131.07, 129.00, 128.79, 128.72, 126.74, 126.29, 71.70, 61.86, 51.47, 48.97, 45.78, 31.52, 26.18, 24.69, 13.99; HRMS (ESI/[M+H]+) calcd. for $C_{24}H_{27}NO_5$: 410.1962 Found 410.1958

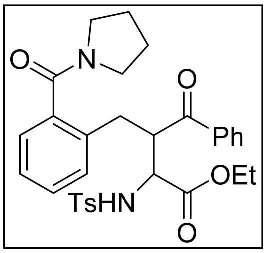


**Ethyl-2-((4-methylphenyl)sulfonamido)-4-oxo-4-phenyl-3-(2-(pyrrolidine-1-carbonyl)benzyl)butanoate (3v):** In a $N_2$-filled glove box, a 2-5 mL microwave vial was charged with crushed 3Å molecular sieves (50.0 mg), $AgSbF_6$ (13.8 mg, 0.0402 mmol, 0.201 equiv), $[Cp^*RhCl_2]_2$[S1] (6.2 mg, 0.010 mmol, 0.050 equiv), phenyl(pyrrolidin-1-yl)methanone[S16] (35.1 mg, 0.200 mmol, 1.00 equiv), phenyl vinyl ketone[S15] (29.1 mg, 0.220 mmol, 1.10 equiv), and (*E*)-ethyl-2-(tosylimino)acetate[S17] (102.1 mg, 0.4003 mmol, 2.002 equiv). 1,2-dichloroethane (0.4 mL) was then added, and the vial was equipped with a stir bar. The reaction vial was sealed and then outside the glove box, the vial was heated at 60 °C in a preset oil bath for 20 h with stirring. The reaction mixture was then filtered over a plug of celite (1 cm celite in a glass pipette) eluting with $CH_2Cl_2$ and concentrated. Chromatography eluting with a 95:5 solution of methyl *tert*-butyl ether/hexanes provided the product as a mixture of diastereomers **3v** as a colorless waxy solid. The mixture was then separated by preparatory TLC using 2:1

dichloromethane/ethyl acetate to obtain the diastereomer with higher $R_f$ (53.5 mg) as a white solid (mp: 165-167 °C), and the diastereomer with lower $R_f$ (26.8 mg) as a colorless waxy solid (80.3 mg total, 2:1 dr, 71% yield).

Diastereomer 1 (Higher $R_f$): IR (film): 2979, 2891, 1750, 1671, 1609, 1164, 1092, 702 cm$^{-1}$; $^1$H NMR (500 MHz, $CDCl_3$) δ 7.75-7.71 (m, 4H), 7.43 (t, *J* = 7.2 Hz, 1H), 7.28-7.25 (m, 4H), 7.21 (d, *J* = 7.5 Hz, 1H), 7.16-7.13 (m, 1H), 7.09-7.06 (m, 2H), 6.34 (d, *J* = 9.5 Hz, 1H), 4.56 (dt, *J* = 8.8, 5.0 Hz, 1H), 4.26 (dd, *J* = 9.4, 4.0 Hz, 1H), 3.78-3.72 (m, 1H), 3.67-3.65 (m, 3H), 3.23-3.10 (m, 3H), 3.00 (dd, *J* = 13.8, 5.4 Hz, 1H), 2.39 (s, 3H), 2.06-1.99 (m, 1H), 1.97-1.86 (m, 3H), 0.80 (t, *J* = 7.1 Hz, 3H). $^{13}$C{$^1$H} NMR ($CDCl_3$, 126 MHz): δ 202.57, 169.80, 169.78, 143.35, 137.77, 137.66, 136.81, 134.71, 133.46, 131.43, 129.55, 129.02, 128.73, 128.52, 127.40, 126.94, 126.27, 61.66, 57.46, 49.03, 48.29, 45.83, 33.37, 26.17, 24.68, 21.65, 13.67; HRMS (ESI/[M+H]+) calcd. for $C_{31}H_{34}N_2O_6S$: 563.2210 Found 563.2189.

Diastereomer 2 (Lower $R_f$): IR (film): 2973, 2873, 1735, 1671, 1597, 1155, 1092, 664 cm$^{-1}$; $^1$H NMR (500 MHz, $CDCl_3$) δ 7.71-7.67 (m, 4H), 7.41 (t, *J* = 7.7 Hz, 1H), 7.28-7.25 (m, 2H), 7.23-7.18 (m, 3H), 7.12 (t, *J* = 7.4 Hz, 1H), 7.04-6.98 (m, 2H), 6.01 (d, *J* = 9.1 Hz, 1H), 4.45 (dt, *J* = 9.0, 4.5 Hz, 1H), 4.26 (t, *J* = 8.6 Hz, 1H), 3.91-3.87 (m, 1H), 3.69-3.65 (m, 1H), 3.57 (q, *J* = 7.1 Hz, 2H), 3.28 (dd, *J* = 13.7, 4.2 Hz, 1H), 3.22-3.18 (m, 1H), 3.16-3.12 (m, 1H), 3.06 (dd, *J* = 13.7, 9.6 Hz, 1H), 2.38 (s, 3H), 2.13-2.07 (m, 1H), 1.98-1.88 (m, 3H), 0.79 (t, *J* = 7.1 Hz, 3H). $^{13}$C{$^1$H} NMR ($CDCl_3$, 126 MHz): δ 200.83, 170.06, 169.93, 143.43, 137.99, 137.50, 137.09, 134.89, 133.22, 131.09, 129.57, 128.84, 128.70, 128.48, 127.47, 126.85, 126.30, 61.77, 57.84, 50.38, 49.06, 45.85, 33.74, 26.23, 24.73, 21.60, 13.58; HRMS (ESI/[M+H]+) calcd. for $C_{31}H_{34}N_2O_6S$: 563.2210 Found 563.2216.

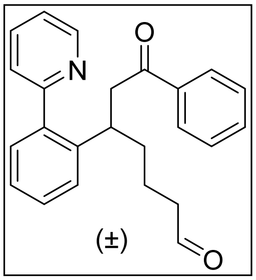


**(±)-7-oxo-7-phenyl-5-(2-(pyridin-2-yl)phenyl)heptanal (9a):** In a $N_2$-filled glove box, a 2-5 mL microwave vial was charged with $AgSbF_6$ (27.5 mg, 0.0800 mmol, 0.10 equiv), $[Cp^*RhCl_2]_2$[S1] (12.4 mg, 0.0201 mmol, 0.025 equiv), (*E*)-7-oxo-7-phenylhept-5-enal[S4] (161.8 mg, 0.8000 mmol, 1.0 quiv), and 2-phenylpyridine (248.3 mg, 1.600 mmol, 2.0 equiv). A 3:2 mixture of dioxane/water (4.0 mL) was then added and the vial was equipped with a stir bar. The reaction vial was sealed and then outside the glove box, the vial was heated at 25 °C in a preset oil bath for 20 h with stirring. The reaction mixture was then filtered

over a plug of celite (3 cm celite in a glass pipette) with $CH_2Cl_2$ and concentrated. Chromatography eluting with a 1:1 solution of ethyl acetate/hexanes provided the product **9a** (25.8 mg, 9% yield) as a colorless oil. IR (film): 2931, 1720, 1679, 1584, 1425, 988, 751, 691 $cm^{-1}$; $^1H$ NMR (400 MHz, $CDCl_3$) δ 9.59 (s, 1H), 8.63 (d, *J* = 4.9 Hz, 1H), 7.87 (d, *J* = 7.7 Hz, 2H), 7.73 (t, *J* = 7.3 Hz, 1H), 7.52 (t, *J* = 7.5 Hz, 1H), 7.41-7.38 (m, 5H), 7.33-7.28 (m, 2H), 7.25-7.23 (m, 1H), 3.65 (tt, *J* = 9.3, 5.1 Hz, 1H), 3.43 (dd, *J* = 16.1, 5.2 Hz, 1H), 3.15 (dd, *J* = 16.0, 8.7 Hz, 1H), 2.21 (t, *J* = 7.0 Hz, 2H), 1.73-1.61 (m, 2H), 1.49-1.32 (m, 2H). $^{13}C\{^1H\}$ NMR ($CDCl_3$, 101 MHz): δ 202.69, 199.14, 160.18, 149.15, 142.27, 141.13, 137.04, 136.44, 133.04, 130.06, 128.94, 128.62, 128.30, 126.41, 126.35, 124.62, 121.95, 46.27, 43.69, 36.09, 35.37, 20.00; HRMS (ESI/[M+H]+) calcd. for $C_{24}H_{23}NO_2$: 358.1802 Found 358.1799.

## IV. Control Experiments with Intermediate 9a

**Table 4, entry 1:**

In a $N_2$-filled glove box, a 2-5 mL microwave vial was charged with $AgSbF_6$ (1.7 mg, 0.0049 mmol, 0.10 equiv), $[Cp^*RhCl_2]_2$[S1] (0.8 mg, 0.001 mmol, 0.025 equiv), and **9a** (17.9 mg, 0.0501 mmol, 1.0 equiv). A 3:2 mixture of dioxane/$H_2O$ (0.025 mL) was then added, and the vial was equipped with a stir bar. The reaction vial was sealed, and then outside the glove box, the vial was heated at 50 $^{\circ}$C in a preset oil bath for 20 h with stirring. The reaction mixture was then concentrated, and 13.2 mg (0.0785 mmol) of an external standard (1,3,5-trimethoxybenzene) was added to the reaction mixture. $CDCl_3$ was added to the reaction mixture, and the crude NMR was taken to determine that 11% of compound **3a** was formed along with 1% of recovered **9a**.

**Table 4, entry 2:**

In a $N_2$-filled glove box, a 2-5 mL microwave vial was charged with **9a** (36.0 mg, 0.101 mmol, 1.0 equiv). A 3:2 mixture of dioxane/$H_2O$ (0.05 mL) was then added, and the vial was equipped with a stir bar. The reaction vial was sealed, and then outside the glove box, the vial was heated at 50 $^{\circ}$C in a preset oil bath for 20 h with stirring. The reaction mixture was then concentrated, and 10.0 mg (0.0595 mmol) of an external standard (1,3,5-trimethoxybenzene) was added to the reaction mixture. $CDCl_3$ was added to the reaction mixture, and the crude NMR was taken to determine that 12% of compound **3a** was formed along with 1% of recovered **9a**.

**Table 4, entry 3:**

In a $N_2$-filled glove box, a 2-5 mL microwave vial was charged with $AgSbF_6$ (1.7 mg, 0.0049 mmol, 0.10 equiv), $[Cp^*RhCl_2]_2$[S1] (0.8 mg, 0.001 mmol, 0.025 equiv), and **9a** (17.1 mg, 0.0478 mmol, 1.0 equiv). Acetic acid (0.025 mL) was then added, and the vial was equipped with a stir bar. The reaction vial was sealed, and then outside the glove box, the vial was heated at 50 $^{\circ}$C in a preset oil bath for 20 h with stirring. The reaction mixture was then concentrated, and 10.4 mg (0.0618 mmol) of an external standard (1,3,5-trimethoxybenzene) was added to the reaction mixture. $CDCl_3$ was added to the reaction mixture, and the crude NMR was taken to determine that 44% of compound **3a** was formed along with 1% of recovered **9a**.

**Table 4, entry 4:**

In a $N_2$-filled glove box, a 2-5 mL microwave vial was charged with **9a** (17.2 mg, 0.0481 mmol, 1.0 equiv). Acetic acid (0.025 mL) was then added, and the vial was equipped with a stir bar. The reaction vial was sealed, and then outside the glove box, the vial was heated at 50 °C in a preset oil bath for 20 h with stirring. The reaction mixture was then concentrated, and 14.1 mg (0.0838 mmol) of an external standard (1,3,5-trimethoxybenzene) was added to the reaction mixture. $CDCl_3$ was added to the reaction mixture, and the crude NMR was taken to determine that 45% of compound **3a** was formed with no observed amount of recovered **9a**.

Table 4, entry 1

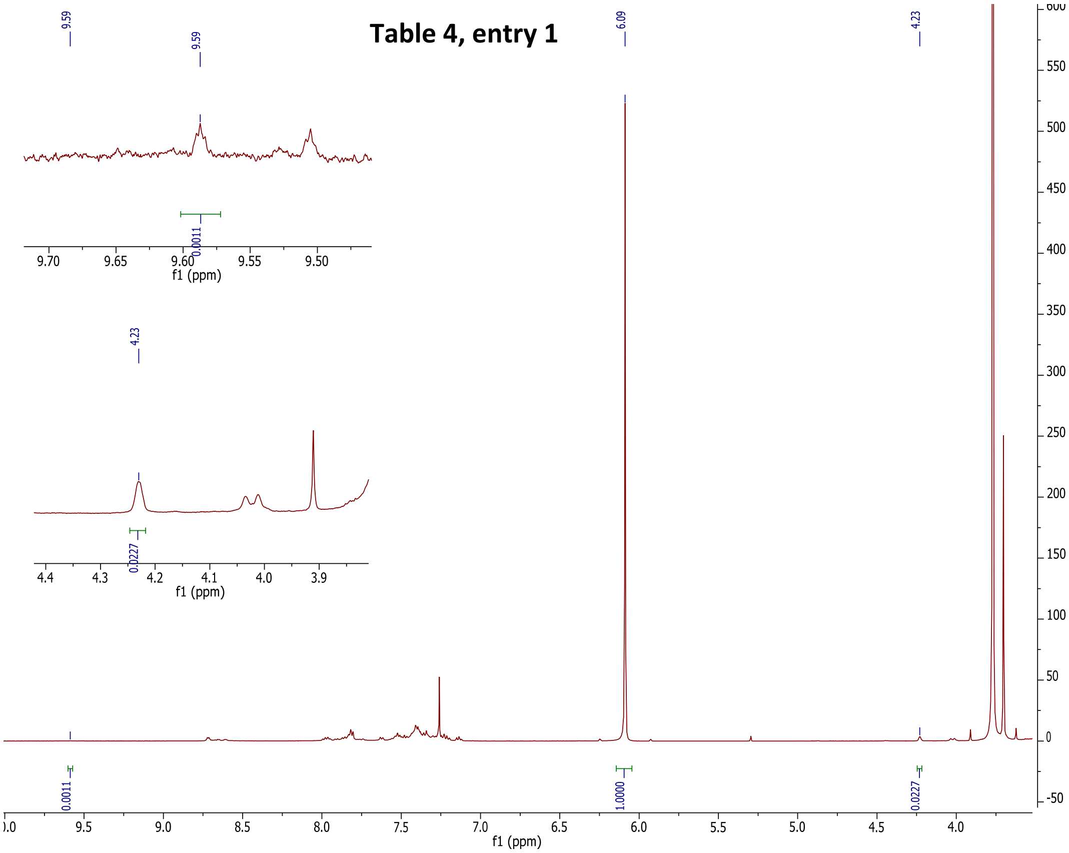


Table 4, entry 2

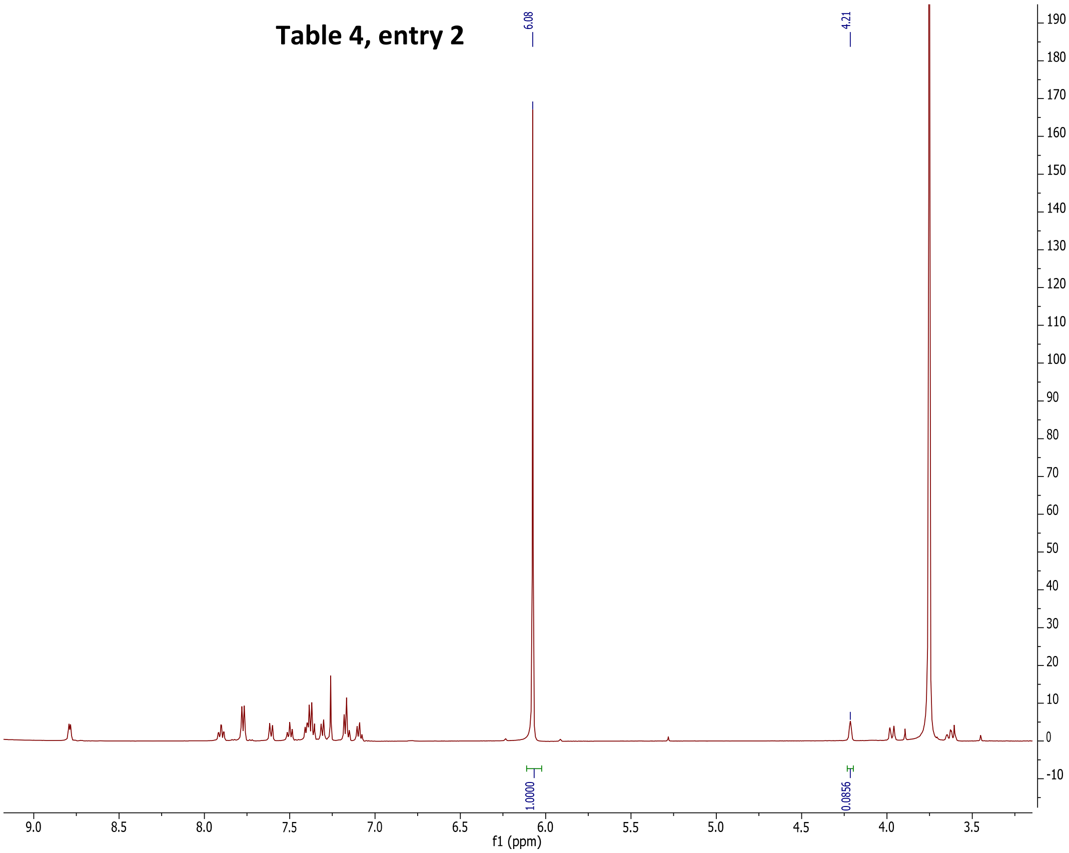

Table 4, entry 3

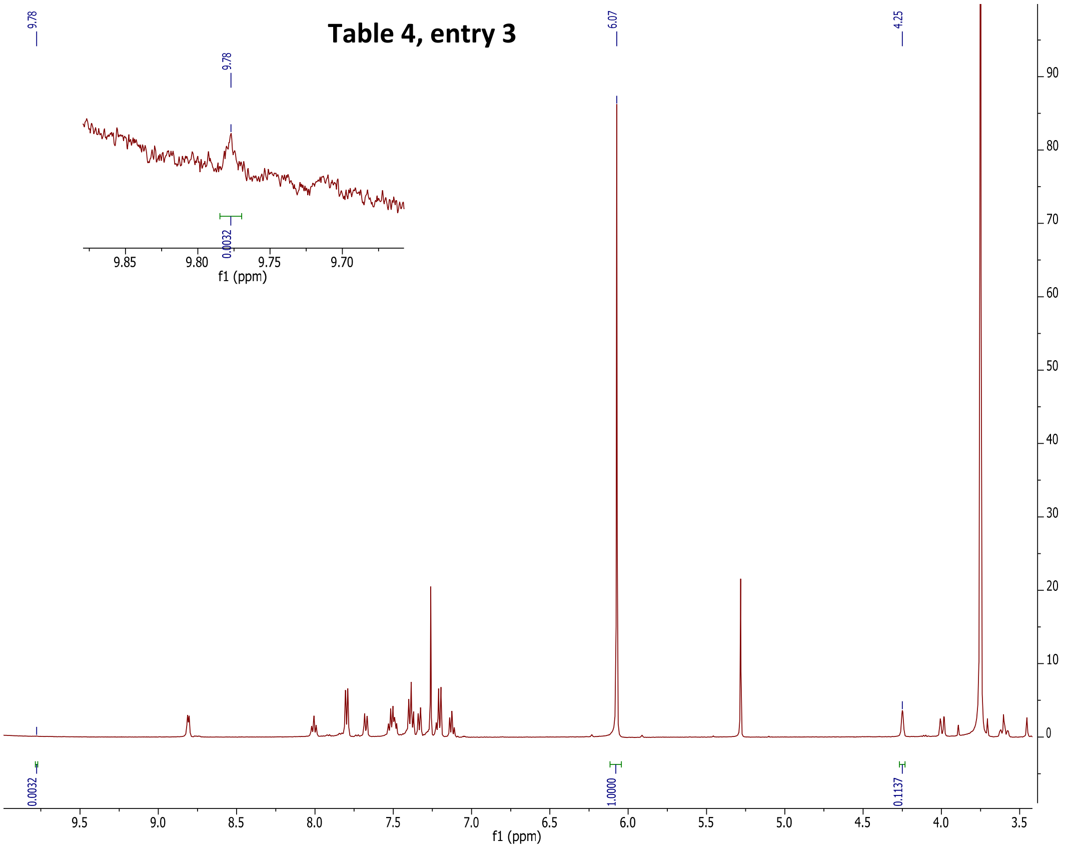


Table 4, entry 4

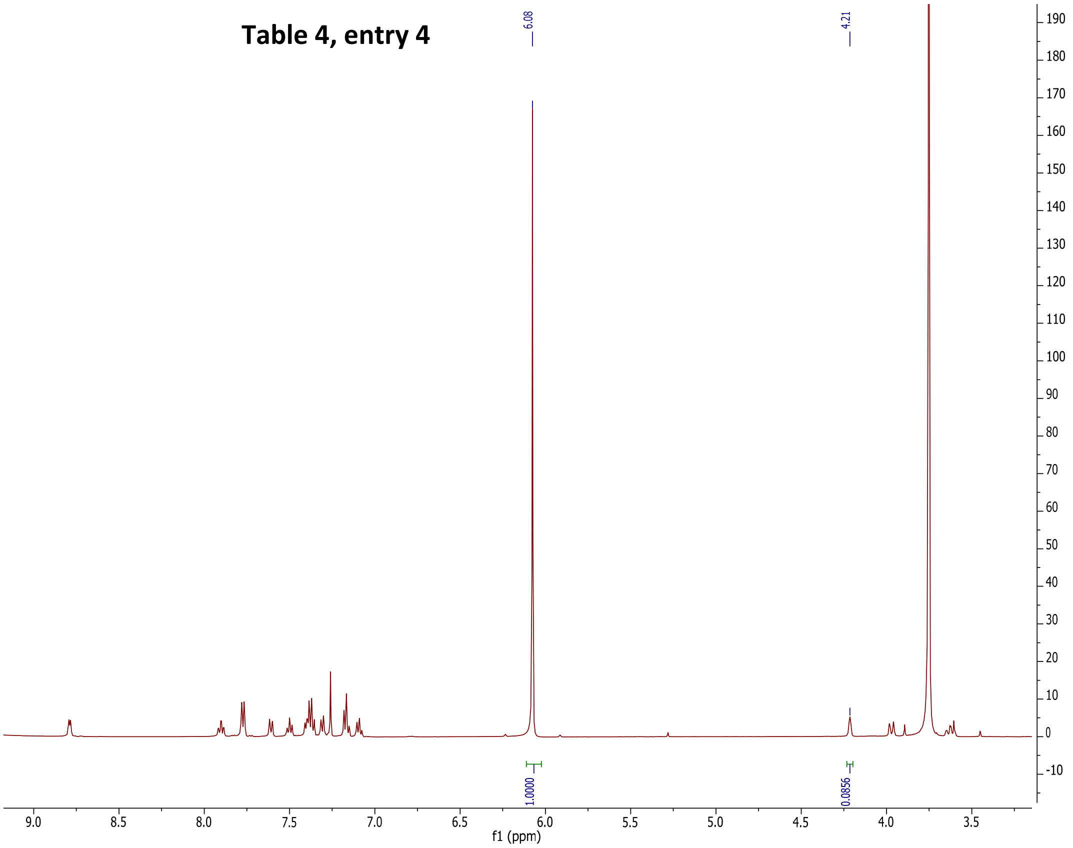

## V. Procedure for Rhodacycle Synthesis:

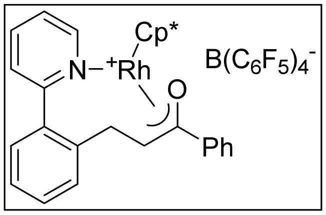


**Synthesis of Rh(III)-Enolate 10:**

In a $N_2$-filled glove box, a 2-5 mL microwave vial was charged with $AgB(C_6F_5)_4(Et_2O)_2$[S2] (187.0 mg, 0.2000 mmol, 4.000 equiv), $[Cp*RhCl_2]_2$[S1] (30.9 mg, 0.0500 mmol, 1.00 equiv), 2-phenylpyridine (17.1 mg, 0.110 mmol, 2.20 equiv), and phenyl vinyl ketone[S15] (14.5 mg, 0.110 mmol, 2.20 equiv). 1,2-dichloroethane (0.5 mL) was then added, and the vial was equipped with a stir bar. The reaction vial was sealed and then outside the glove box, the vial was stirred at room temperature for 30 min. The reaction mixture was then filtered over a plug of celite (1 cm celite in a glass pipette) eluting with $CH_2Cl_2$ and concentrated. Chromatography eluting with a 4:1 solution of dichloromethane/hexanes provided the product **10** (58.0 mg, 96% yield) as a reddish-orange solid (mp: 104-106 $^{\circ}$C). IR (film): 1644, 1513, 1459, 1083, 977, 756, 683, 655 $cm^{-1}$; $^{1}$H NMR (500 MHz, $CDCl_3$) δ 9.23 (d, *J* = 5.2 Hz, 1H), 8.06 (dt, *J* = 7.7, 1.4 Hz, 1H), 8.02 (d, *J* = 7.4 Hz, 2H), 7.74 (d, *J* = 7.8 Hz, 1H), 7.67 (t, *J* = 7.4 Hz, 1H), 7.60-7.47 (m, 7H), 4.89 (dd, *J* = 11.8, 5.3 Hz, 1H), 3.07 (dd, *J* = 13.5, 5.3 Hz, 1H), 2.22-2.17 (m, 1H), 0.99 (s, 15H). $^{13}C\{^{1}H\}$ NMR ($CDCl_3$), 126 MHz): δ 161.22, 153.80, 153.79, 149.42-149.18 (m), 147.49-147.27 (m), 139.90, 139.06, 137.51-137.21 (m), 135.99, 135.57-135.24 (m), 134.69, 132.78, 131.16, 130.14, 130.11, 129.20, 128.82, 128.60, 127.75, 124.16, 98.95, 65.51, 34.54, 8.36.; HRMS (ESI) calcd. for $C_{30}H_{31}NORh^{+}$: 524.1455 Found 524.1457.

# VI. X-Ray Crystallographic Data:

Single crystals of **3b** were obtained by slow diffusion of hexanes into a concentrated solution of product **3b** in toluene.

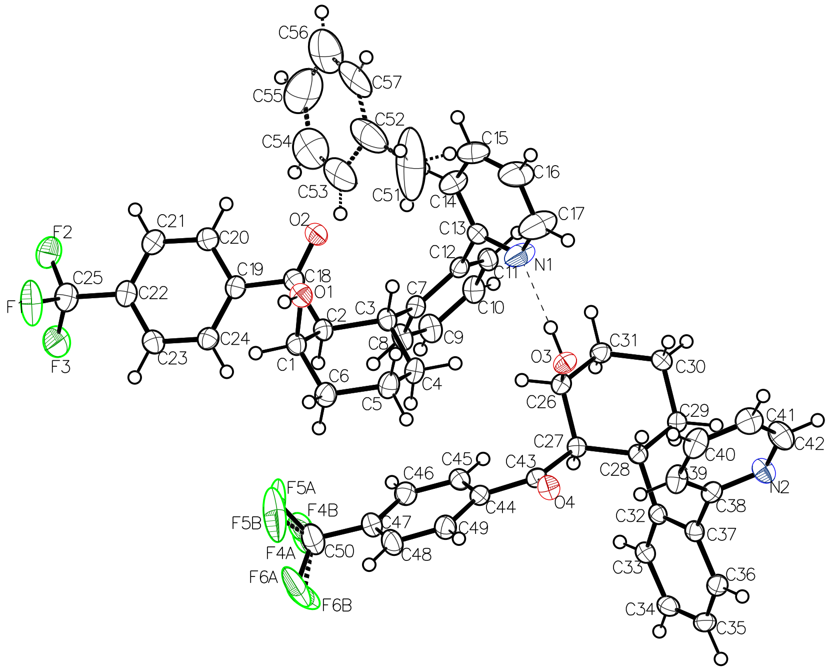


**Figure S1**. A thermal ellipsoid plot of **3b** at a 50% probability level. The full numbering scheme is shown. All hydrogen atoms are depicted as spheres. Carbon atoms C1 has R chirality; C2 has S; C3 has R; C26 has S; C27 has R; C28 has S.

**Table S1**. Crystal data and structure refinement for **3b**.

| | | |
|---|---|---|
| Identification code | 007-15013 | |
| Empirical formula | $C_{53.50}H_{48}F_6N_2O_4$ | |
| Formula weight | 896.94 | |
| Temperature | 93(2) K | |
| Wavelength | 1.54187 Å | |
| Crystal system | Triclinic | |
| Space group | $P\bar{1}$ | |
| Unit cell dimensions | a = 9.6325(2) Å | α = 96.651(7)°. |
| | b = 13.4130(3) Å | β = 91.573(7)°. |
| | c = 18.8693(13) Å | γ = 109.521(8)°. |
| Volume | 2276.6(2) $Å^3$ | |
| Z | 2 | |
| Density (calculated) | 1.308 $Mg/m^3$ | |
| Absorption coefficient | 0.825 $mm^{-1}$ | |
| F(000) | 938 | |
| Crystal size | 0.200 x 0.200 x 0.040 $mm^3$ | |
| Crystal color and habit | Colorless Prism | |
| Diffractometer | Rigaku Saturn 944+ CCD | |
| Θ range for data collection | 2.363 to 66.591°. | |
| Index ranges | $-11 \le h \le 11$, $-15 \le k \le 15$, $-22 \le l \le 22$ | |
| Reflections collected | 59268 | |
| Independent reflections | 7910 [R(int) = 0.0480] | |
| Observed reflections (I > 2σ(I)) | 7081 | |
| Completeness to θ = 66.591° | 98.3 % | |

| | |
|---|---|
| Absorption correction | Semi-empirical from equivalents |
| Max. and min. transmission | 0.968 and 0.802 |
| Solution method | SHELXS-2013 (Sheldrick, 2013) |
| Refinement method | SHELXL-2014/7 (Sheldrick, 2014) |
| Data / restraints / parameters | 7910 / 51 / 659 |
| Goodness-of-fit on $F^2$ | 1.072 |
| Final R indices [$I>2\sigma(I)$] | R1 = 0.0394, wR2 = 0.1018 |
| R indices (all data) | R1 = 0.0428, wR2 = 0.1039 |
| Largest diff. peak and hole | 0.529 and -0.338 e.$Å^{-3}$ |

**Table S2**. Atomic coordinates ( x $10^4$) and equivalent isotropic displacement parameters ($Å^2$x $10^3$) for 007-15013. U(eq) is defined as one third of the trace of the orthogonalized $U^{ij}$ tensor.

| | x | y | z | U(eq) |
|---|---|---|---|---|
| F(1) | 3262(2) | 10021(1) | 9526(1) | 63(1) |
| F(2) | 1167(1) | 9285(1) | 9938(1) | 51(1) |
| F(3) | 3126(1) | 9208(1) | 10442(1) | 57(1) |
| O(1) | 3633(1) | 5870(1) | 6631(1) | 31(1) |
| O(2) | 736(1) | 4918(1) | 7512(1) | 33(1) |
| N(1) | 908(2) | 1503(2) | 6125(1) | 54(1) |
| C(1) | 4388(2) | 5836(1) | 7280(1) | 26(1) |
| C(2) | 3257(2) | 5038(1) | 7691(1) | 24(1) |
| C(3) | 2784(2) | 3894(1) | 7290(1) | 24(1) |
| C(4) | 4155(2) | 3576(1) | 7162(1) | 29(1) |
| C(5) | 5273(2) | 4354(1) | 6753(1) | 32(1) |
| C(6) | 5723(2) | 5487(1) | 7147(1) | 31(1) |
| C(7) | 1704(2) | 3070(1) | 7679(1) | 24(1) |
| C(8) | 1893(2) | 3097(1) | 8418(1) | 31(1) |
| C(9) | 976(2) | 2317(1) | 8776(1) | 34(1) |
| C(10) | -154(2) | 1476(1) | 8402(1) | 32(1) |
| C(11) | -363(2) | 1428(1) | 7668(1) | 27(1) |
| C(12) | 551(2) | 2215(1) | 7303(1) | 23(1) |
| C(13) | 276(2) | 2105(1) | 6510(1) | 25(1) |
| C(14) | -626(2) | 2562(1) | 6194(1) | 37(1) |

| | | | | |
|---|---|---|---|---|
| C(15) | -889(2) | 2394(2) | 5459(1) | 44(1) |
| C(16) | -231(2) | 1792(2) | 5064(1) | 57(1) |
| C(17) | 652(3) | 1365(2) | 5414(1) | 80(1) |
| C(18) | 1932(2) | 5388(1) | 7833(1) | 25(1) |
| C(19) | 2096(2) | 6343(1) | 8379(1) | 24(1) |
| C(20) | 899(2) | 6708(1) | 8439(1) | 27(1) |
| C(21) | 988(2) | 7597(1) | 8914(1) | 30(1) |
| C(22) | 2290(2) | 8145(1) | 9329(1) | 29(1) |
| C(23) | 3485(2) | 7786(1) | 9285(1) | 29(1) |
| C(24) | 3382(2) | 6885(1) | 8816(1) | 26(1) |
| C(25) | 2449(2) | 9154(1) | 9807(1) | 36(1) |
| F(4A) | 6589(12) | 4971(8) | 10219(5) | 55(2) |
| F(4B) | 6474(10) | 5019(9) | 10180(6) | 52(2) |
| F(5A) | 6900(20) | 5826(10) | 9325(5) | 67(2) |
| F(5B) | 7300(30) | 5917(13) | 9337(8) | 99(5) |
| F(6A) | 8710(13) | 5529(10) | 9766(9) | 80(3) |
| F(6B) | 8672(13) | 5322(18) | 9965(13) | 87(4) |
| O(3) | 3063(1) | 730(1) | 6629(1) | 27(1) |
| O(4) | 6408(1) | 1326(1) | 7031(1) | 29(1) |
| N(2) | 5224(1) | -2531(1) | 5777(1) | 32(1) |
| C(26) | 2909(2) | 535(1) | 7352(1) | 23(1) |
| C(27) | 4315(1) | 325(1) | 7604(1) | 21(1) |
| C(28) | 4412(1) | -690(1) | 7168(1) | 21(1) |
| C(29) | 3019(2) | -1641(1) | 7244(1) | 23(1) |
| C(30) | 1618(2) | -1444(1) | 7014(1) | 26(1) |

| | | | | |
|---|---|---|---|---|
| C(31) | 1538(2) | -419(1) | 7425(1) | 25(1) |
| C(32) | 5746(1) | -975(1) | 7376(1) | 21(1) |
| C(33) | 6195(2) | -920(1) | 8094(1) | 24(1) |
| C(34) | 7304(2) | -1297(1) | 8292(1) | 28(1) |
| C(35) | 8000(2) | -1732(1) | 7775(1) | 28(1) |
| C(36) | 7586(2) | -1783(1) | 7059(1) | 26(1) |
| C(37) | 6465(2) | -1414(1) | 6854(1) | 22(1) |
| C(38) | 6016(2) | -1537(1) | 6076(1) | 24(1) |
| C(39) | 6406(2) | -680(1) | 5686(1) | 33(1) |
| C(40) | 5977(2) | -854(1) | 4963(1) | 38(1) |
| C(41) | 5159(2) | -1872(1) | 4652(1) | 37(1) |
| C(42) | 4808(2) | -2677(1) | 5076(1) | 39(1) |
| C(43) | 5668(2) | 1291(1) | 7545(1) | 23(1) |
| C(44) | 6082(2) | 2235(1) | 8124(1) | 24(1) |
| C(45) | 5197(2) | 2319(1) | 8684(1) | 25(1) |
| C(46) | 5590(2) | 3237(1) | 9175(1) | 29(1) |
| C(47) | 6887(2) | 4067(1) | 9118(1) | 32(1) |
| C(48) | 7803(2) | 3980(1) | 8575(1) | 36(1) |
| C(49) | 7394(2) | 3073(1) | 8080(1) | 31(1) |
| C(50) | 7304(2) | 5078(1) | 9627(1) | 45(1) |
| C(51) | 1421(12) | 4315(9) | 4219(6) | 154(5) |
| C(52) | 422(5) | 4809(4) | 4737(3) | 85(2) |
| C(53) | 1078(8) | 5216(7) | 5421(3) | 68(2) |
| C(54) | 117(5) | 5508(4) | 5869(3) | 78(1) |
| C(55) | -1234(8) | 5564(9) | 5630(4) | 100(3) |

| | | | | |
|---|---|---|---|---|
| C(56) | -1820(6) | 5304(4) | 4921(3) | 84(2) |
| C(57) | -822(7) | 5009(7) | 4498(4) | 67(2) |

Single crystals of **10** were obtained by slow evaporation of a concentrated solution of product **10** in ethyl ether.

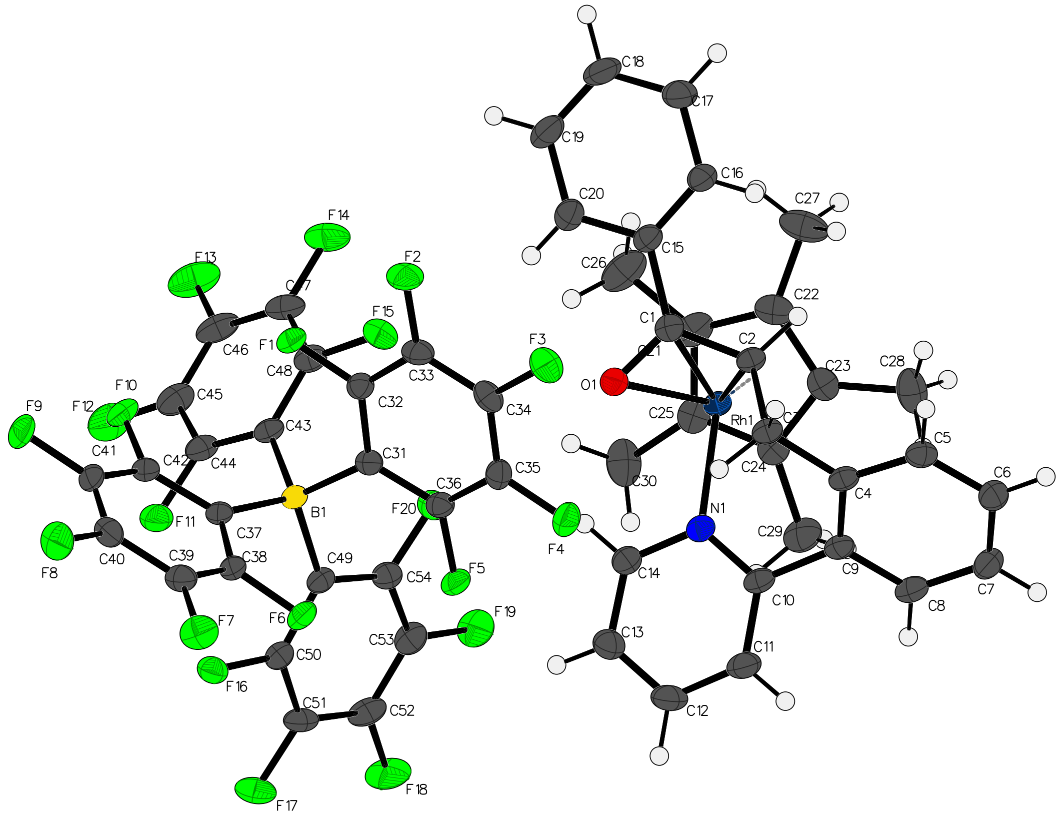


Figure S2. The full numbering scheme of **10** with 50% thermal ellipsoids. The hydrogen atoms are show as arbitrary circles for clarity.

Table S3. Crystal data and structure refinement for **10**.

| | | |
|---|---|---|
| Identification code | 007-15127 | |
| Empirical formula | $C_{54}H_{31}BF_{20}NORh$ | |
| Formula weight | 1203.52 | |
| Temperature | 93(2) K | |
| Wavelength | 1.54178 Å | |
| Crystal system | Triclinic | |
| Space group | P-1 | |
| Unit cell dimensions | a = 11.8540(8) Å | α = 79.702(2)°. |
| | b = 13.0781(9) Å | β = 80.128(2)°. |
| | c = 16.1540(11) Å | γ = 73.163(2)°. |
| Volume | 2339.2(3) $Å^3$ | |
| Z | 2 | |
| Density (calculated) | 1.709 $Mg/m^3$ | |
| Absorption coefficient | 4.074 $mm^{-1}$ | |
| F(000) | 1200 | |
| Crystal size | 0.080 x 0.080 x 0.040 $mm^3$ | |
| Theta range for data collection | 2.803 to 68.095°. | |
| Index ranges | $-14 \leq h \leq 14$, $-15 \leq k \leq 15$, $-19 \leq l \leq 19$ | |
| Reflections collected | 75602 | |
| Independent reflections | 8279 [R(int) = 0.0573] | |
| Completeness to theta = 67.679° | 97.7 % | |
| Absorption correction | Semi-empirical from equivalents | |
| Max. and min. transmission | 1.000 and 0.788 | |
| Refinement method | Full-matrix least-squares on $F^2$ | |

| | |
|---|---|
| Data / restraints / parameters | 8279 / 0 / 712 |
| Goodness-of-fit on $F^2$ | 1.126 |
| Final R indices [I>2sigma(I)] | R1 = 0.0250, wR2 = 0.0727 |
| R indices (all data) | R1 = 0.0260, wR2 = 0.0731 |
| Largest diff. peak and hole | 0.401 and -0.735 e.Å$^{-3}$ |

Table S4. Atomic coordinates ( x $10^4$) and equivalent isotropic displacement parameters ($Å^2$x $10^3$) for 007-15127. U(eq) is defined as one third of the trace of the orthogonalized $U^{ij}$ tensor.

| | x | y | z | U(eq) |
|---|---|---|---|---|
| Rh(1) | 573(1) | 8052(1) | 2161(1) | 18(1) |
| O(1) | 998(1) | 6541(1) | 2990(1) | 22(1) |
| N(1) | 2173(2) | 8191(1) | 2511(1) | 20(1) |
| C(1) | -95(2) | 7006(2) | 3263(1) | 20(1) |
| C(2) | -337(2) | 8096(2) | 3439(1) | 19(1) |
| C(3) | 350(2) | 8431(2) | 4006(1) | 20(1) |
| C(4) | 380(2) | 9591(2) | 3726(1) | 20(1) |
| C(5) | -526(2) | 10422(2) | 4048(1) | 24(1) |
| C(6) | -543(2) | 11500(2) | 3790(1) | 27(1) |
| C(7) | 375(2) | 11765(2) | 3220(1) | 28(1) |
| C(8) | 1293(2) | 10954(2) | 2900(1) | 27(1) |
| C(9) | 1299(2) | 9869(2) | 3137(1) | 21(1) |
| C(10) | 2336(2) | 9038(2) | 2809(1) | 21(1) |
| C(11) | 3475(2) | 9125(2) | 2822(1) | 28(1) |
| C(12) | 4455(2) | 8353(2) | 2532(2) | 32(1) |
| C(13) | 4286(2) | 7478(2) | 2250(1) | 29(1) |
| C(14) | 3138(2) | 7419(2) | 2260(1) | 24(1) |
| C(15) | -1021(2) | 6439(2) | 3283(1) | 22(1) |
| C(16) | -2194(2) | 6851(2) | 3627(1) | 26(1) |
| C(17) | -3038(2) | 6292(2) | 3648(2) | 33(1) |

| | | | | |
|---|---|---|---|---|
| C(18) | -2704(2) | 5306(2) | 3342(2) | 36(1) |
| C(19) | -1533(2) | 4877(2) | 3025(2) | 33(1) |
| C(20) | -689(2) | 5433(2) | 2989(1) | 26(1) |
| C(21) | -43(2) | 7720(2) | 1052(1) | 30(1) |
| C(22) | -918(2) | 8583(2) | 1430(1) | 28(1) |
| C(23) | -381(2) | 9444(2) | 1392(1) | 25(1) |
| C(24) | 810(2) | 9111(2) | 977(1) | 24(1) |
| C(25) | 1027(2) | 8047(2) | 772(1) | 27(1) |
| C(26) | -211(3) | 6669(2) | 929(2) | 44(1) |
| C(27) | -2186(2) | 8620(2) | 1753(2) | 41(1) |
| C(28) | -989(2) | 10521(2) | 1669(2) | 38(1) |
| C(29) | 1662(2) | 9796(2) | 771(1) | 34(1) |
| C(30) | 2167(2) | 7408(2) | 326(2) | 40(1) |
| F(1) | 2851(1) | 2278(1) | 3557(1) | 24(1) |
| F(2) | 1125(1) | 3289(1) | 4648(1) | 31(1) |
| F(3) | 1106(1) | 5281(1) | 4987(1) | 33(1) |
| F(4) | 2896(1) | 6175(1) | 4241(1) | 29(1) |
| F(5) | 4591(1) | 5203(1) | 3128(1) | 24(1) |
| F(6) | 6071(1) | 3257(1) | 3903(1) | 24(1) |
| F(7) | 6920(1) | 1799(1) | 5177(1) | 32(1) |
| F(8) | 6864(1) | -290(1) | 5282(1) | 37(1) |
| F(9) | 5924(1) | -874(1) | 4062(1) | 34(1) |
| F(10) | 5067(1) | 548(1) | 2804(1) | 28(1) |
| F(11) | 6221(1) | 1566(1) | 1432(1) | 29(1) |
| F(12) | 5538(1) | 887(1) | 189(1) | 44(1) |

| | | | | |
|---|---|---|---|---|
| F(13) | 3205(1) | 1523(1) | -84(1) | 47(1) |
| F(14) | 1595(1) | 2863(1) | 925(1) | 37(1) |
| F(15) | 2254(1) | 3630(1) | 2106(1) | 29(1) |
| F(16) | 7547(1) | 2593(1) | 2396(1) | 25(1) |
| F(17) | 8793(1) | 3726(1) | 1290(1) | 34(1) |
| F(18) | 7701(1) | 5503(1) | 274(1) | 42(1) |
| F(19) | 5276(1) | 6098(1) | 376(1) | 38(1) |
| F(20) | 3996(1) | 4962(1) | 1473(1) | 28(1) |
| C(31) | 3779(2) | 3707(2) | 3249(1) | 19(1) |
| C(32) | 2878(2) | 3266(2) | 3692(1) | 20(1) |
| C(33) | 1980(2) | 3767(2) | 4267(1) | 24(1) |
| C(34) | 1973(2) | 4761(2) | 4446(1) | 24(1) |
| C(35) | 2874(2) | 5215(2) | 4057(1) | 22(1) |
| C(36) | 3741(2) | 4692(2) | 3474(1) | 20(1) |
| C(37) | 5512(2) | 2003(2) | 3271(1) | 19(1) |
| C(38) | 6012(2) | 2236(2) | 3915(1) | 20(1) |
| C(39) | 6456(2) | 1504(2) | 4580(1) | 24(1) |
| C(40) | 6434(2) | 442(2) | 4636(1) | 27(1) |
| C(41) | 5966(2) | 157(2) | 4022(1) | 25(1) |
| C(42) | 5526(2) | 926(2) | 3362(1) | 21(1) |
| C(43) | 4278(2) | 2598(2) | 1874(1) | 20(1) |
| C(44) | 5047(2) | 1917(2) | 1330(1) | 25(1) |
| C(45) | 4721(2) | 1548(2) | 685(1) | 31(1) |
| C(46) | 3555(2) | 1863(2) | 545(1) | 33(1) |
| C(47) | 2746(2) | 2545(2) | 1049(1) | 28(1) |

| | | | | |
|---|---|---|---|---|
| C(48) | 3118(2) | 2914(2) | 1681(1) | 24(1) |
| C(49) | 5691(2) | 3717(2) | 2009(1) | 19(1) |
| C(50) | 6920(2) | 3464(2) | 1924(1) | 21(1) |
| C(51) | 7597(2) | 4045(2) | 1354(1) | 25(1) |
| C(52) | 7052(2) | 4940(2) | 834(1) | 29(1) |
| C(53) | 5830(2) | 5231(2) | 887(1) | 26(1) |
| C(54) | 5194(2) | 4624(2) | 1459(1) | 22(1) |
| B(1) | 4822(2) | 3007(2) | 2599(1) | 19(1) |

## VII. References:


S1. K.-I. Fujita, Y. Takahashi, M. Owaki, K. Yamamoto and R. Yamaguchi, *Org. Lett*., 2004, **6**, 2785.

S2. M. Kuprat, M. Lehmann, A. Schulz and A. Villinger, *Organometallics*, 2010, **29**, 1421.

S3. H. T. You, A. C. Grosse, J. K. Howard, C. J. T. Hyland, J. Just, P. P. Molesworth and J. A. Smith, *Org. Biomol. Chem.,* 2011, **9**, 3948.

S4. E. L. Richards, P. J. Murphy, F. Dinon, S. Fratucello, P. M. Brown, T. Gelbrich and M. B. Hursthouse, *Tetrahedron,* 2001, **57**, 7771.

S5. X. Zhang, P. Ma, D. Zhang, Y. Lei, S. Zhang, R. Jiang and W. Chen, *Org. Biomol. Chem*., 2014, **12**, 2423.

S6. M. Neumann and K. Zeitler, *Chem. Eur. J.,* 2013, **19**, 6950.

S7. C. E. Aroyan, M. M. Vasbinder and S. J. Miller, *Org. Lett*., 2005, **7**, 3849.

S8. E. J. Enholm and K. S. Kinter, *J. Org. Chem.,* 1995, **60**, 4850.

S9. E. Sánchez-Larios, J. M. Holmes, C. L. Daschner and M. Gravel, *Org. Lett.,* 2010, **12**, 5772.

S10. C. Liu and W.-B. Yang, *Chem. Commun*., 2009, 6267.

S11. D. M. Kang, J.-W. Kang, J. W. Park, S. O. Jung, S.-H. Lee, H.-D. Park, Y.-H. Kim, S. C. Shin, J.-J. Kim and S.-K. Kwon, *Adv. Mater*., 2008, **20**, 2003.

S12. L. Ackermann and A. V. Lygin, *Org. Lett.,* 2011, **13**, 3332.

S13. Y. Fukui, P. Liu, Q. Liu, Z. He, N. Wu, P. Tian and G. Lin, *J. Am. Chem. Soc.,* 2014, **136**, 15607.

S14. H. J. P. de Lijser and C. K. Tsai, *J. Org. Chem*., 2004, **69**, 3057.

S15. S. Chanthamath, S. Takaki, K. Shibatomi and S. Iwasa, *Angew. Chem. Int. Ed.,* 2013, **52**, 5818.

S16. K. D. Hesp, R. G. Bergman and J. A. Ellman, *Org. Lett*. 2012, **14**, 2304.

S17. L. O. Davis and S. L. Tobey, *Tetrahedron Lett*. 2010, **51**, 6078.

# VIII: NMR Data

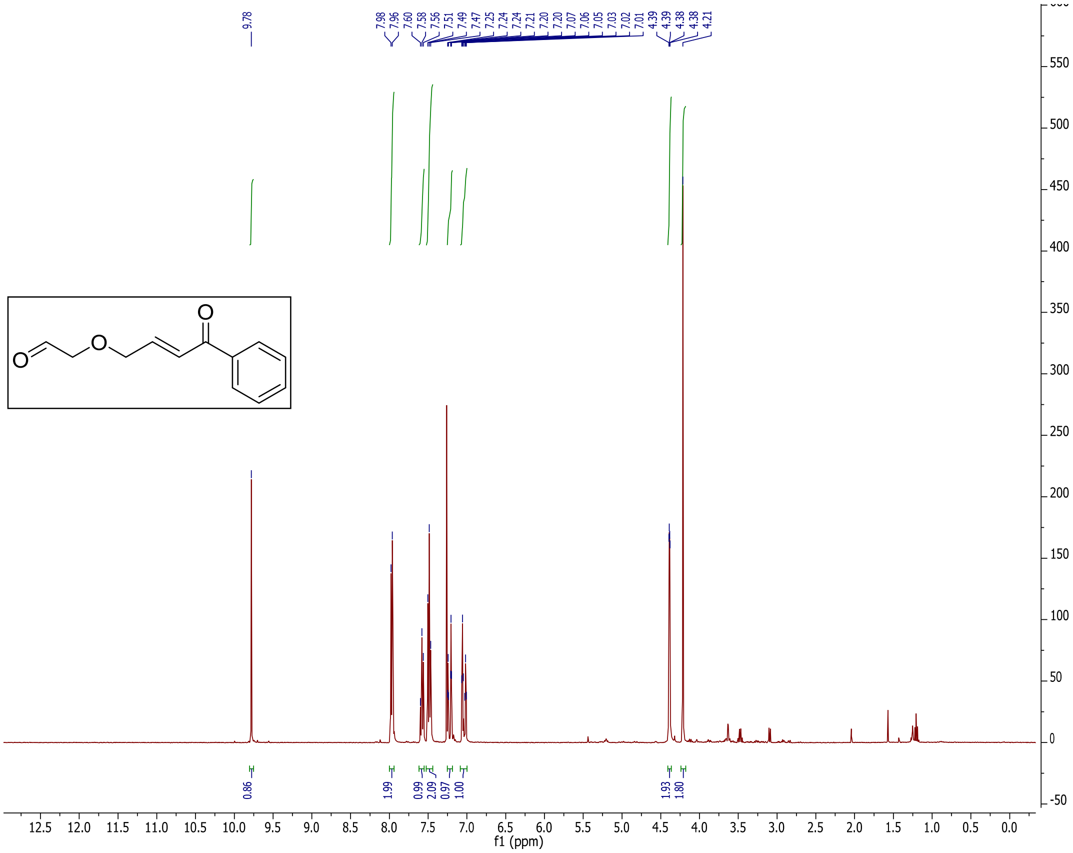

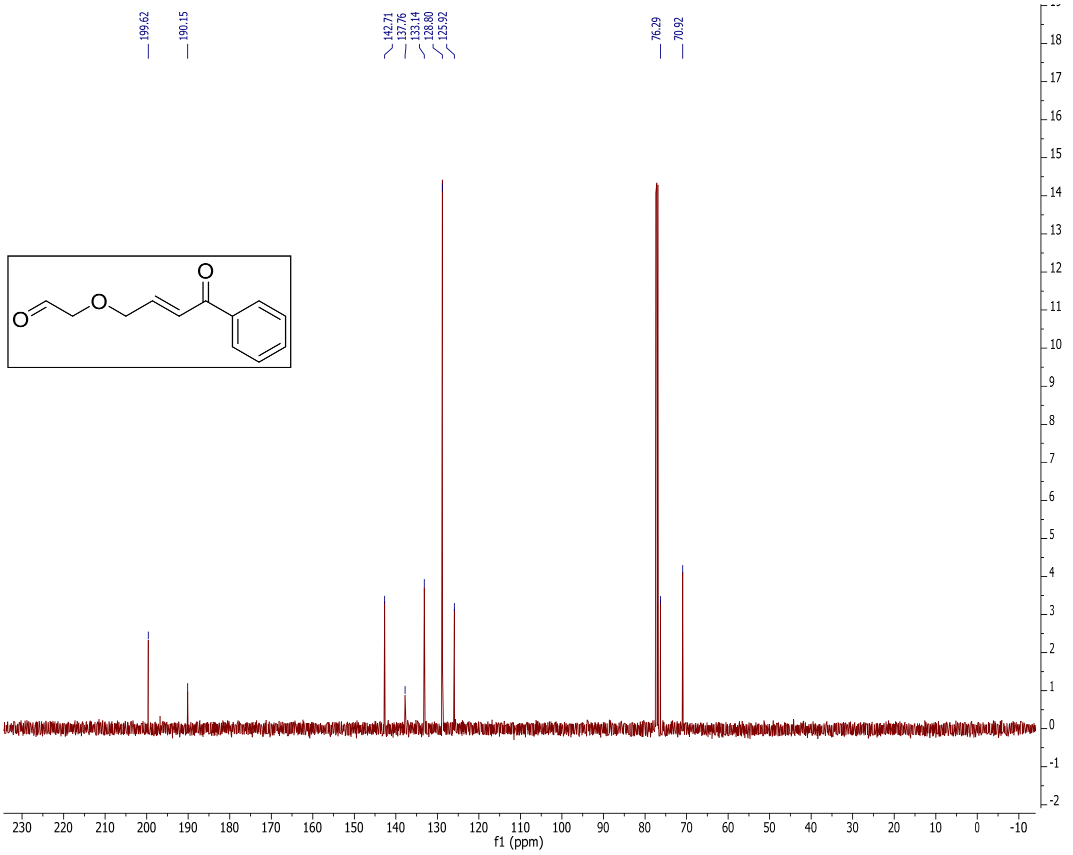

Crude $^{1}$H NMR of **2g** (500 MHz, $CDCl_3$):

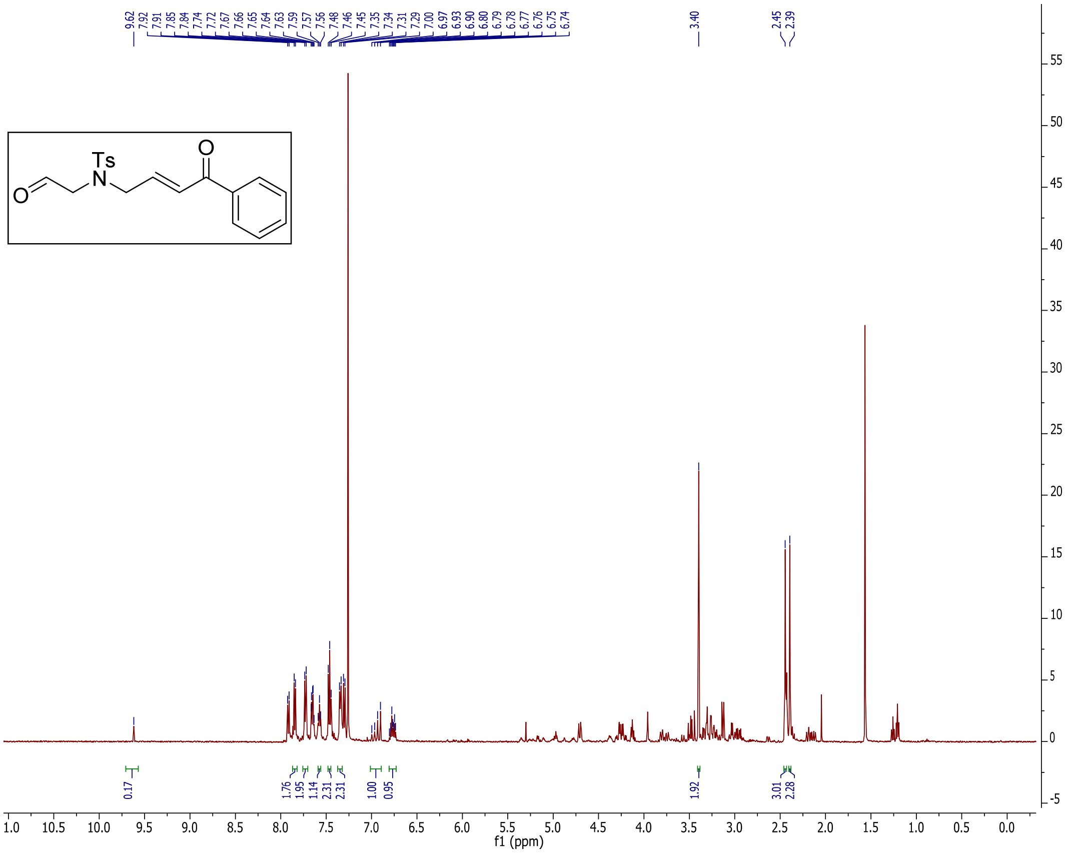

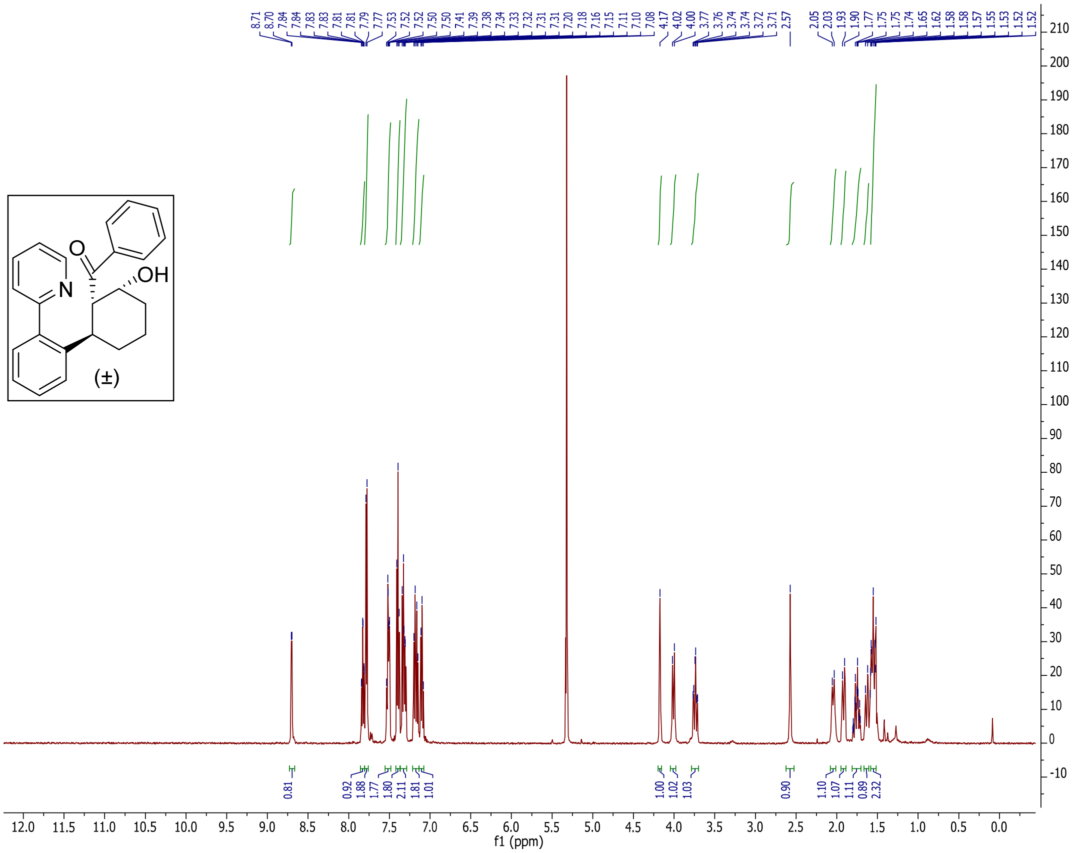

O
N
OH
(±)
8.71 8.70 7.84 7.83 7.83 7.81 7.81 7.79 7.77 7.53 7.52 7.52 7.50 7.50 7.41 7.39 7.38 7.34 7.33 7.32 7.31 7.20 7.18 7.16 7.15 7.11 7.10 7.08 4.17 4.02 4.00 3.77 3.76 3.74 3.74 3.72 3.71 2.57 2.05 2.03 1.93 1.90 1.77 1.75 1.75 1.74 1.65 1.62 1.58 1.58 1.57 1.55 1.53 1.52 1.52
0.81 0.92 1.88 1.77 1.80 2.11 1.81 1.01 1.00 1.02 1.03 0.90 1.10 1.07 1.11 0.89 2.32
f1 (ppm)


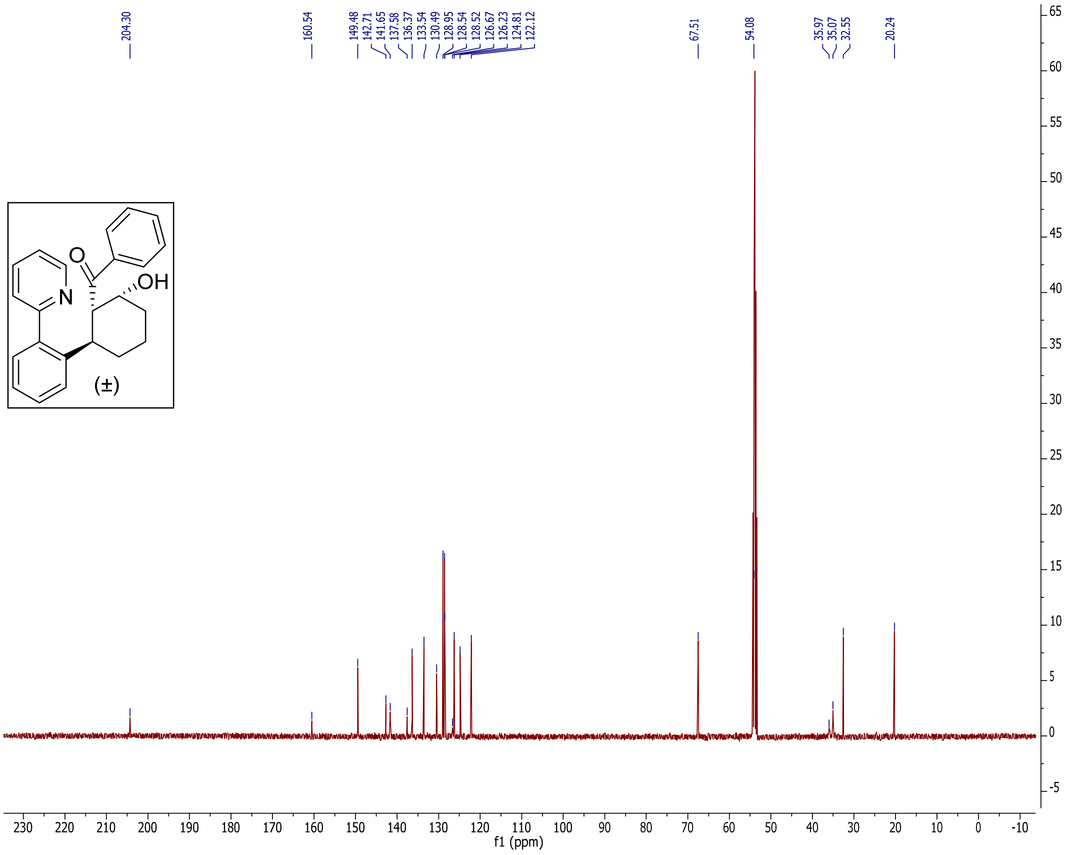

O
N
OH
(±)
204.30 160.54 149.48 142.71 141.65 137.58 136.37 133.54 130.49 128.95 128.54 128.52 126.67 126.23 124.81 122.12 67.51 54.08 35.97 35.07 32.55 20.24
f1 (ppm)

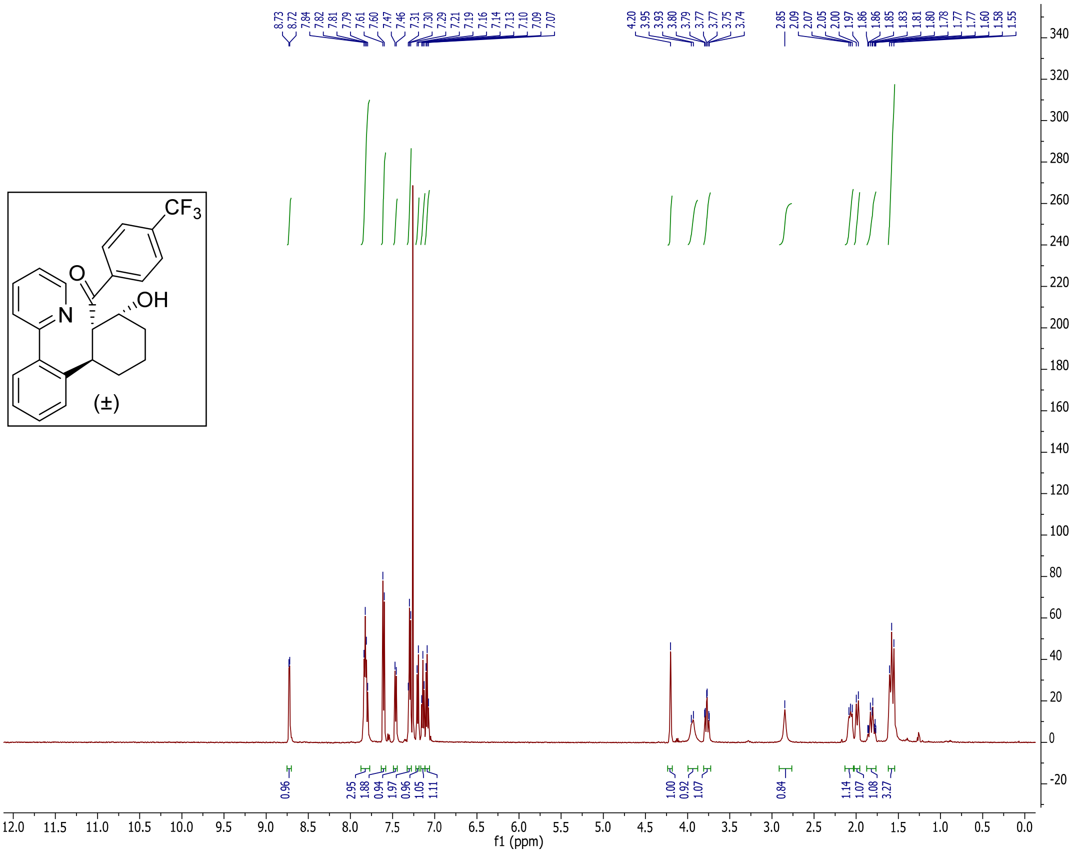

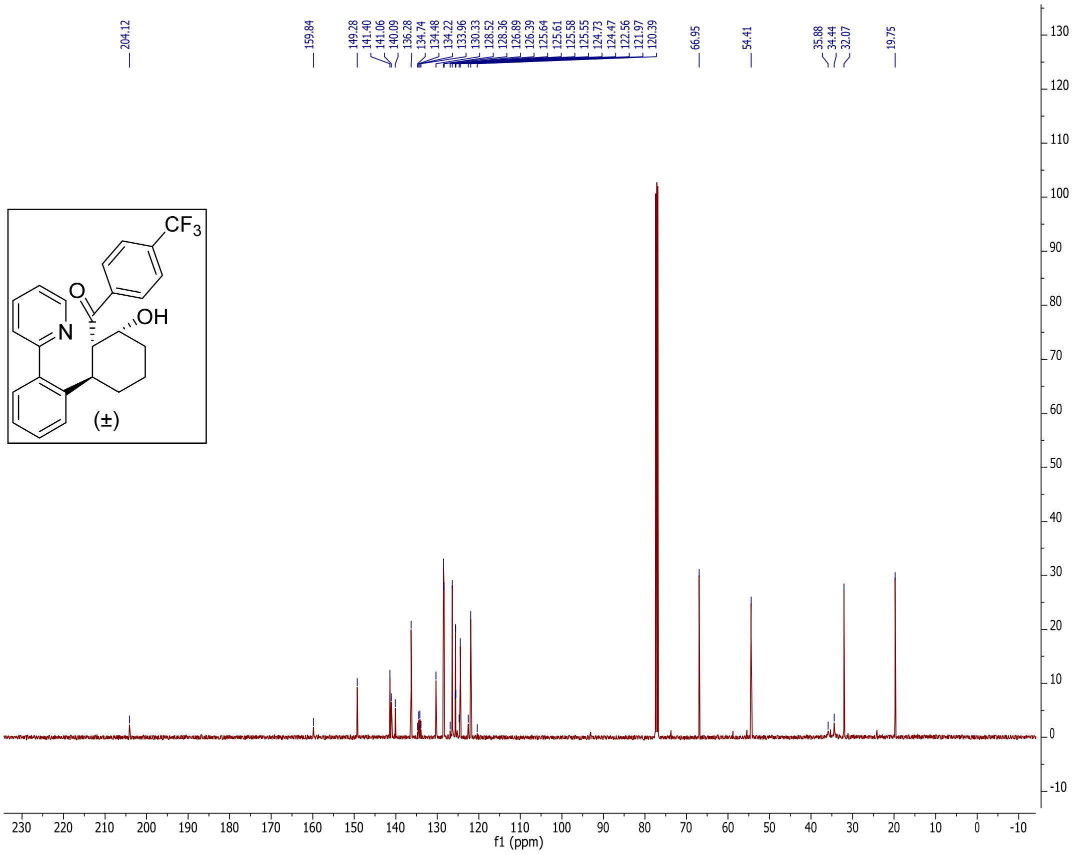

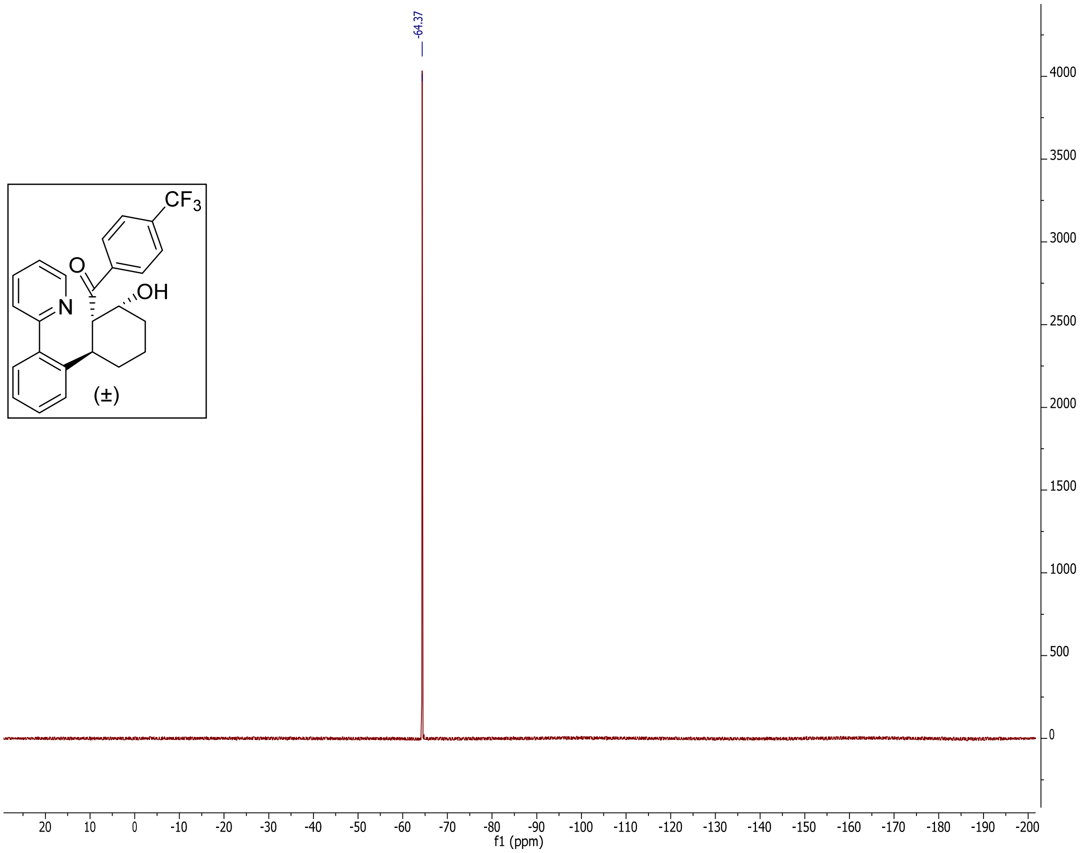

-64.37
CF3
O
N
OH
(±)
4000
3500
3000
2500
2000
1500
1000
500
0
20
10
0
-10
-20
-30
-40
-50
-60
-70
-80
-90
-100
-110
-120
-130
-140
-150
-160
-170
-180
-190
-200
f1 (ppm)

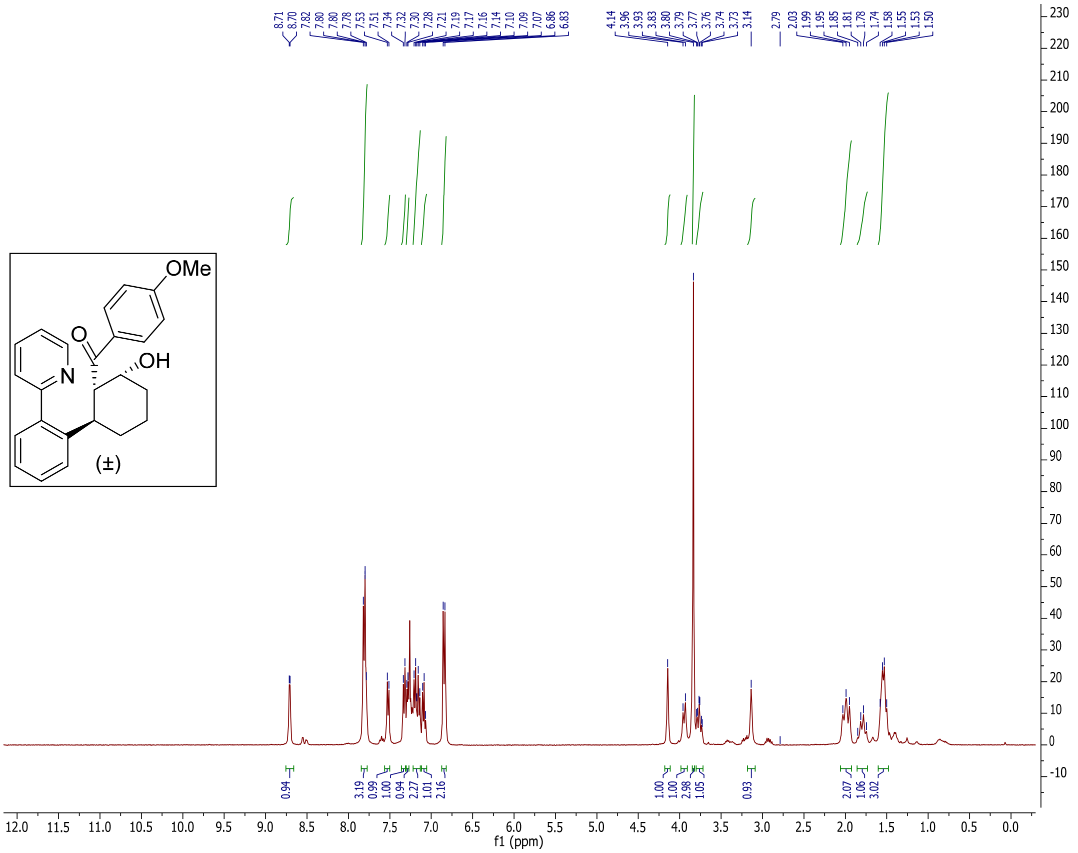

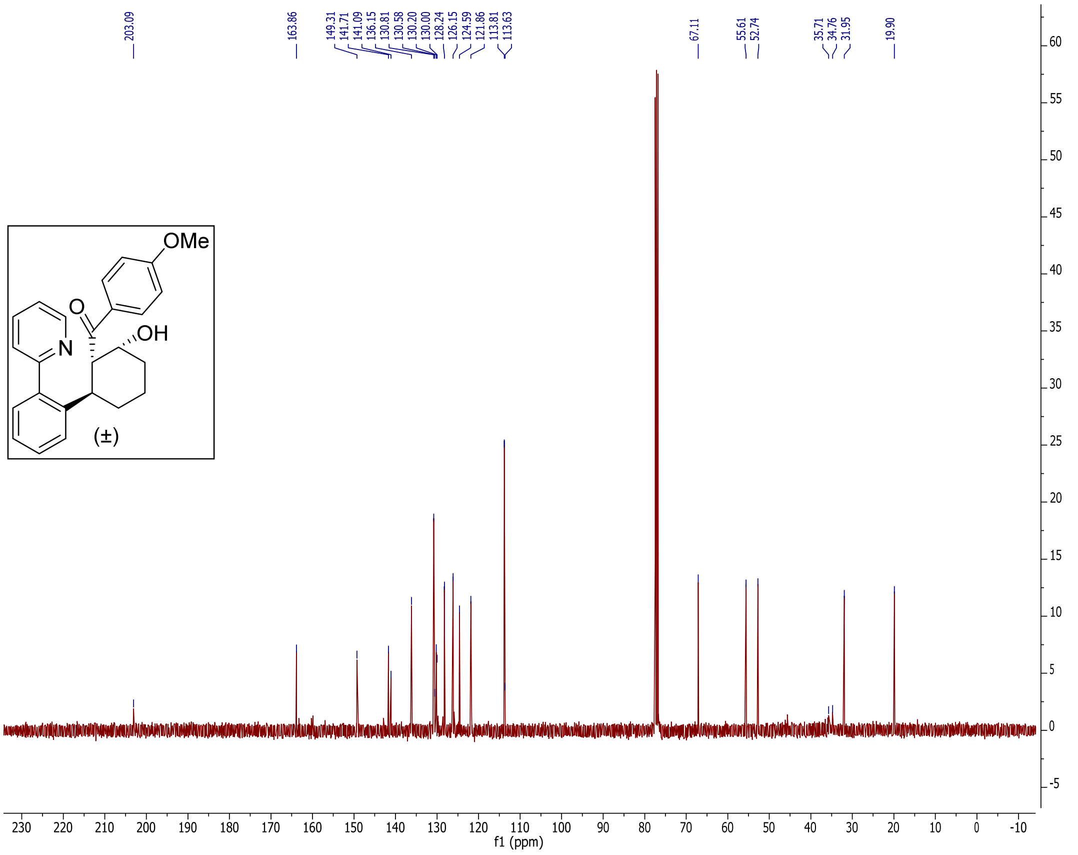

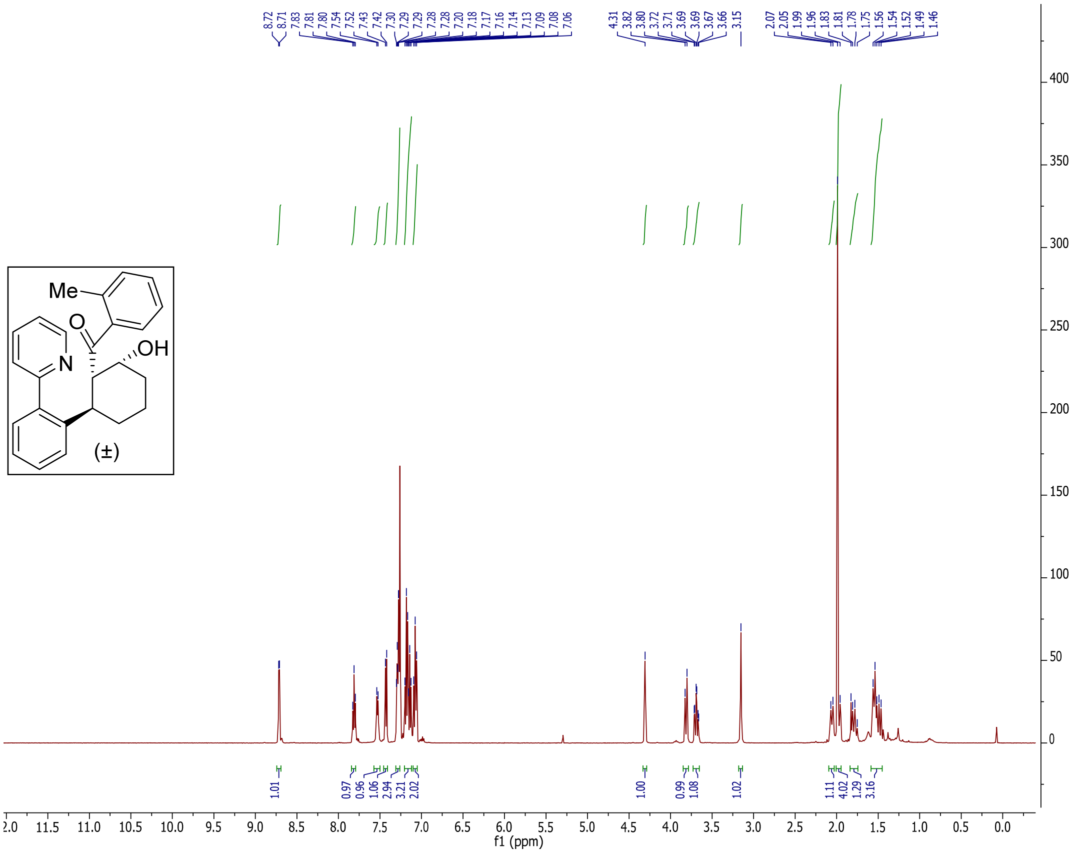

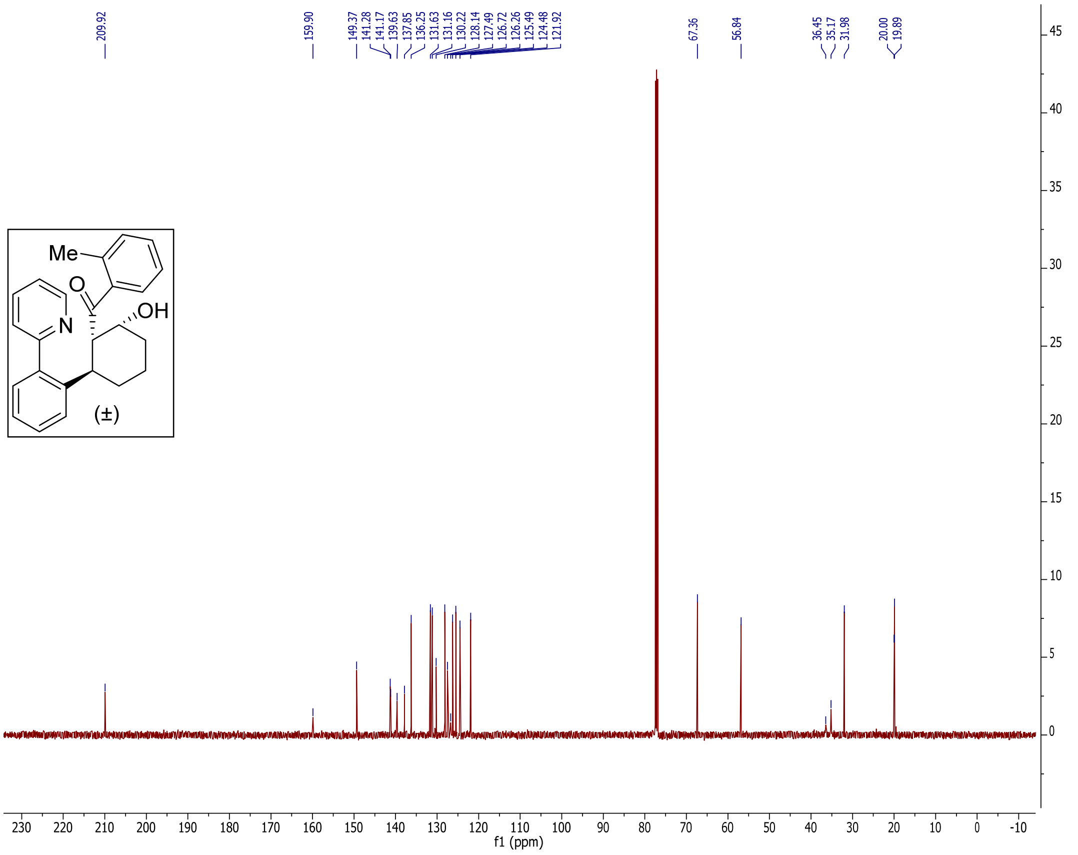

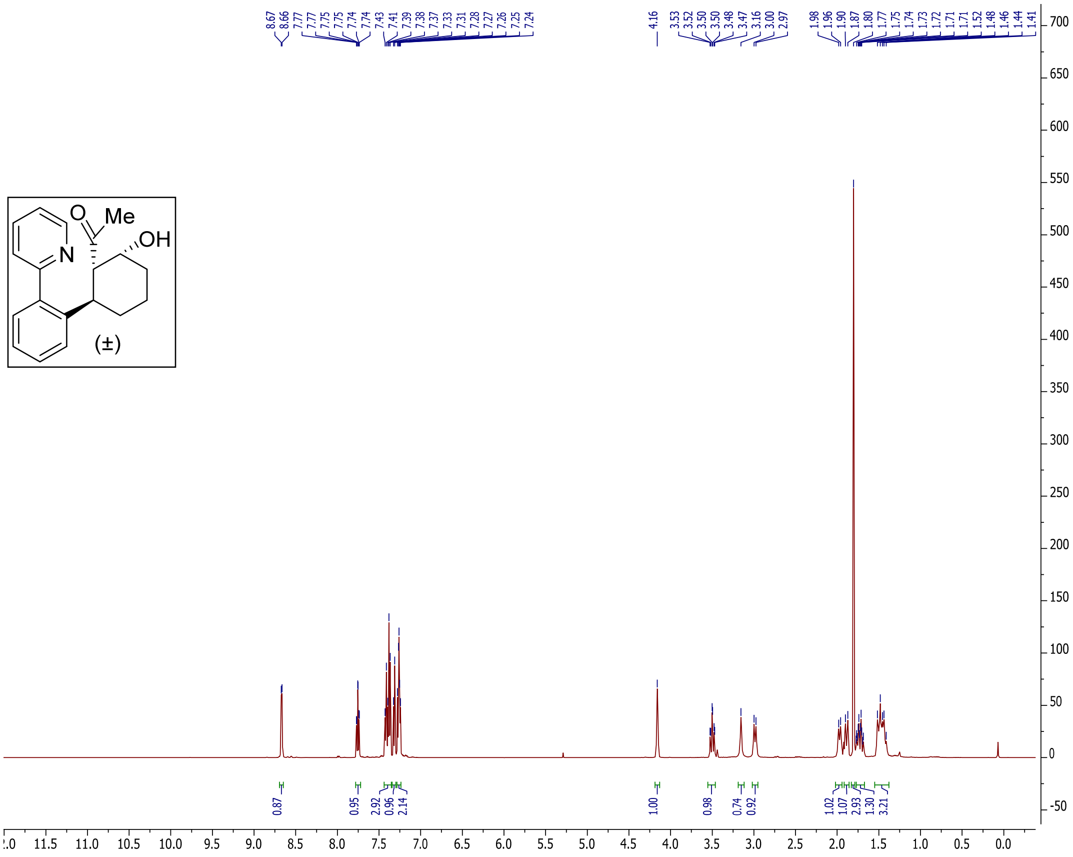
8.67 8.66 7.77 7.77 7.75 7.75 7.74 7.74 7.43 7.41 7.39 7.38 7.37 7.33 7.31 7.28 7.27 7.26 7.25 7.24
4.16
3.53 3.52 3.50 3.50 3.48 3.47 3.16 3.00 2.97
1.98 1.96 1.90 1.87 1.80 1.77 1.75 1.74 1.73 1.72 1.71 1.71 1.52 1.48 1.46 1.44 1.41
0.87 0.95 2.92 0.96 2.14 1.00 0.98 0.74 0.92 1.02 1.07 2.93 1.30 3.21
O
Me
OH
N
(±)

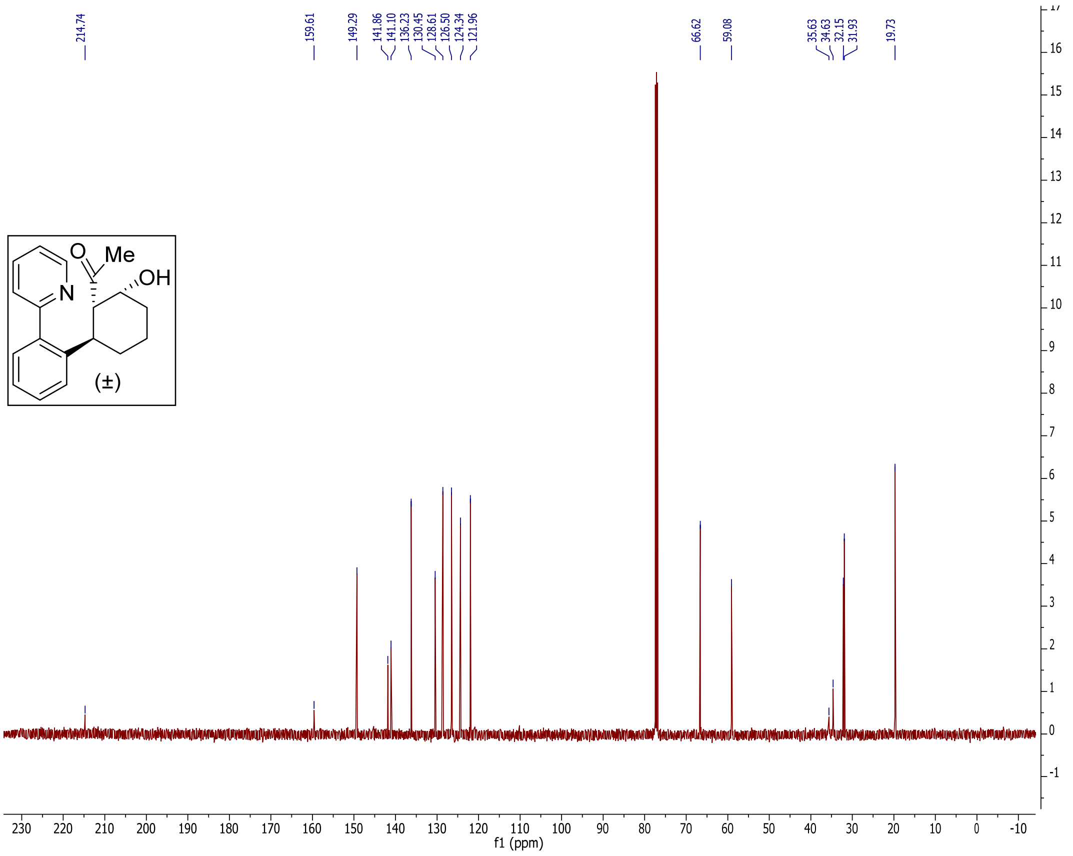
214.74
159.61
149.29
141.86 141.10 136.23 130.45 128.61 126.50 124.34 121.96
66.62
59.08
35.63 34.63 32.15 31.93
19.73
O
Me
OH
N
(±)
f1 (ppm)

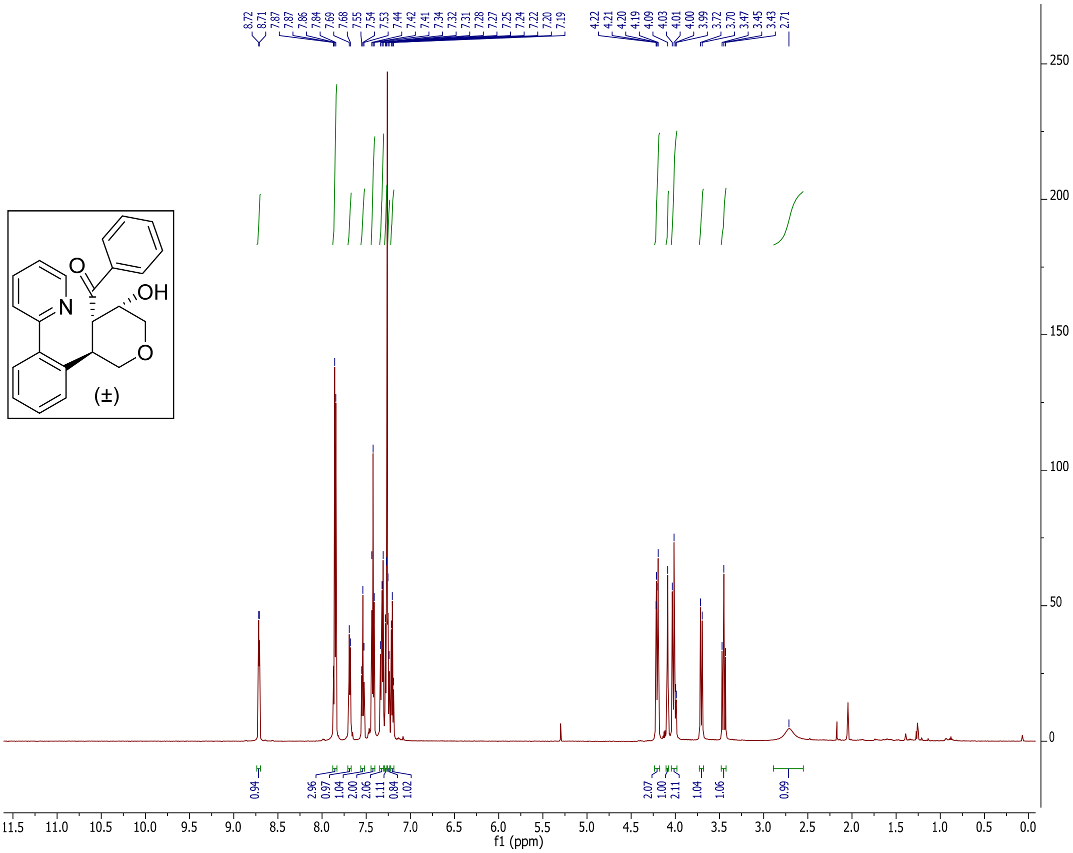
8.72
8.71
7.87
7.87
7.86
7.84
7.69
7.68
7.55
7.54
7.53
7.44
7.42
7.41
7.34
7.32
7.31
7.28
7.27
7.25
7.24
7.22
7.20
7.19
4.22
4.21
4.20
4.19
4.09
4.03
4.01
4.00
3.99
3.72
3.70
3.47
3.45
3.43
2.71
0.94
2.96
0.97
1.04
2.00
2.06
1.11
0.84
1.02
2.07
1.00
2.11
1.04
1.06
0.99
O
N
OH
O
(±)
f1 (ppm)

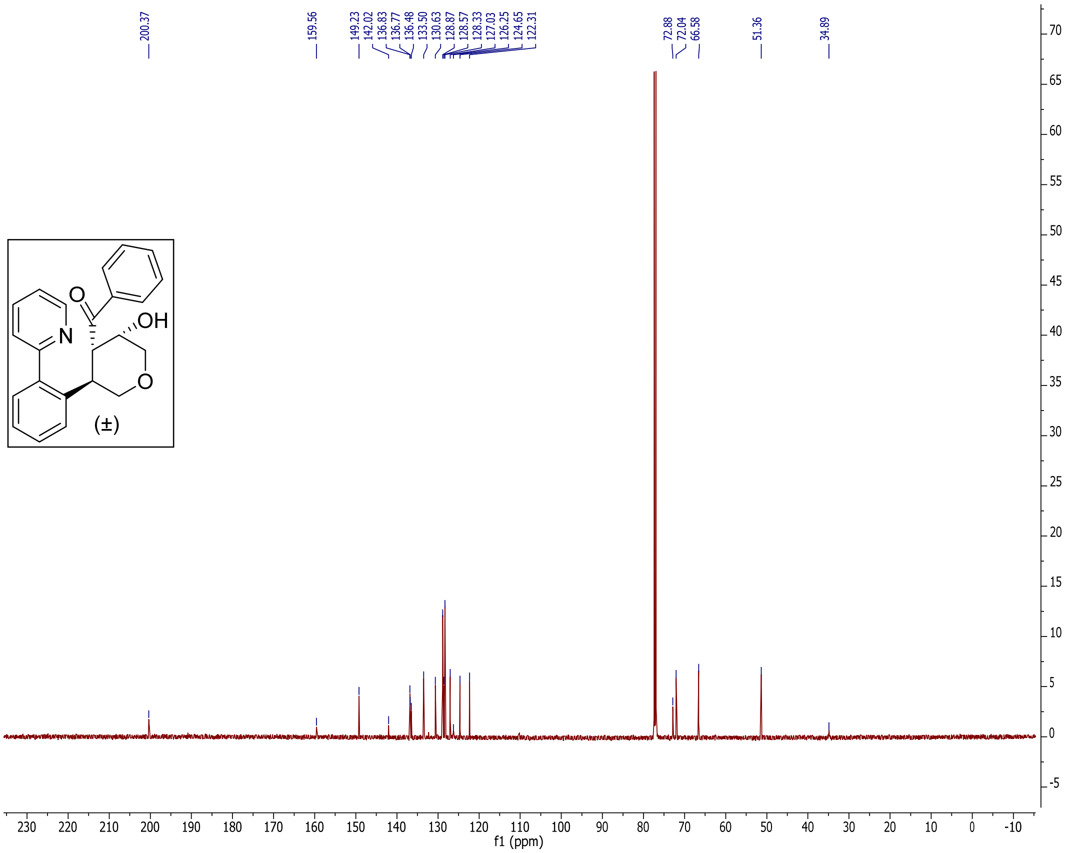
200.37
159.56
149.23
142.02
136.83
136.77
136.48
133.50
130.63
128.87
128.57
128.33
127.03
126.25
124.65
122.31
72.88
72.04
66.58
51.36
34.89
O
N
OH
O
(±)
f1 (ppm)

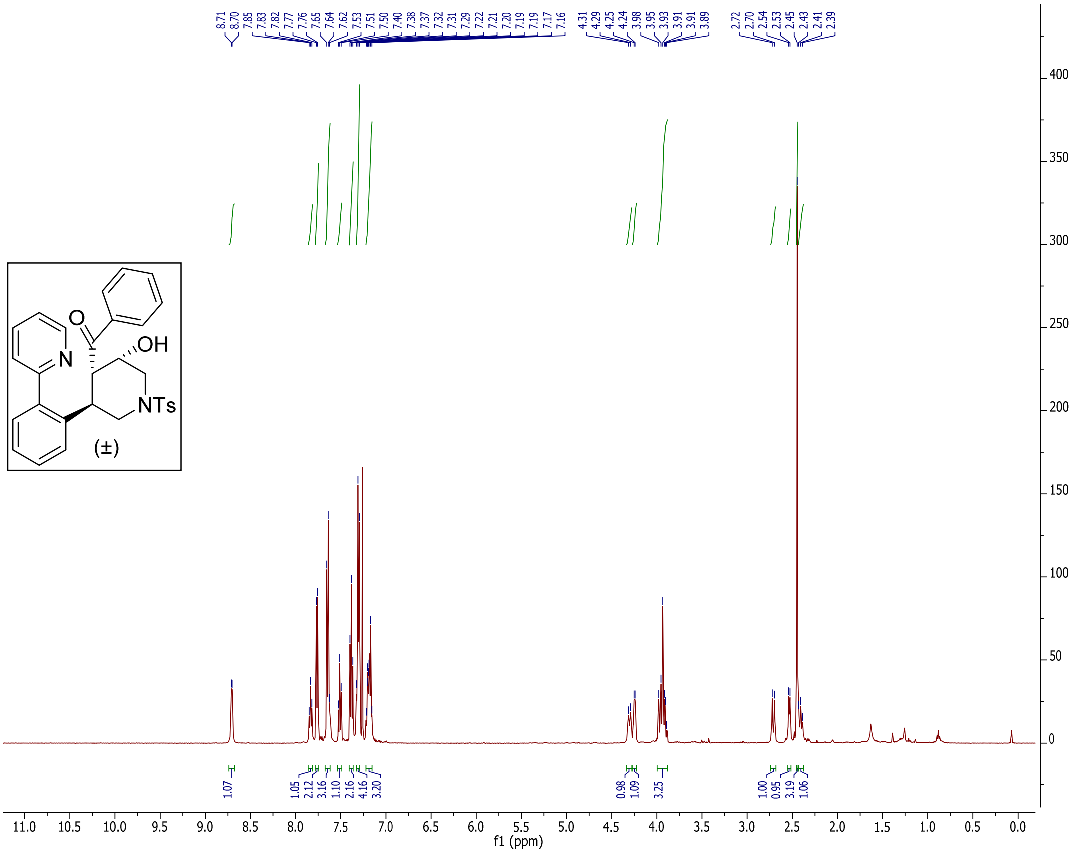
8.71
8.70
7.85
7.83
7.82
7.77
7.76
7.65
7.64
7.62
7.53
7.51
7.50
7.40
7.38
7.37
7.32
7.31
7.29
7.22
7.21
7.20
7.19
7.19
7.17
7.16
4.31
4.29
4.25
4.24
3.98
3.95
3.93
3.93
3.91
3.91
3.89
2.72
2.70
2.54
2.53
2.45
2.43
2.41
2.39
O
OH
N
NTs
(±)
1.07
1.05
2.12
3.16
1.10
2.16
4.16
3.20
0.98
1.09
3.25
1.00
0.95
3.19
1.06
f1 (ppm)

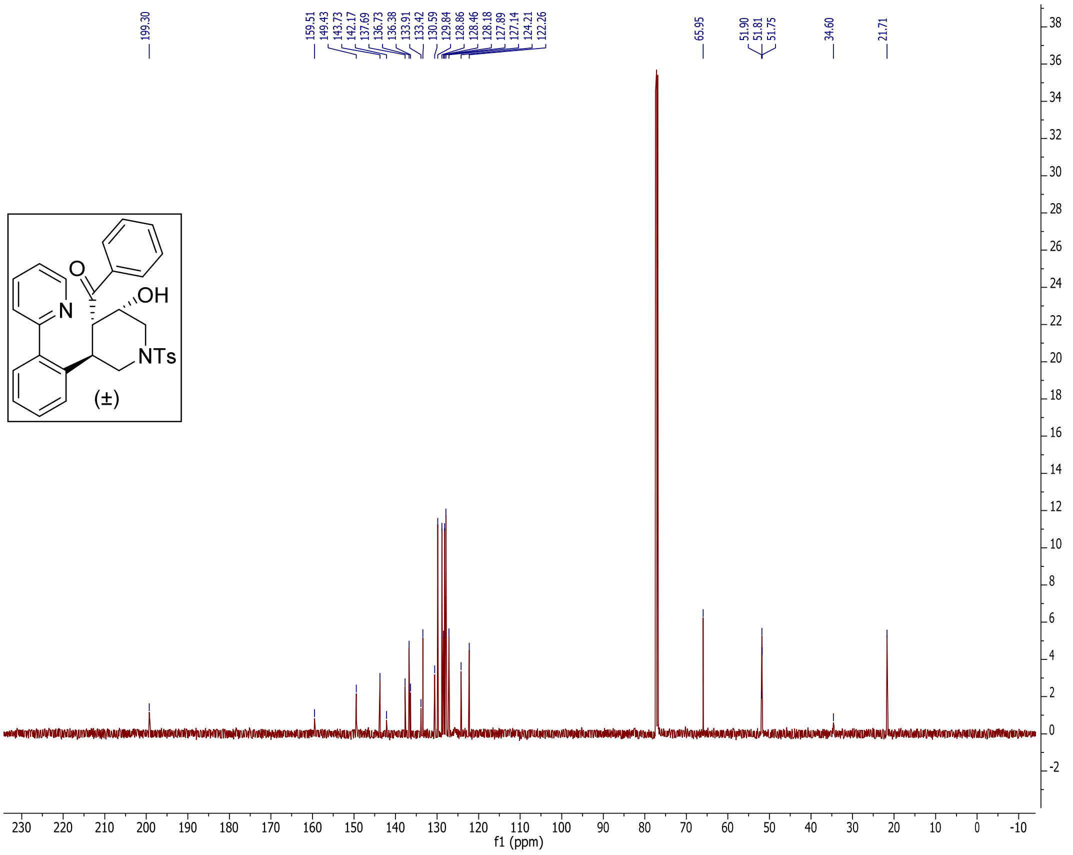
199.30
159.51
149.43
143.73
142.17
137.69
136.73
136.38
133.91
133.42
130.59
129.84
128.86
128.46
128.18
127.89
127.14
124.21
122.26
65.95
51.90
51.81
51.75
34.60
21.71
O
OH
N
NTs
(±)
f1 (ppm)

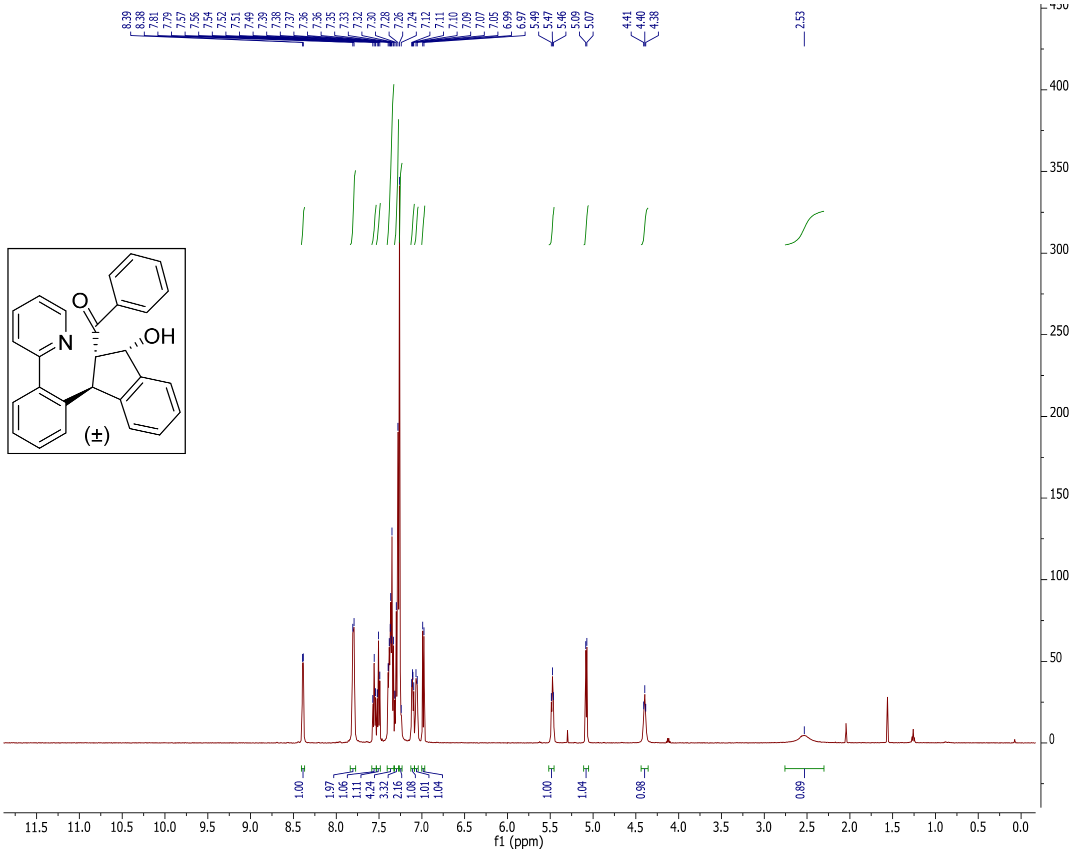
8.39 8.38 7.81 7.79 7.57 7.56 7.54 7.52 7.51 7.49 7.39 7.38 7.37 7.36 7.36 7.35 7.33 7.32 7.30 7.28 7.26 7.24 7.12 7.11 7.10 7.09 7.07 7.05 6.99 6.97 5.49 5.47 5.46 5.09 5.07 4.41 4.40 4.38 2.53
1.00 1.97 1.06 1.11 4.24 3.32 2.16 1.08 1.01 1.04 1.00 1.04 0.98 0.89
O
N
OH
(±)
f1 (ppm)

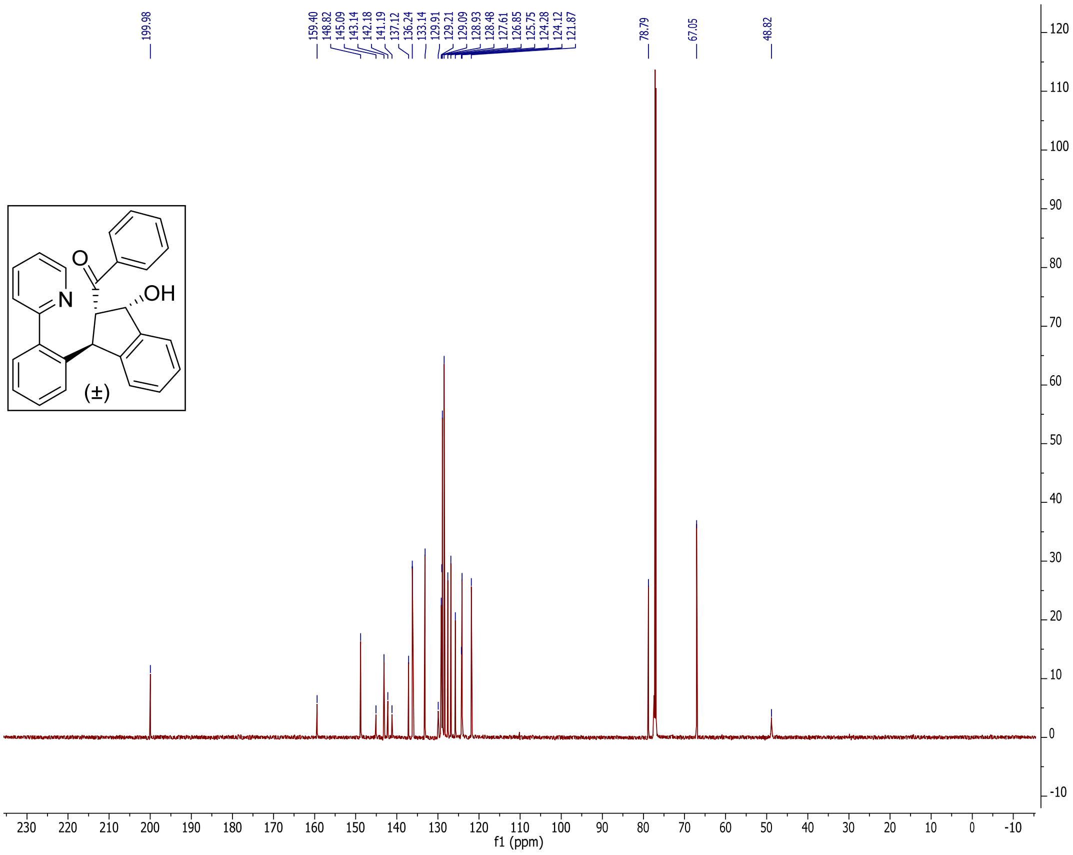
199.98 159.40 148.82 145.09 143.14 142.18 141.19 137.12 136.24 133.14 129.91 129.21 129.09 128.93 128.48 127.61 126.85 125.75 124.28 124.12 121.87 78.79 67.05 48.82
O
N
OH
(±)
f1 (ppm)

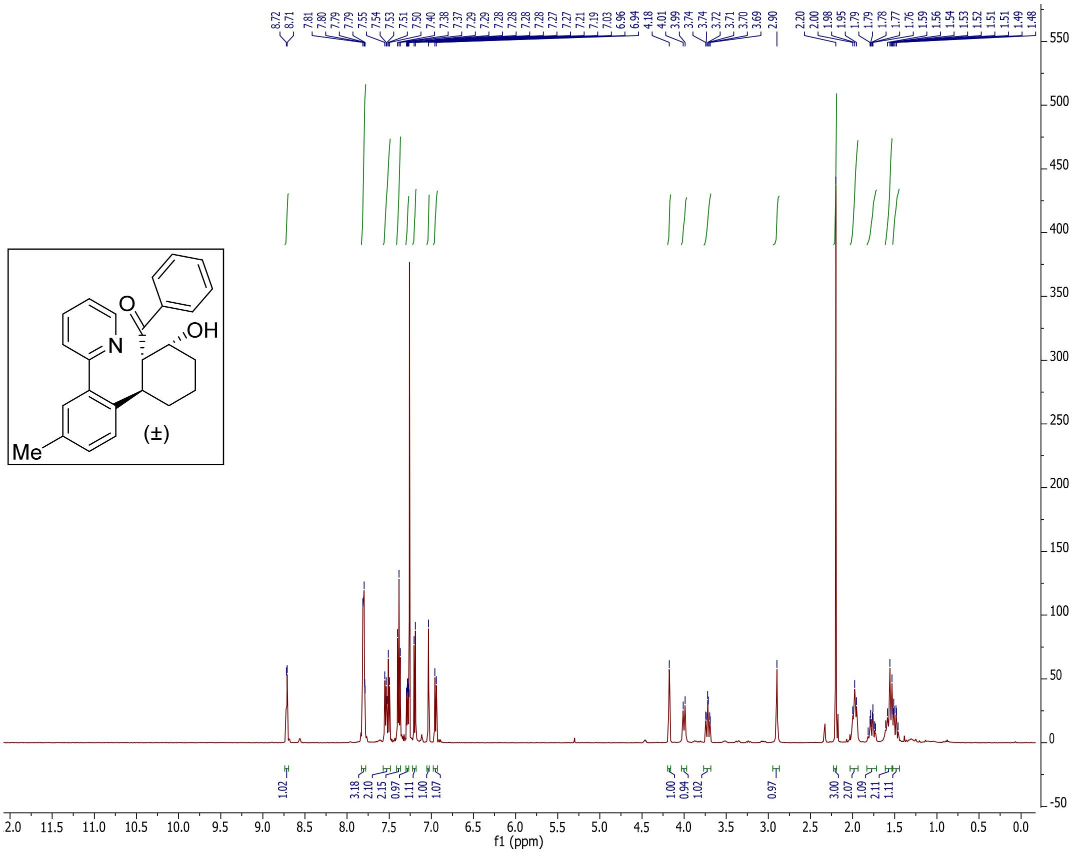
8.72 8.71 7.81 7.80 7.79 7.79 7.55 7.54 7.53 7.51 7.50 7.40 7.38 7.37 7.29 7.29 7.28 7.28 7.28 7.27 7.21 7.19 7.03 6.96 6.94 4.18 4.01 3.99 3.74 3.74 3.72 3.71 3.70 3.69 2.90 2.20 2.00 1.98 1.95 1.79 1.79 1.78 1.77 1.76 1.59 1.56 1.54 1.53 1.52 1.51 1.51 1.49 1.48
O
N
OH
Me
(±)
1.02 3.18 2.10 2.15 0.97 1.11 1.00 1.07 1.00 0.94 1.02 0.97 3.00 2.07 1.09 2.11 1.11
f1 (ppm)

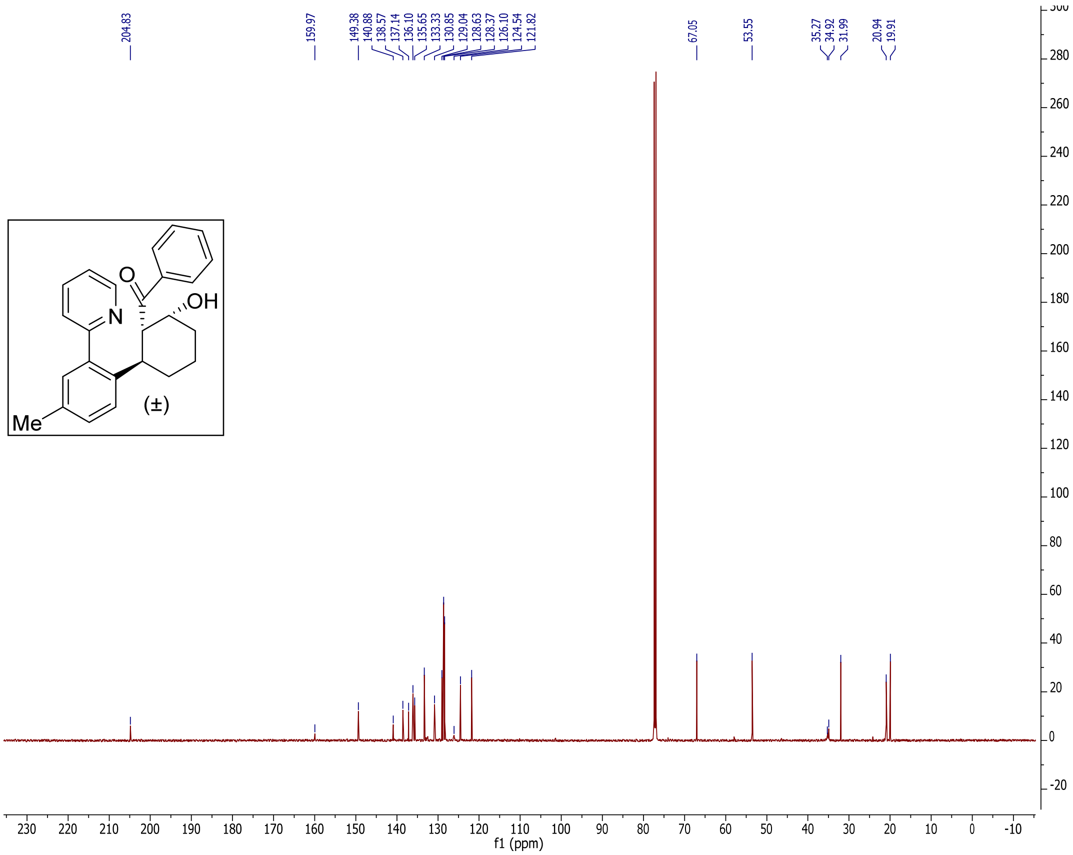
204.83 159.97 149.38 140.88 138.57 137.14 136.10 135.65 133.33 130.85 129.04 128.63 128.37 126.10 124.54 121.82 67.05 53.55 35.27 34.92 31.99 20.94 19.91
O
N
OH
Me
(±)
f1 (ppm)

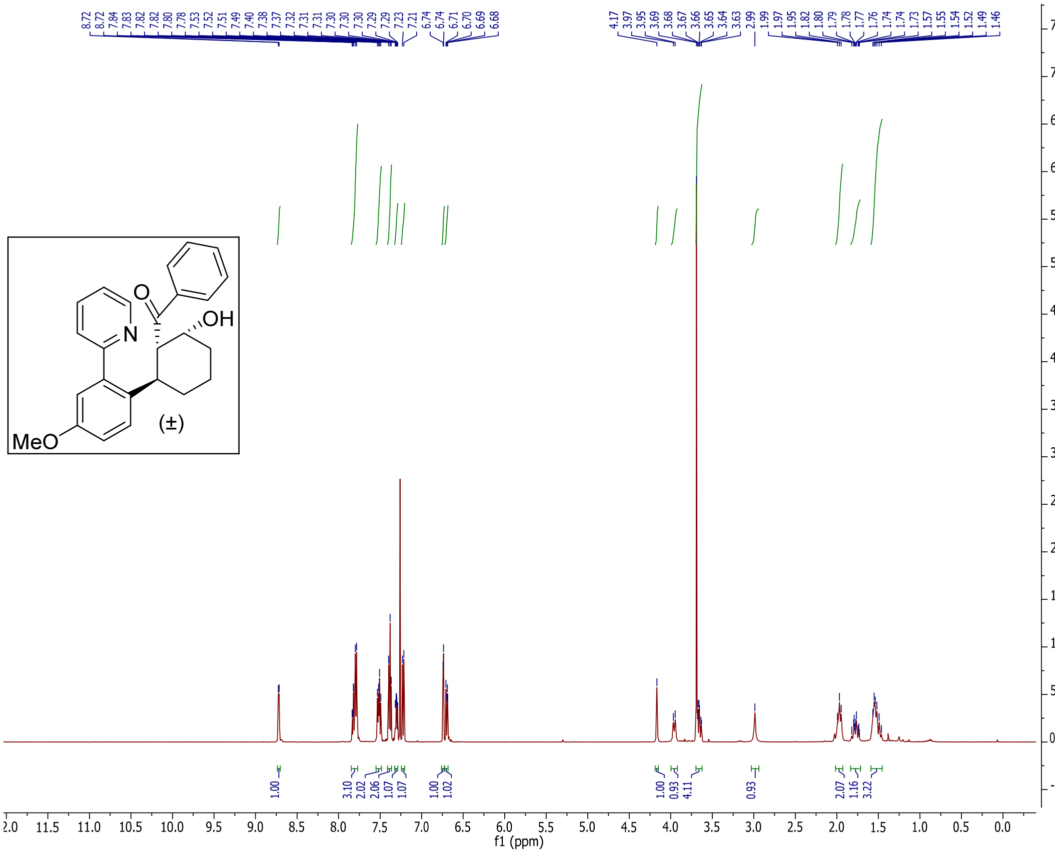
O
N
OH
MeO
(±)
f1 (ppm)

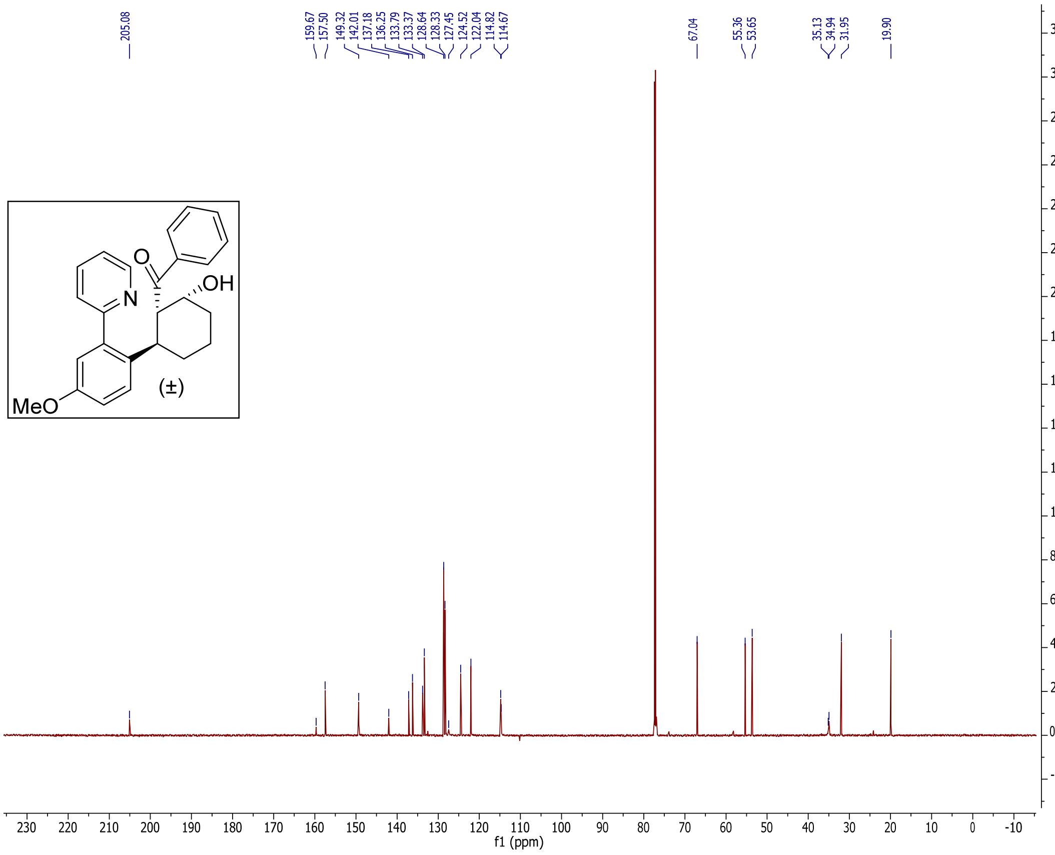
205.08
159.67
157.50
149.32
142.01
137.18
136.25
133.79
133.37
128.64
128.33
127.45
124.52
122.04
114.82
114.67
67.04
55.36
53.65
35.13
34.94
31.95
19.90
O
N
OH
MeO
(±)
f1 (ppm)

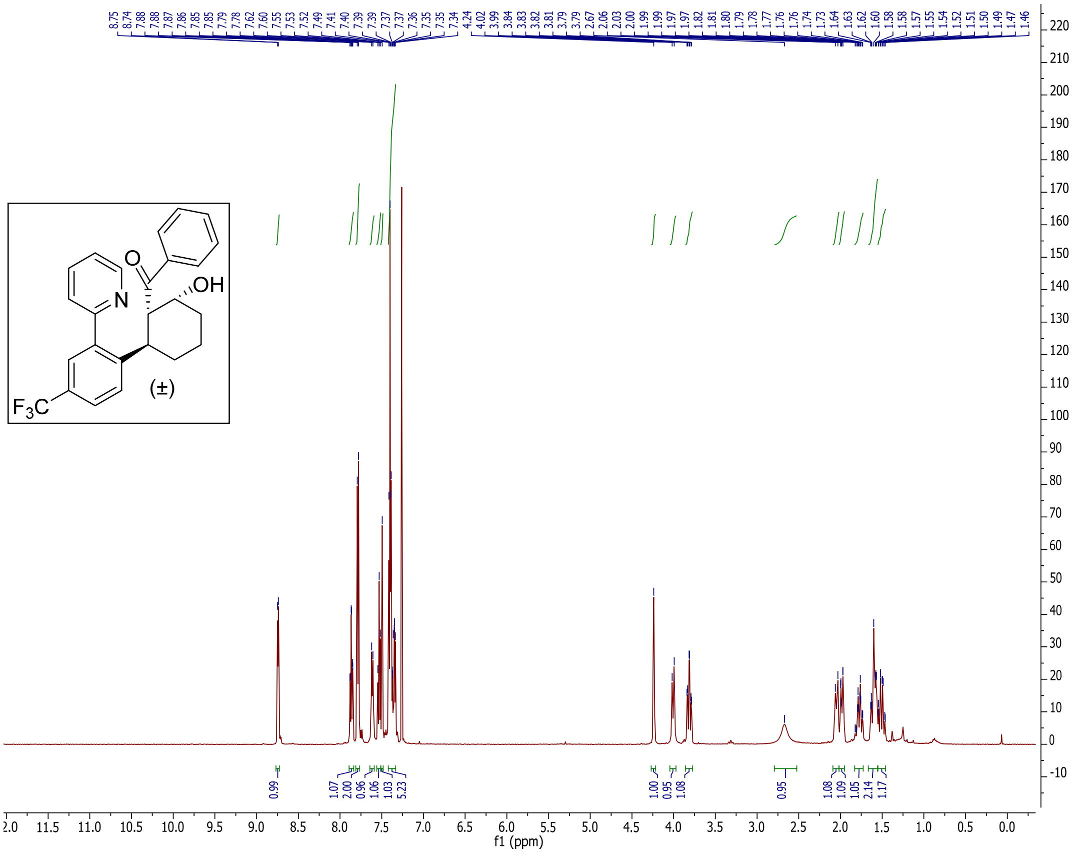

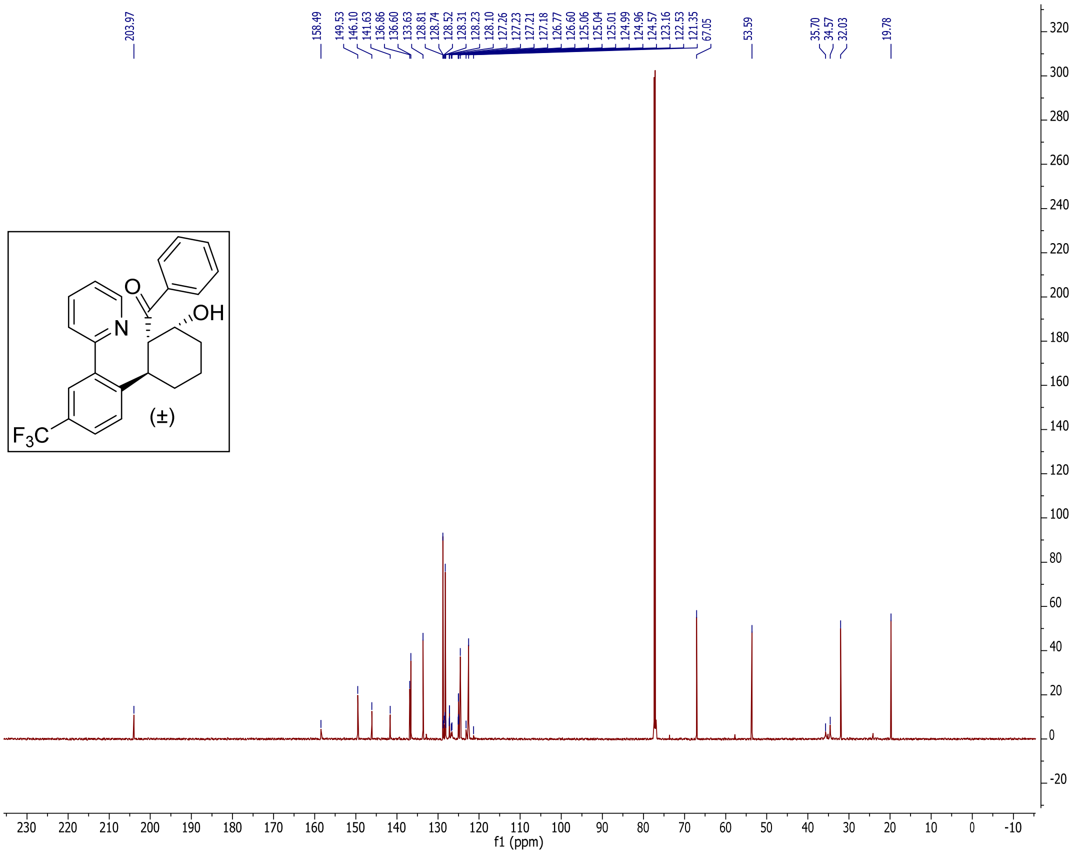

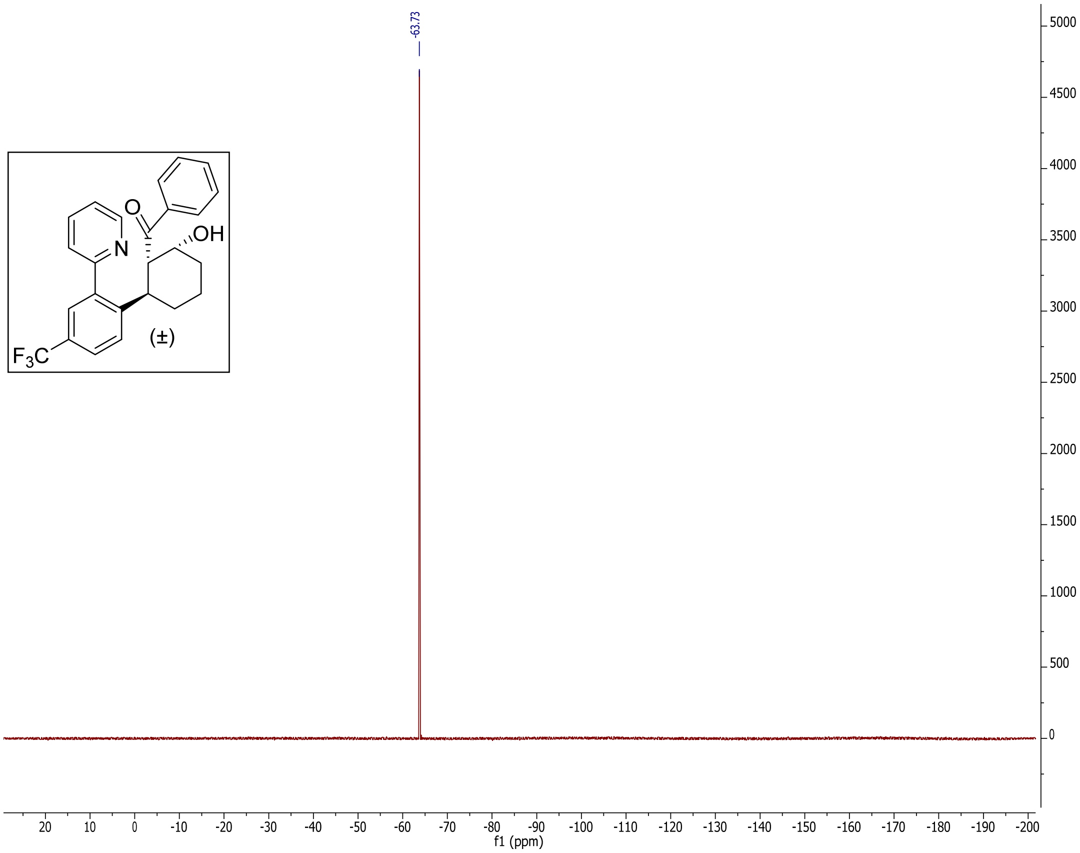
-63.73
O
OH
N
F3C
(±)
5000
4500
4000
3500
3000
2500
2000
1500
1000
500
0
20
10
0
-10
-20
-30
-40
-50
-60
-70
-80
-90
-100
-110
-120
-130
-140
-150
-160
-170
-180
-190
-200
f1 (ppm)

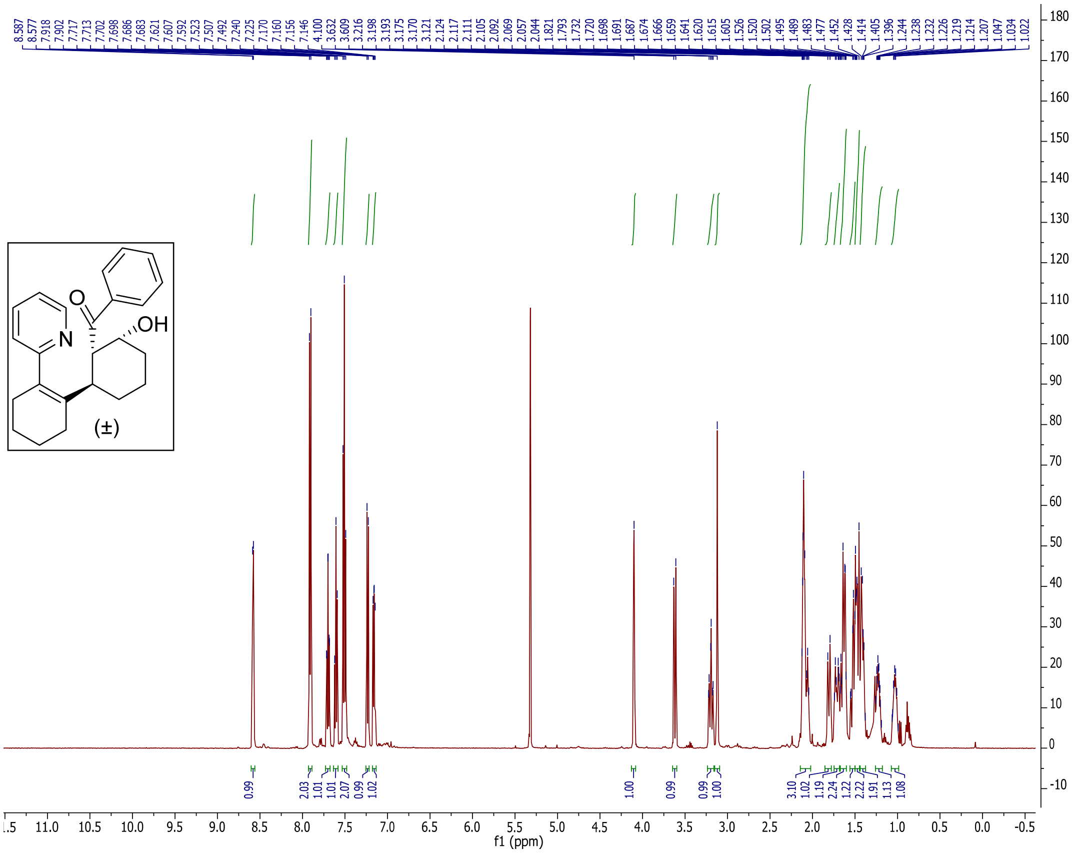

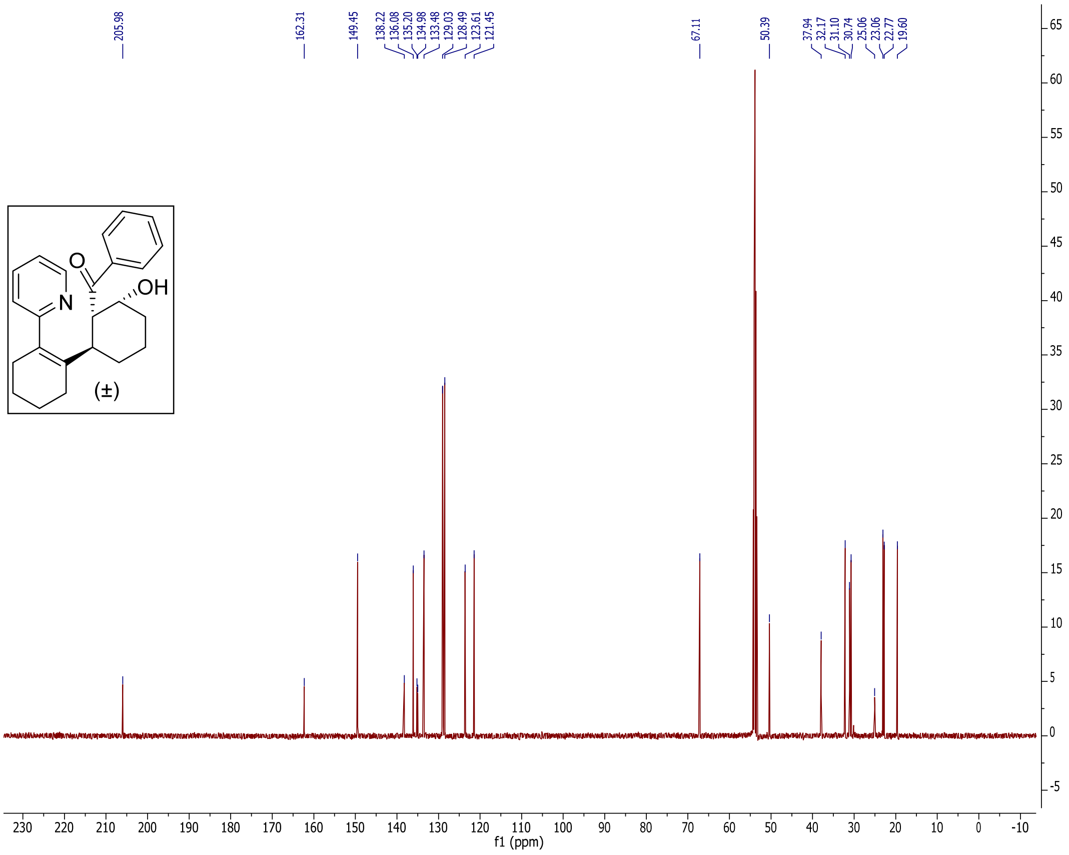

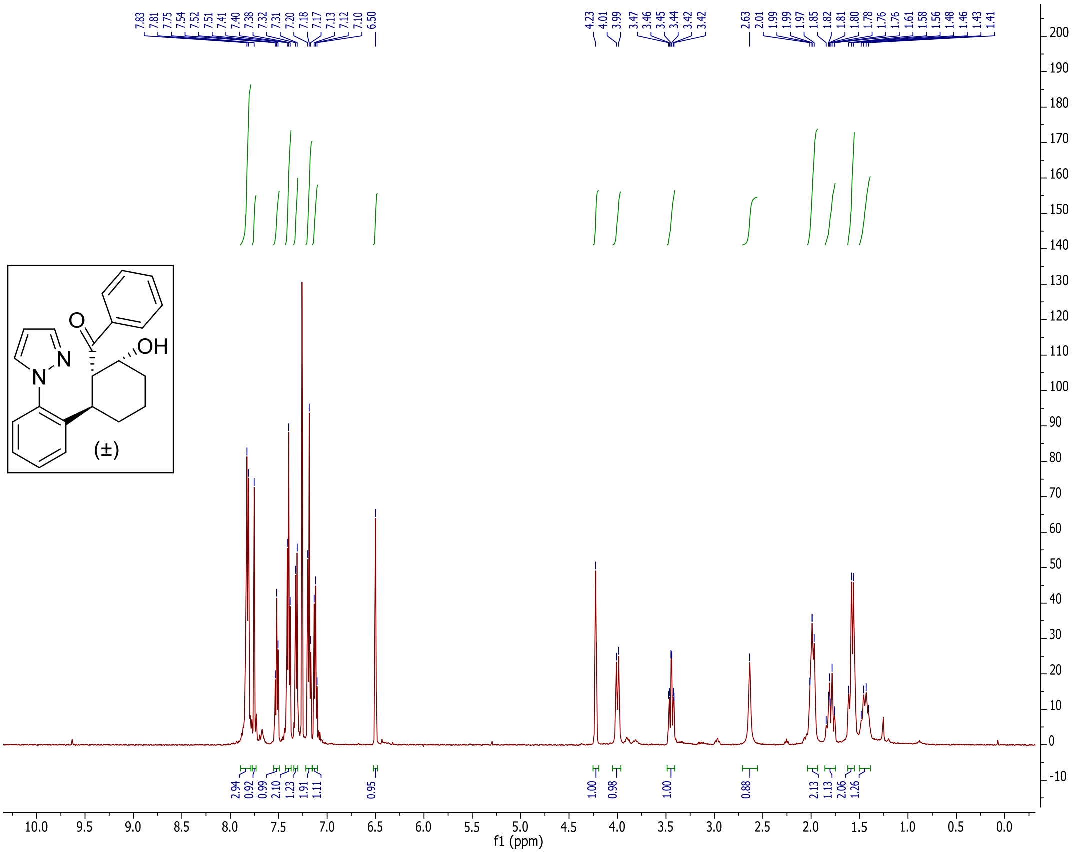

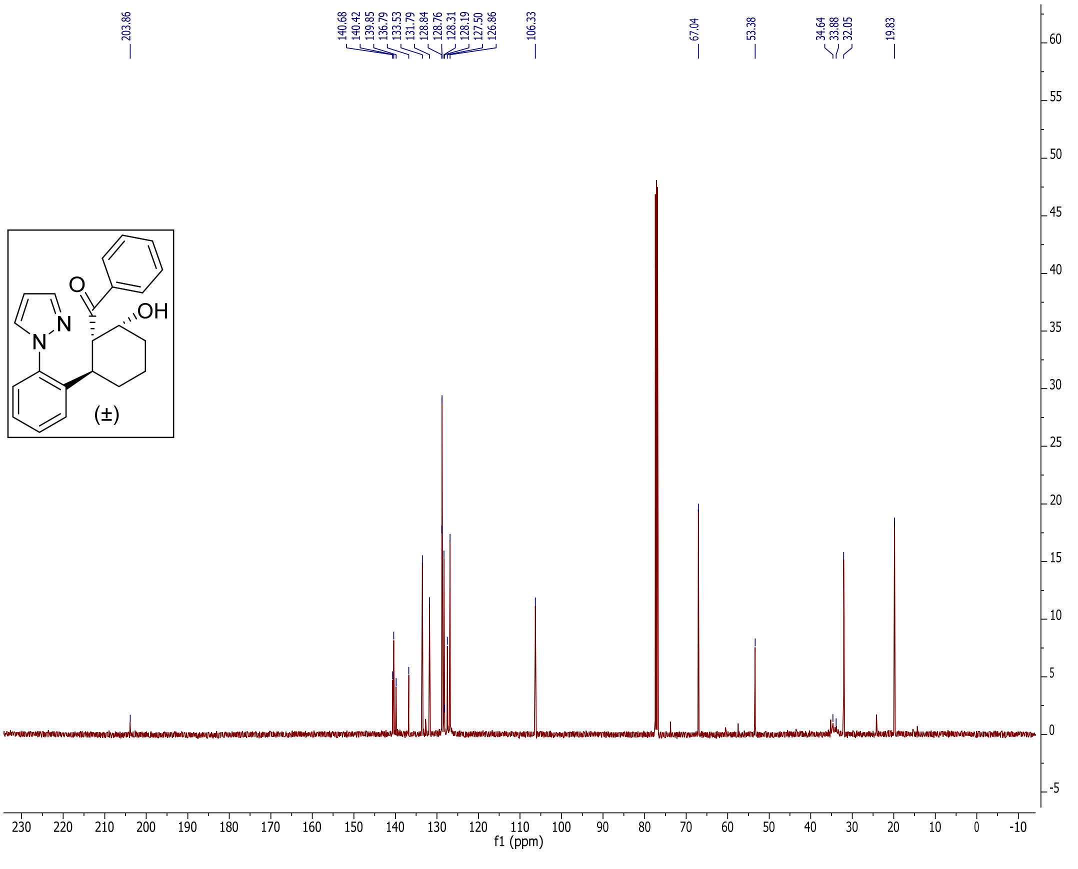

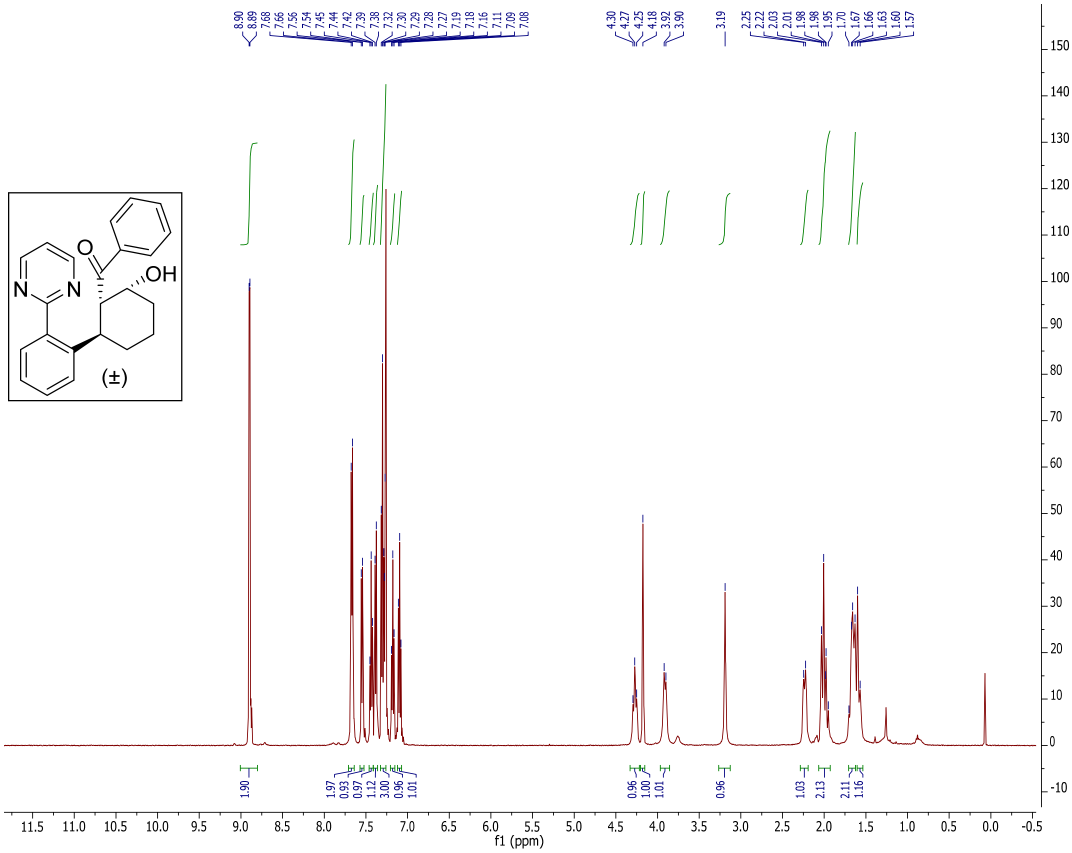

8.90 8.89 7.68 7.66 7.56 7.54 7.45 7.44 7.42 7.39 7.38 7.32 7.30 7.29 7.28 7.27 7.19 7.18 7.16 7.11 7.09 7.08
4.30 4.27 4.25 4.18 3.92 3.90
3.19
2.25 2.22 2.03 2.01 1.98 1.98 1.95 1.70 1.67 1.66 1.63 1.60 1.57
O
N
N
OH
(±)
1.90
1.97 0.93 0.97 1.12 3.00 0.96 1.01
0.96 1.00 1.01
0.96
1.03 2.13
2.11 1.16
f1 (ppm)


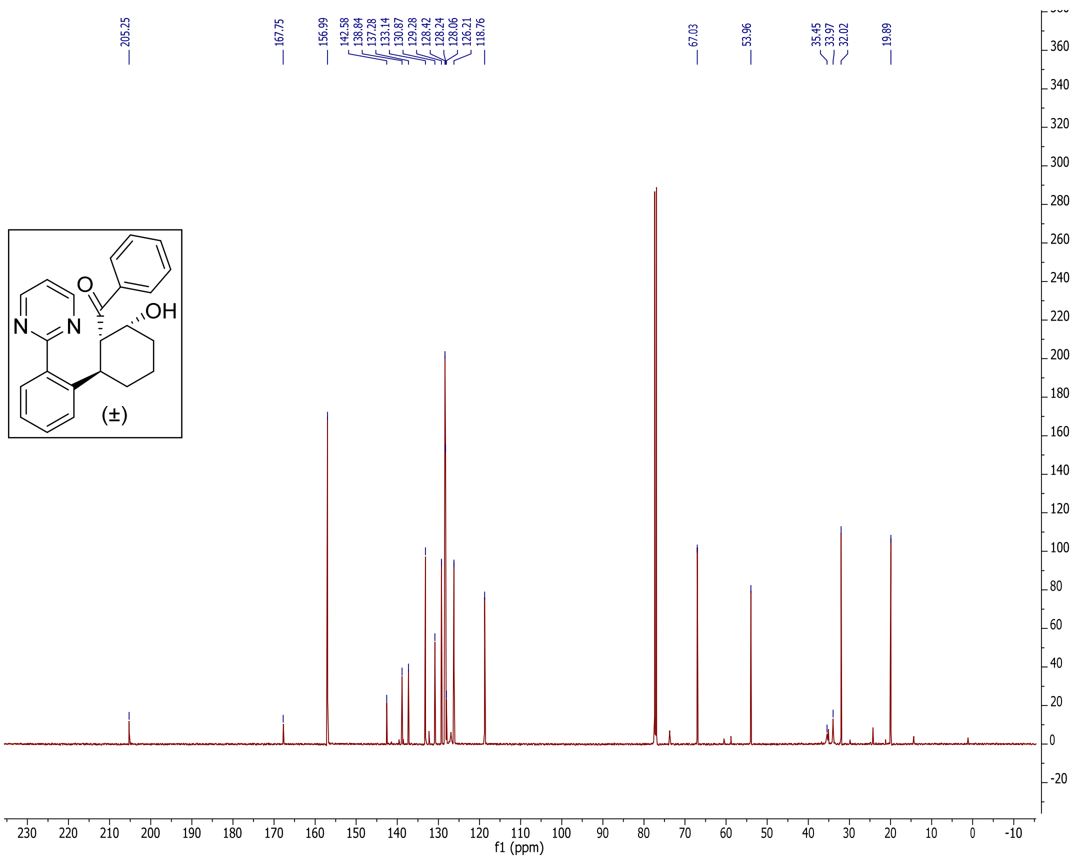

205.25
167.75
156.99
142.58 138.84 137.28 133.14 130.87 129.28 128.42 128.24 128.06 126.21 118.76
67.03
53.96
35.45 33.97 32.02
19.89
O
N
N
OH
(±)
f1 (ppm)

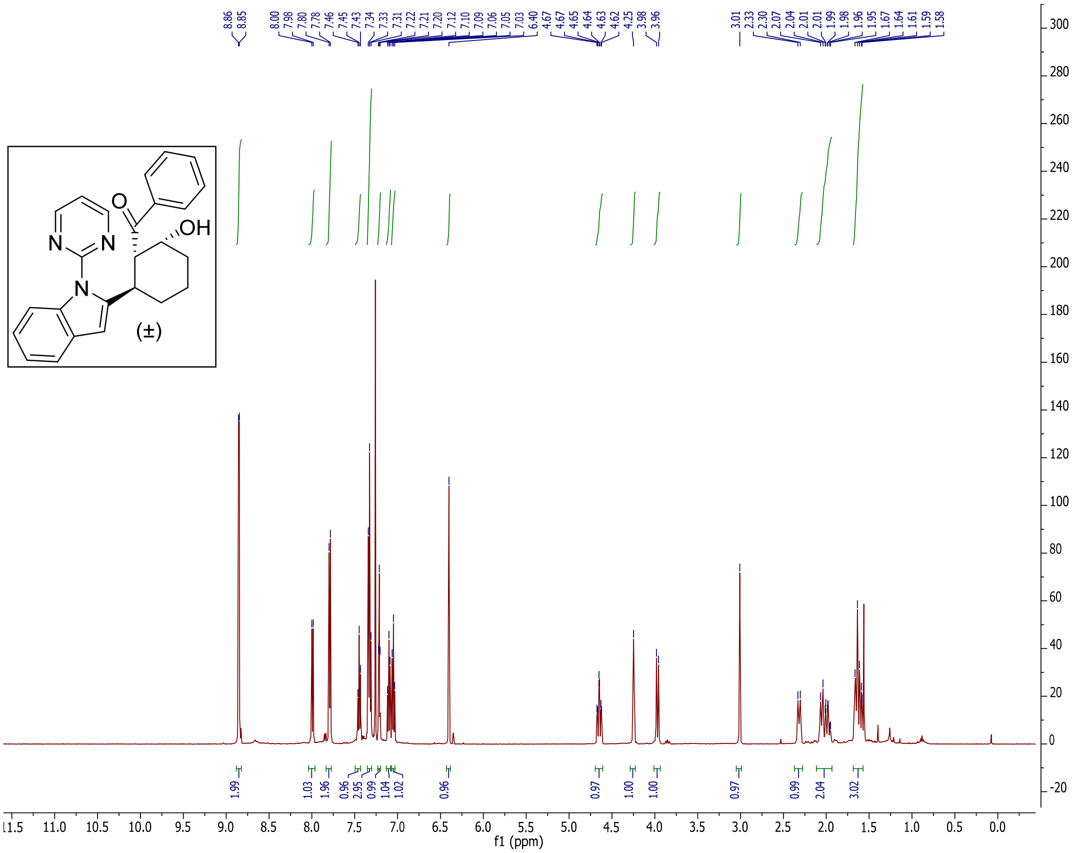
8.86
8.85
8.00
7.98
7.80
7.78
7.46
7.45
7.43
7.34
7.33
7.31
7.22
7.21
7.20
7.12
7.10
7.09
7.06
7.05
7.03
6.40
4.67
4.67
4.65
4.64
4.63
4.62
4.25
3.98
3.96
3.01
2.33
2.30
2.07
2.04
2.01
1.99
1.98
1.96
1.95
1.67
1.64
1.61
1.59
1.58
O
N
N
OH
N
(±)
1.99
1.03
1.96
0.96
2.95
0.99
1.04
1.02
0.96
0.97
1.00
1.00
0.97
0.99
2.04
3.02
f1 (ppm)

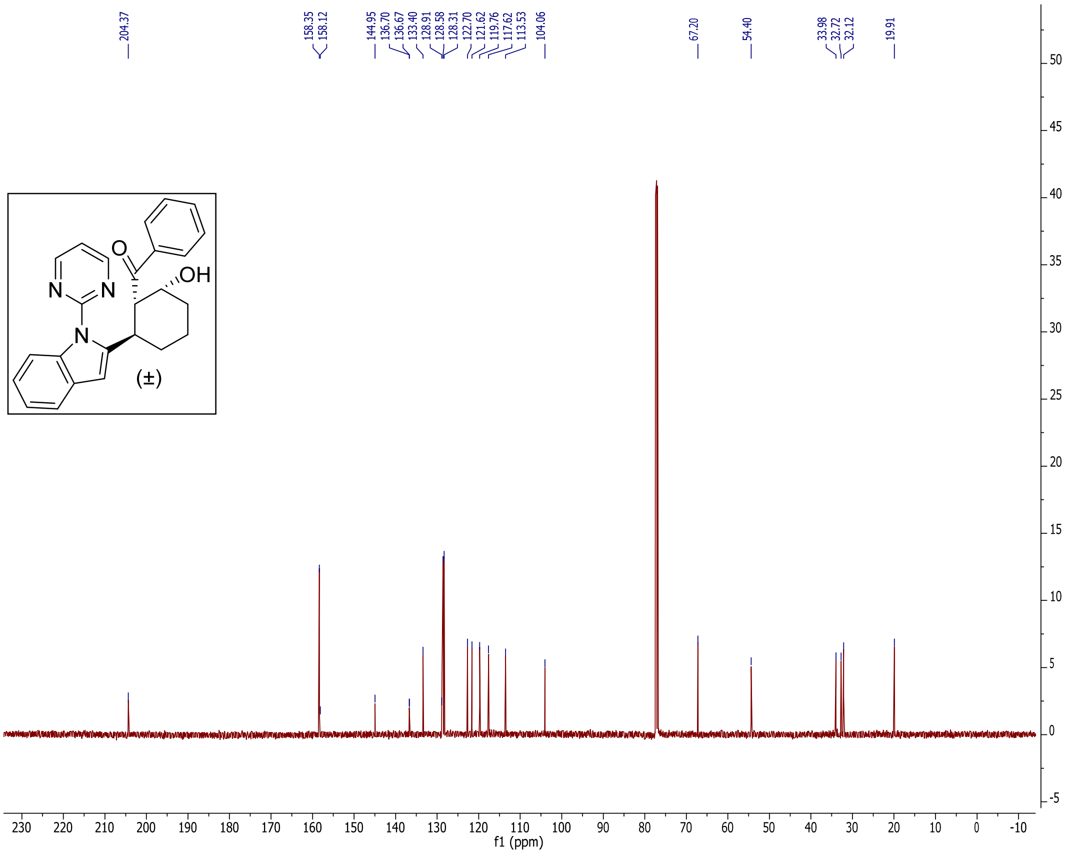
204.37
158.35
158.12
144.95
136.70
136.67
133.40
128.91
128.58
128.31
122.70
121.62
119.76
117.62
113.53
104.06
67.20
54.40
33.98
32.72
32.12
19.91
O
N
N
OH
N
(±)
f1 (ppm)

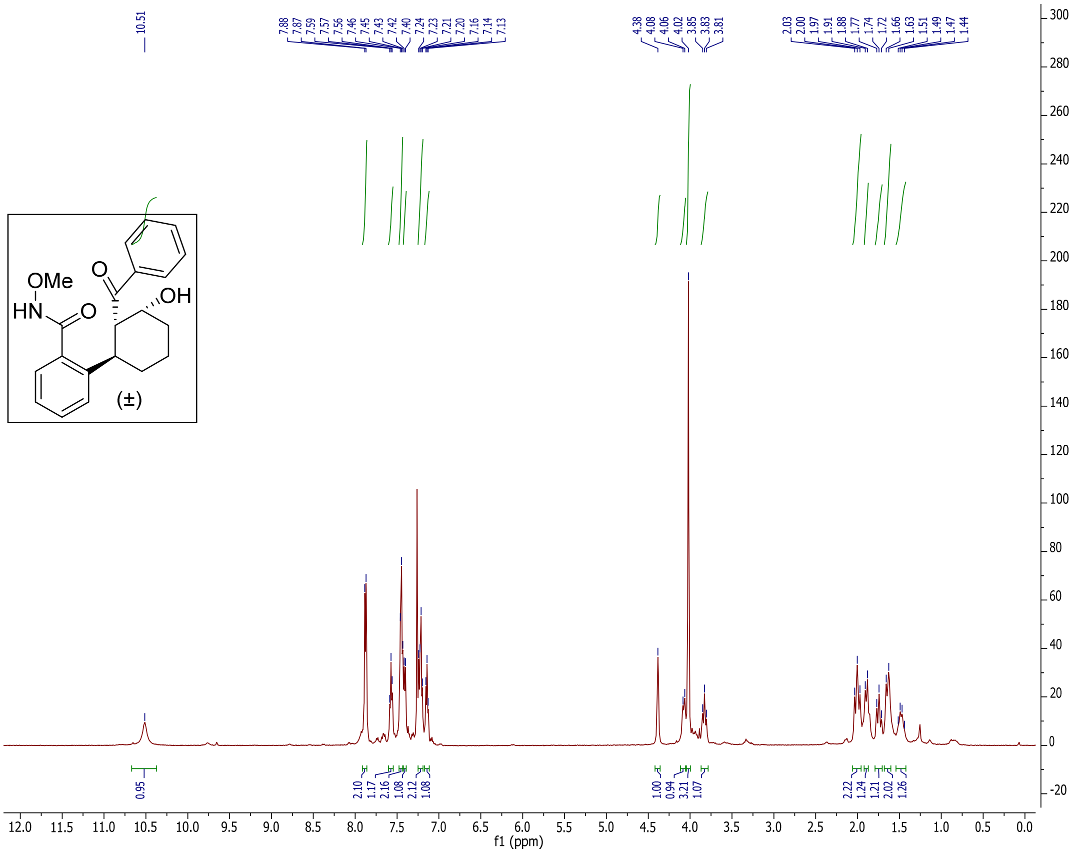
10.51
7.88 7.87 7.59 7.57 7.56 7.46 7.45 7.43 7.42 7.40 7.24 7.23 7.21 7.20 7.16 7.14 7.13
4.38 4.08 4.06 4.02 3.85 3.83 3.81
2.03 2.00 1.97 1.91 1.88 1.77 1.74 1.72 1.66 1.63 1.51 1.49 1.47 1.44
OMe
HN
O
OH
(±)
0.95
2.10 1.17 2.16 1.08 2.12 1.08
1.00 0.94 3.21 1.07
2.22 1.24 1.21 2.02 1.26
f1 (ppm)

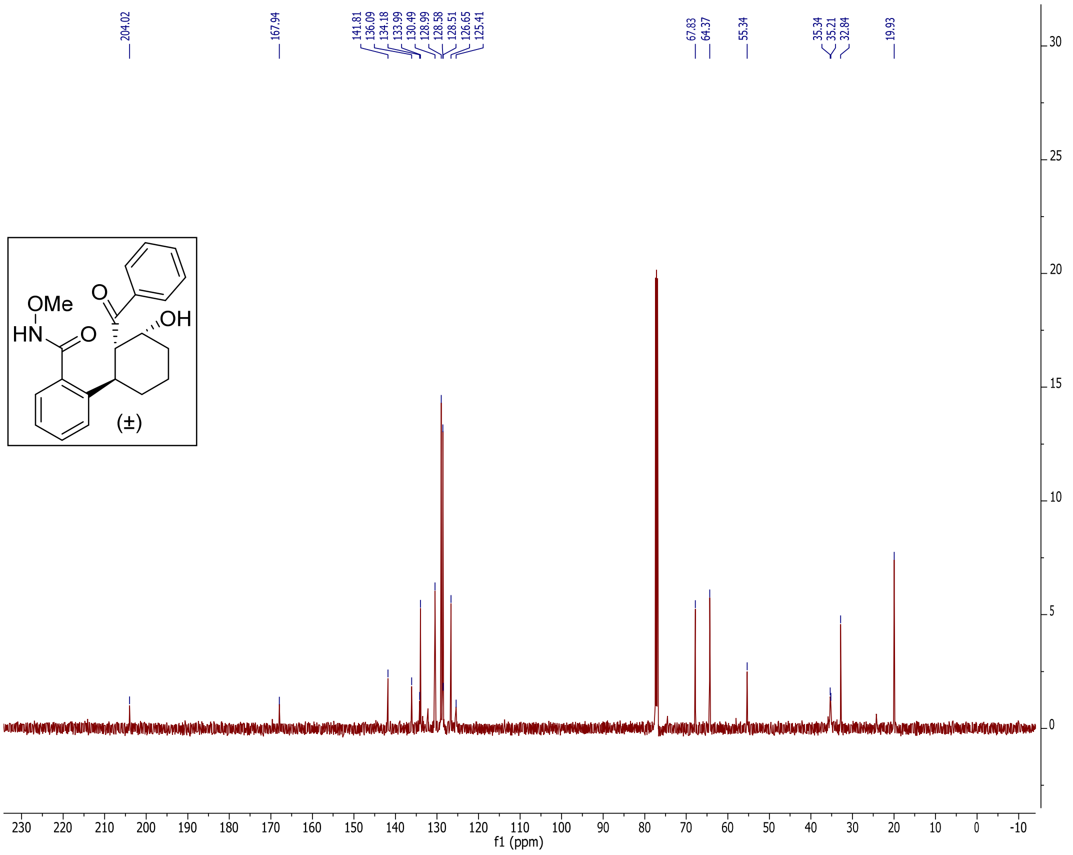
204.02
167.94
141.81 136.09 134.18 133.99 130.49 128.99 128.58 128.51 126.65 125.41
67.83 64.37
55.34
35.34 35.21 32.84
19.93
OMe
HN
O
OH
(±)
f1 (ppm)

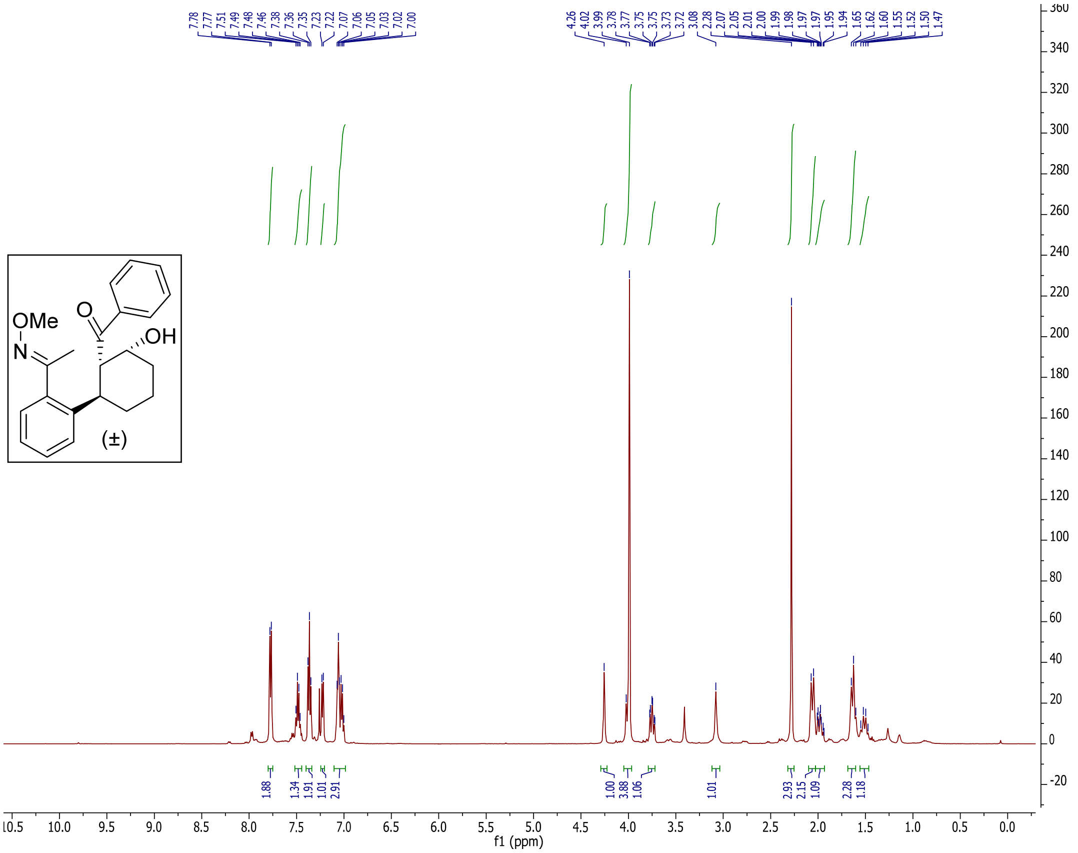

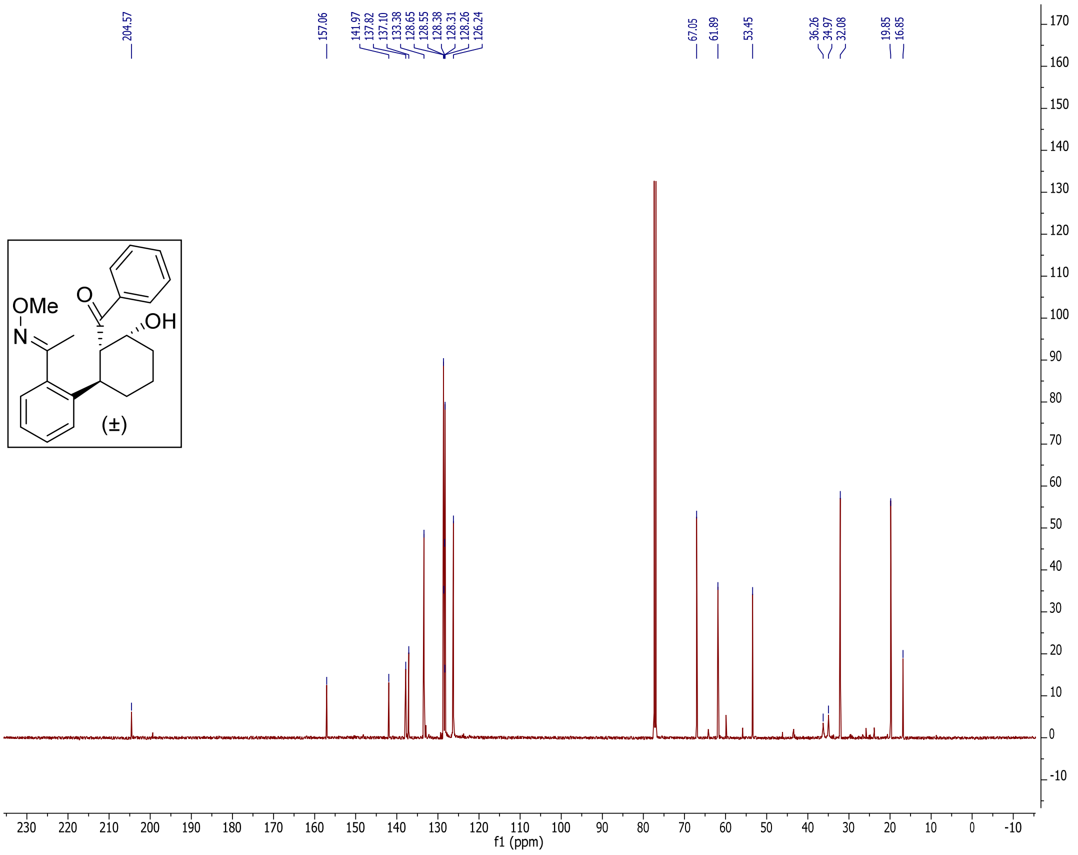

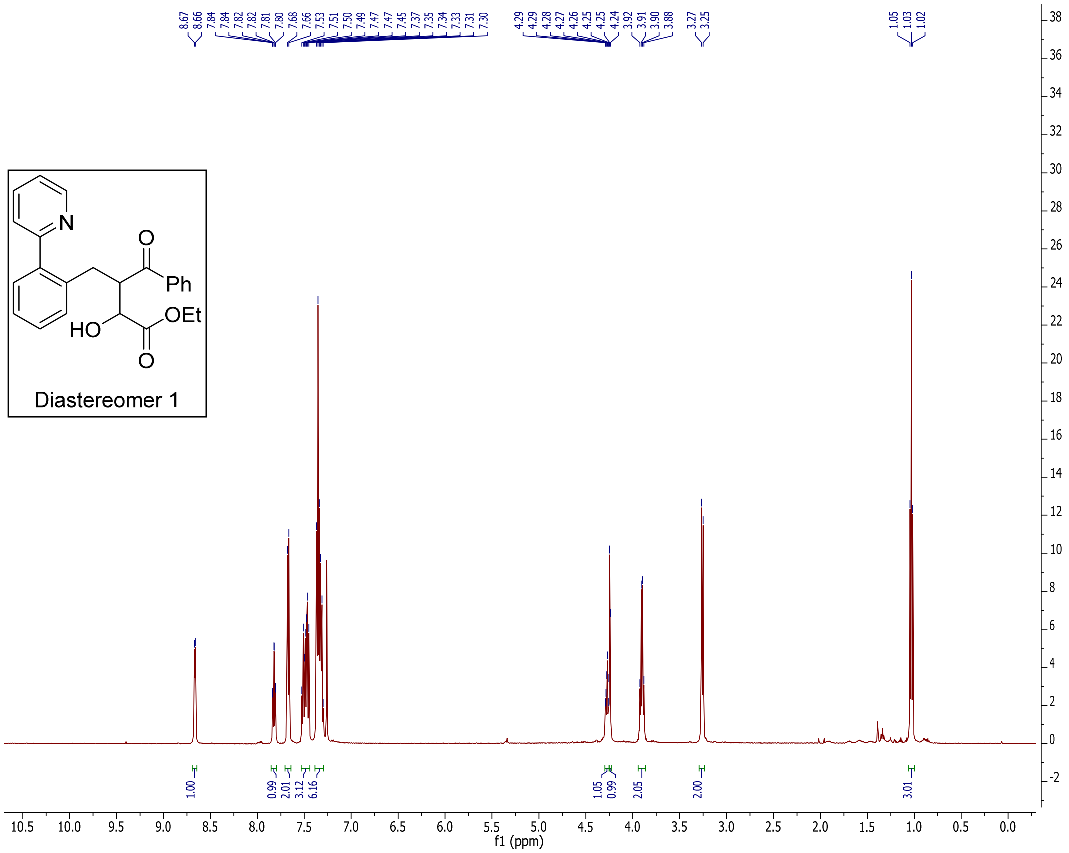
8.67
8.66
7.84
7.82
7.82
7.81
7.80
7.68
7.66
7.53
7.51
7.50
7.49
7.47
7.47
7.45
7.37
7.35
7.34
7.33
7.31
7.30
4.29
4.29
4.28
4.27
4.26
4.25
4.25
4.24
3.92
3.91
3.90
3.88
3.27
3.25
1.05
1.03
1.02
N
O
Ph
OEt
HO
O
Diastereomer 1
1.00
0.99
2.01
3.12
6.16
1.05
0.99
2.05
2.00
3.01
f1 (ppm)

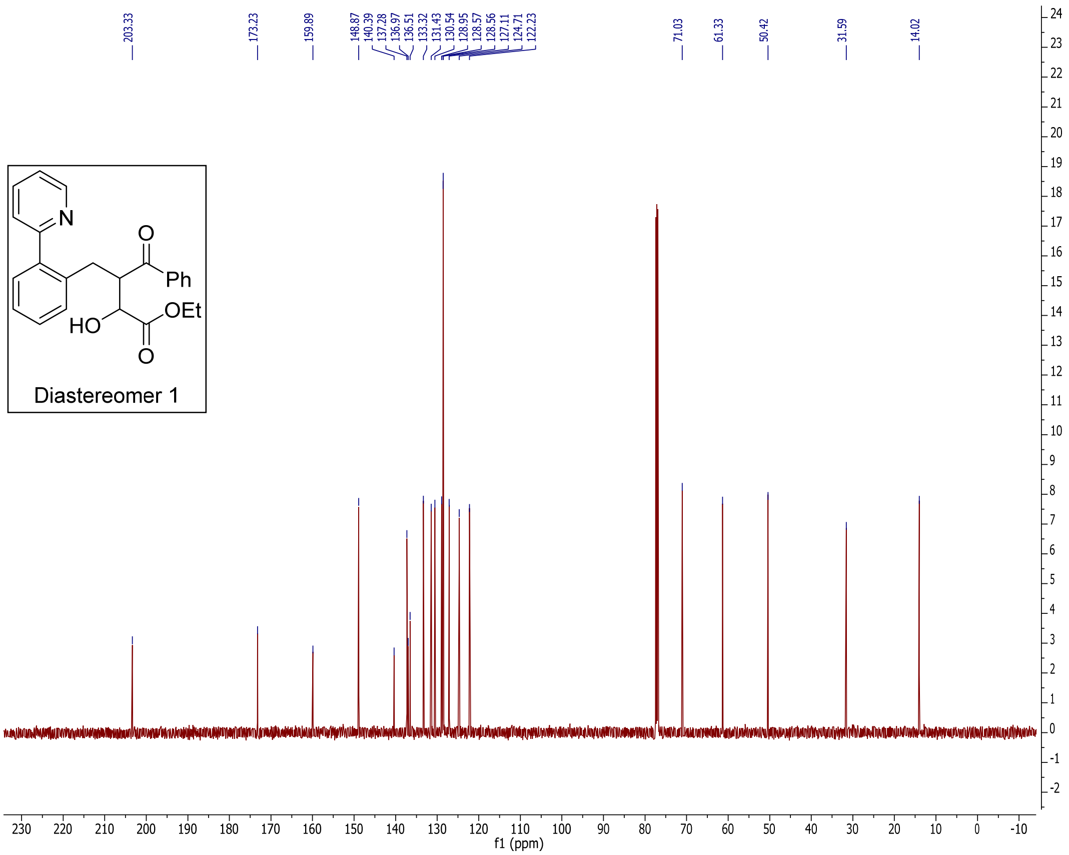
203.33
173.23
159.89
148.87
140.39
137.28
136.97
136.51
133.32
131.43
130.54
128.95
128.57
128.56
127.11
124.71
122.23
71.03
61.33
50.42
31.59
14.02
N
O
Ph
OEt
HO
O
Diastereomer 1
f1 (ppm)

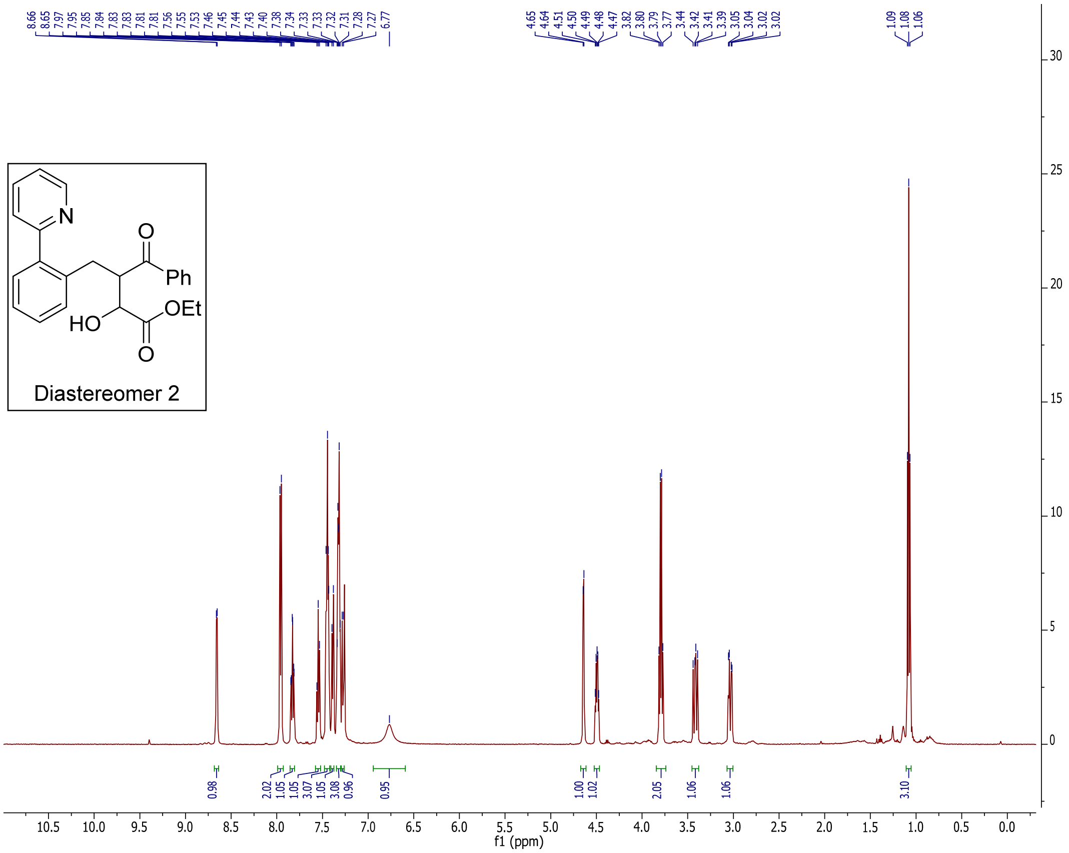

8.66 8.65 7.97 7.95 7.85 7.84 7.83 7.83 7.81 7.81 7.56 7.55 7.53 7.46 7.45 7.44 7.43 7.40 7.38 7.34 7.33 7.33 7.32 7.31 7.28 7.27 6.77
4.65 4.64 4.51 4.50 4.49 4.48 4.47 3.82 3.80 3.79 3.77 3.44 3.42 3.41 3.39 3.05 3.04 3.02 3.02
1.09 1.08 1.06
N
O
Ph
OEt
HO
O
Diastereomer 2
0.98 2.02 1.05 1.05 3.07 1.05 3.08 0.96 0.95 1.00 1.02 2.05 1.06 1.06 3.10
f1 (ppm)


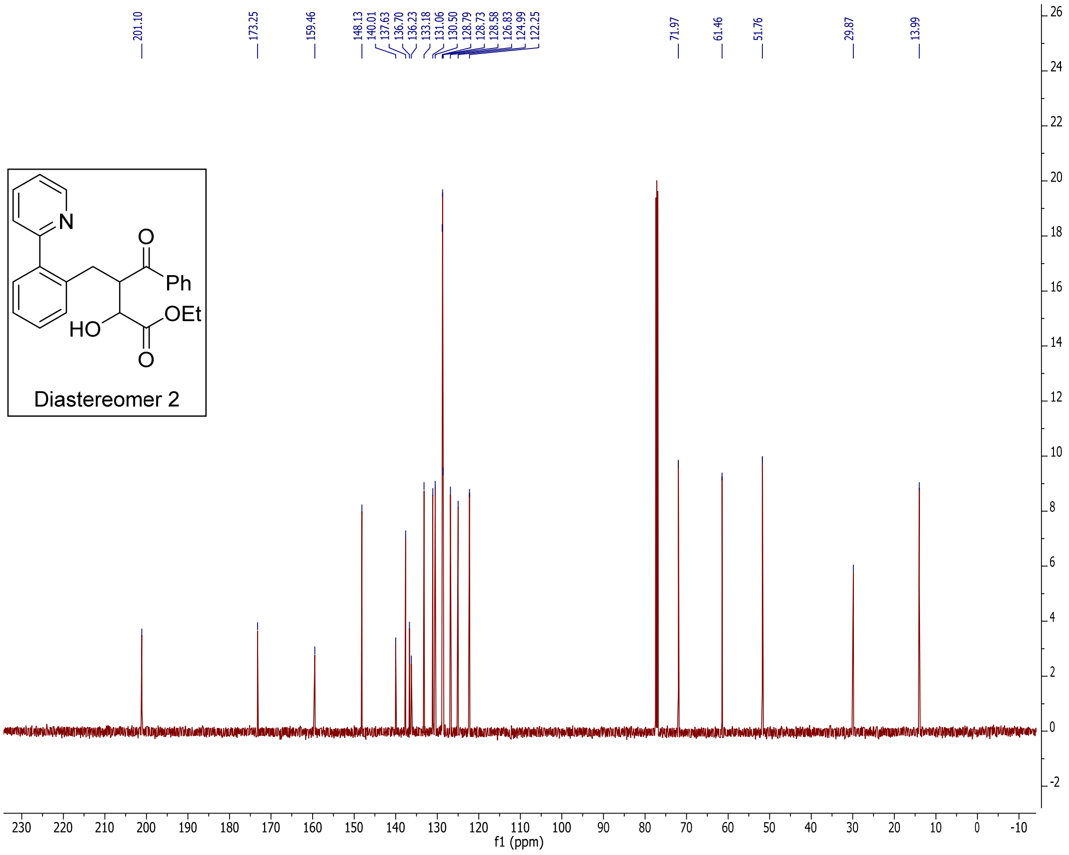

201.10
173.25
159.46
148.13 140.01 137.63 136.70 136.23 133.18 131.06 130.50 128.79 128.73 128.58 126.83 124.99 122.25
71.97
61.46
51.76
29.87
13.99
N
O
Ph
OEt
HO
O
Diastereomer 2
f1 (ppm)

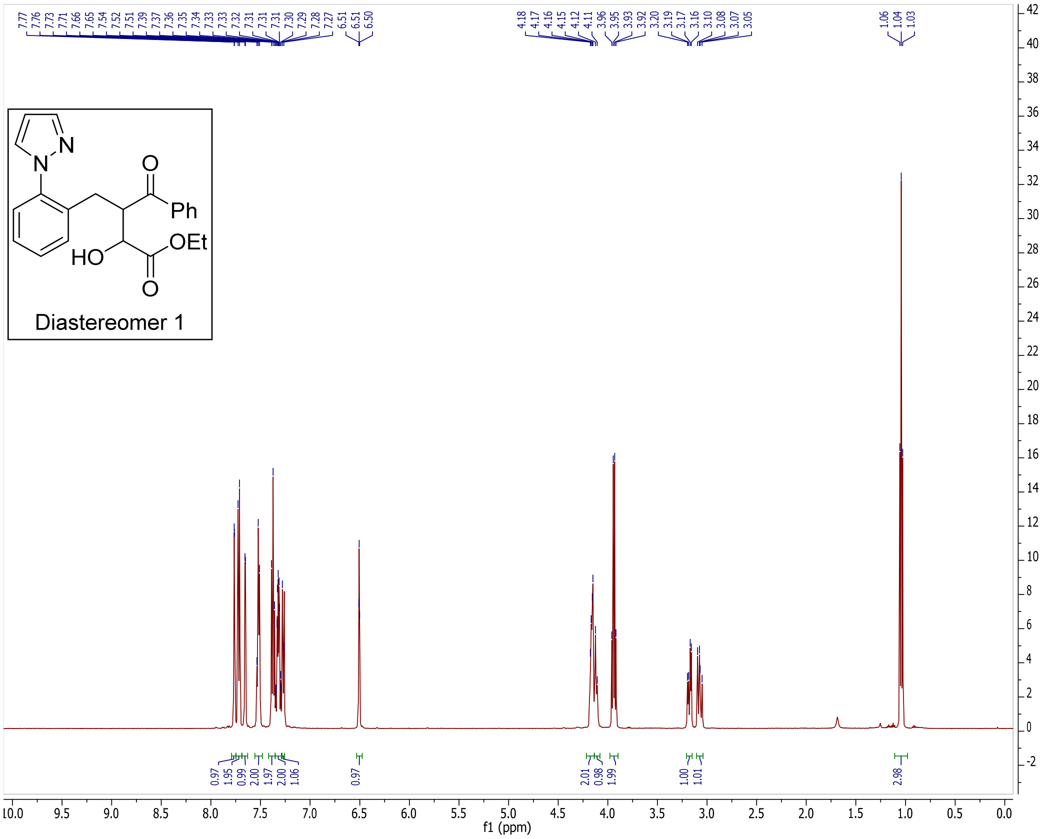
Diastereomer 1
f1 (ppm)

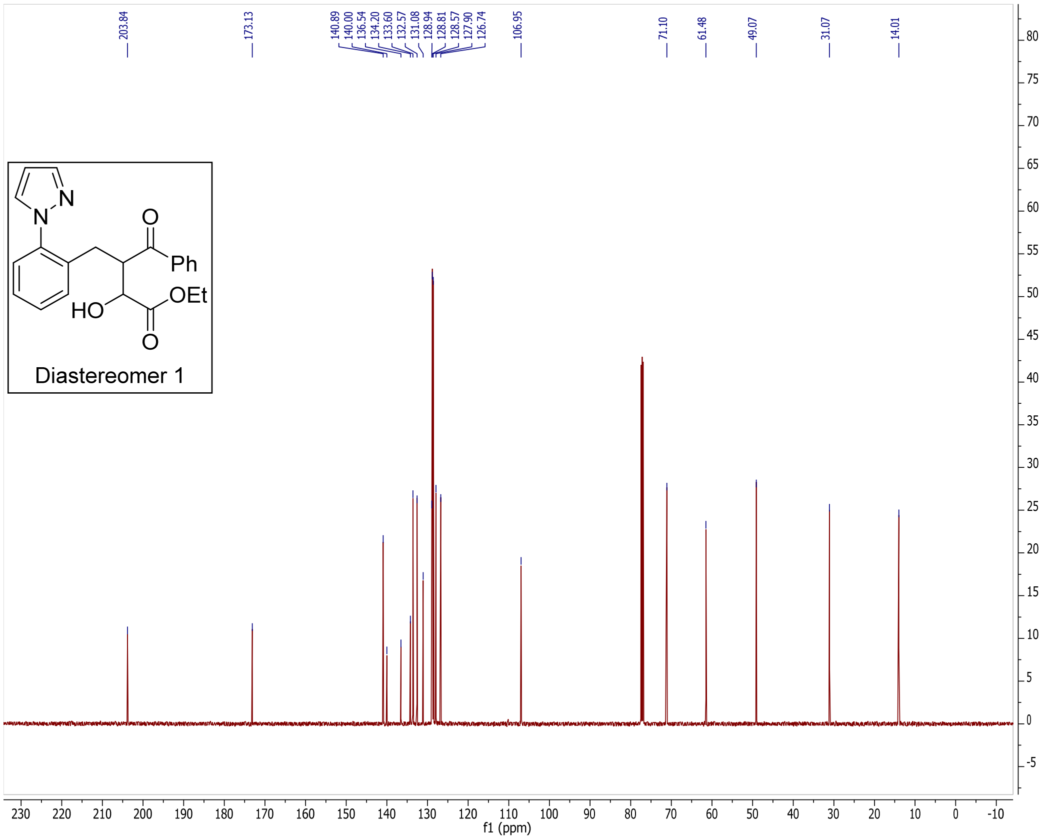
203.84
173.13
106.95
71.10
61.48
49.07
31.07
14.01
Diastereomer 1
f1 (ppm)

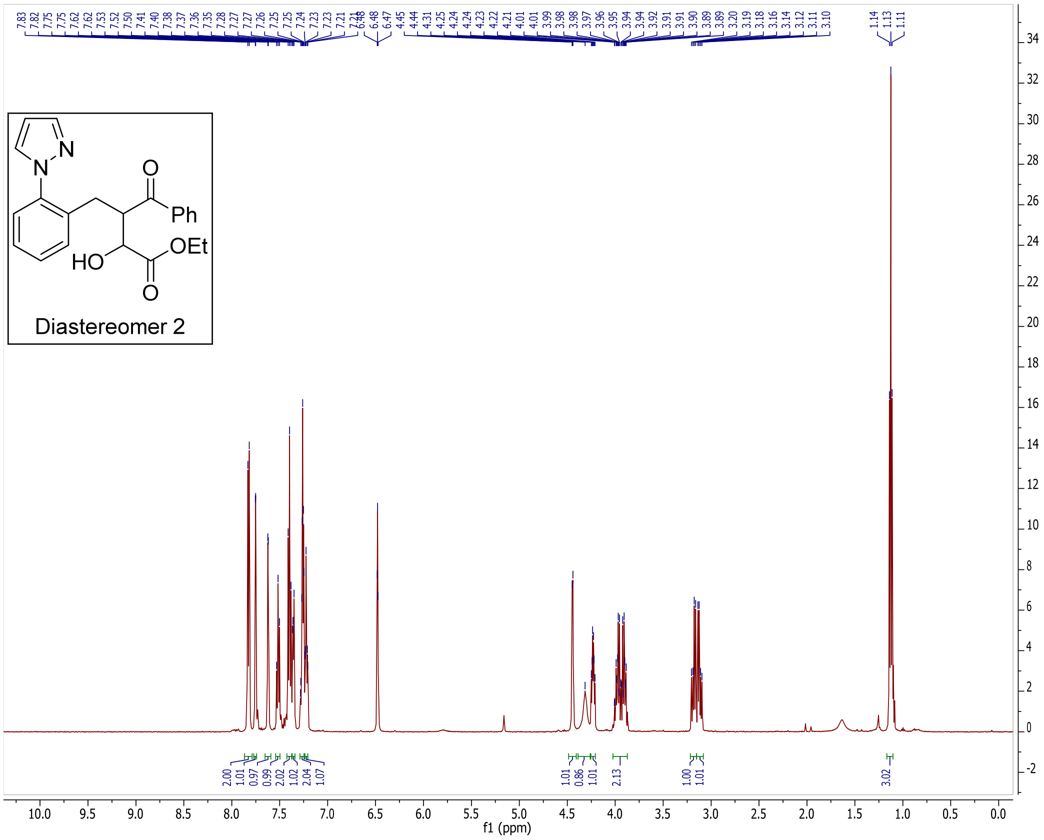

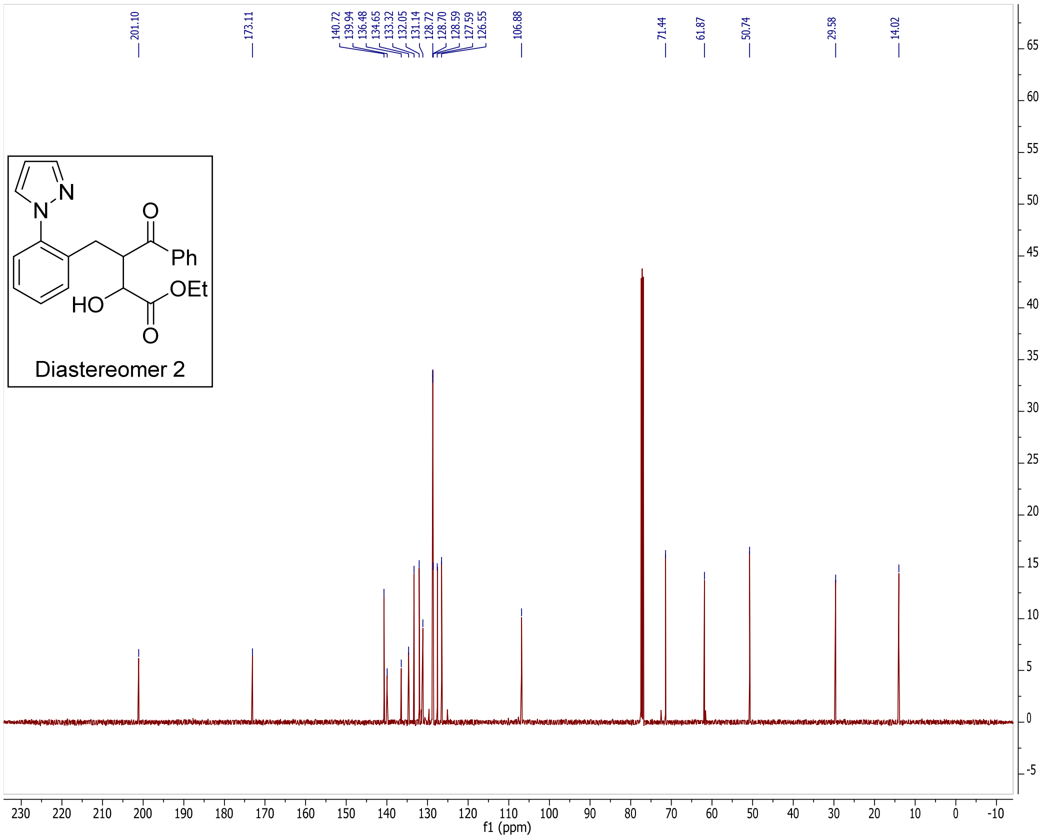

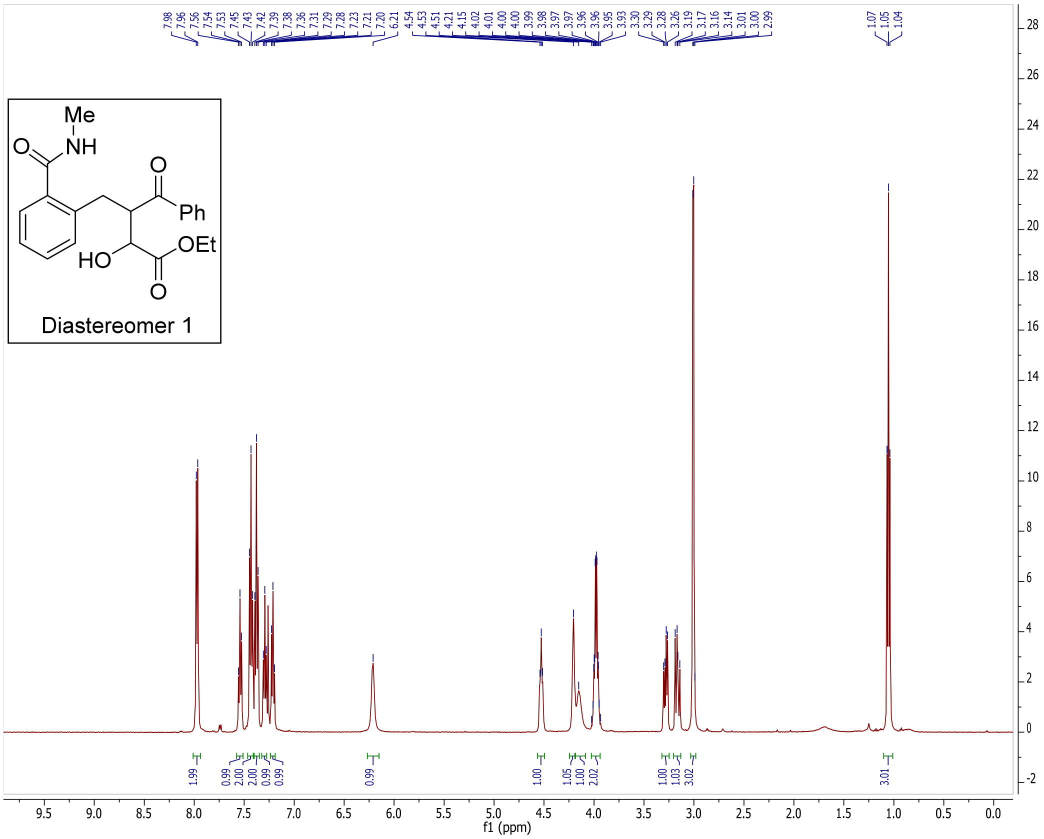
Me
NH
O
O
Ph
OEt
HO
O
Diastereomer 1
f1 (ppm)

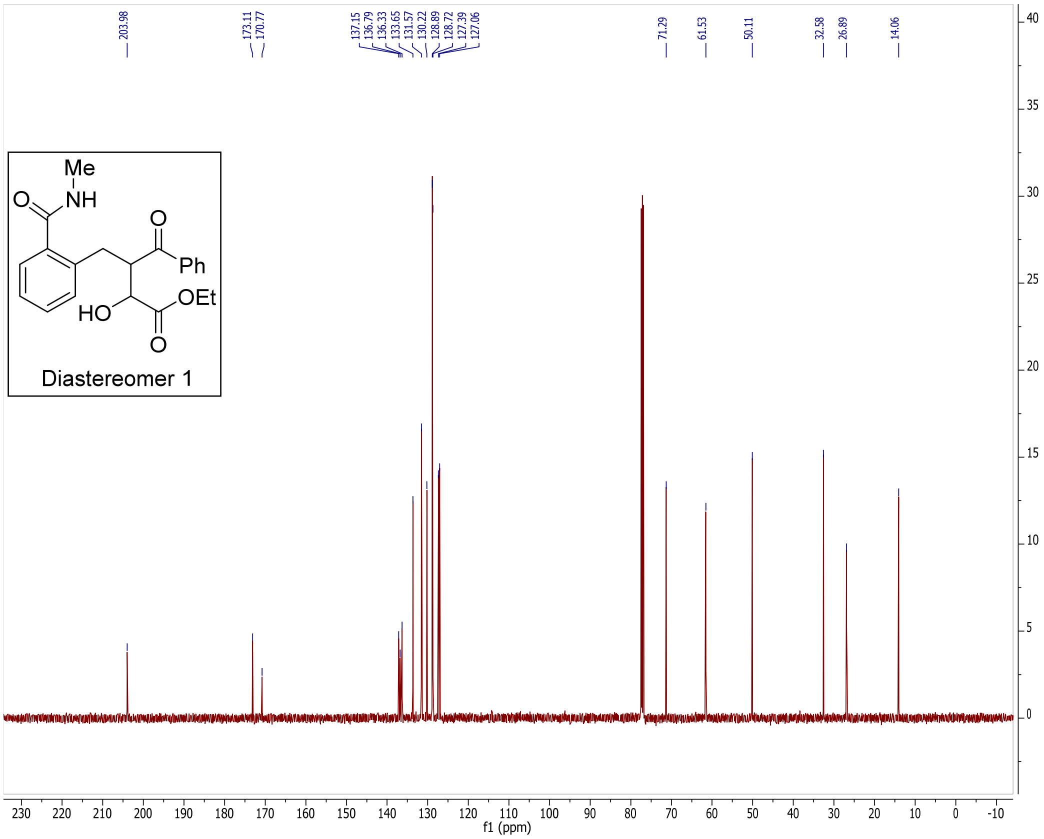
Me
NH
O
O
Ph
OEt
HO
O
Diastereomer 1
f1 (ppm)

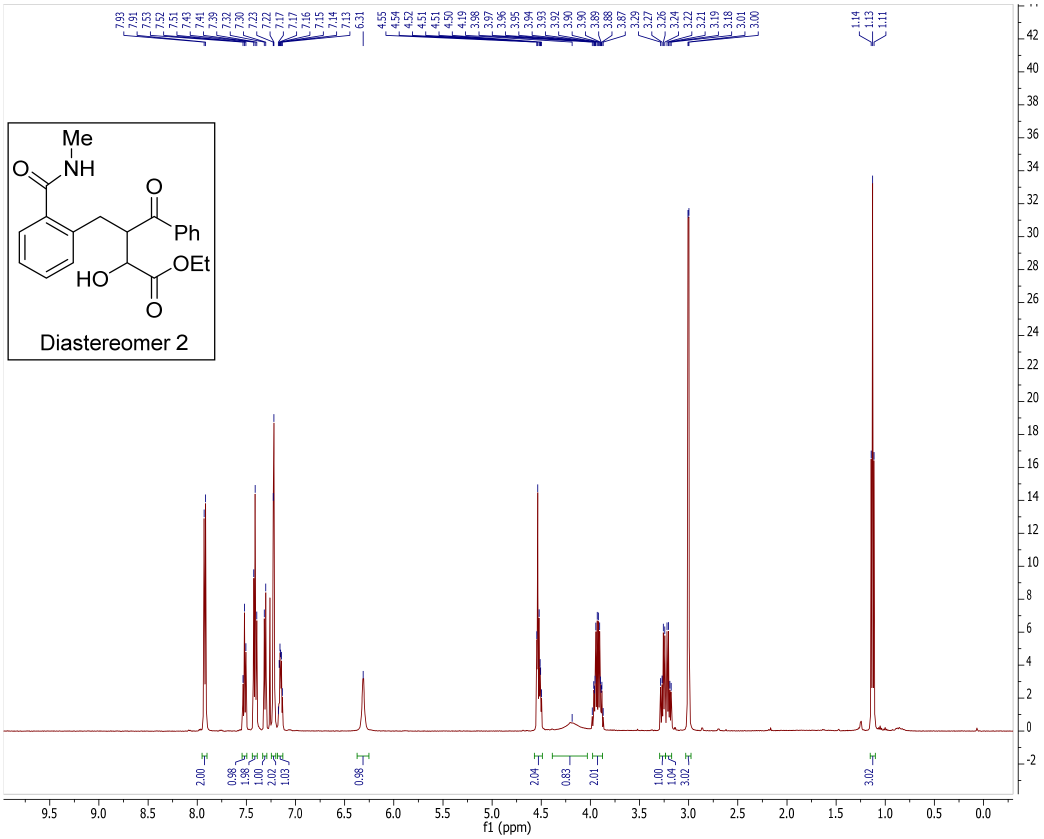
Me
NH
O
O
Ph
OEt
HO
O
Diastereomer 2
f1 (ppm)

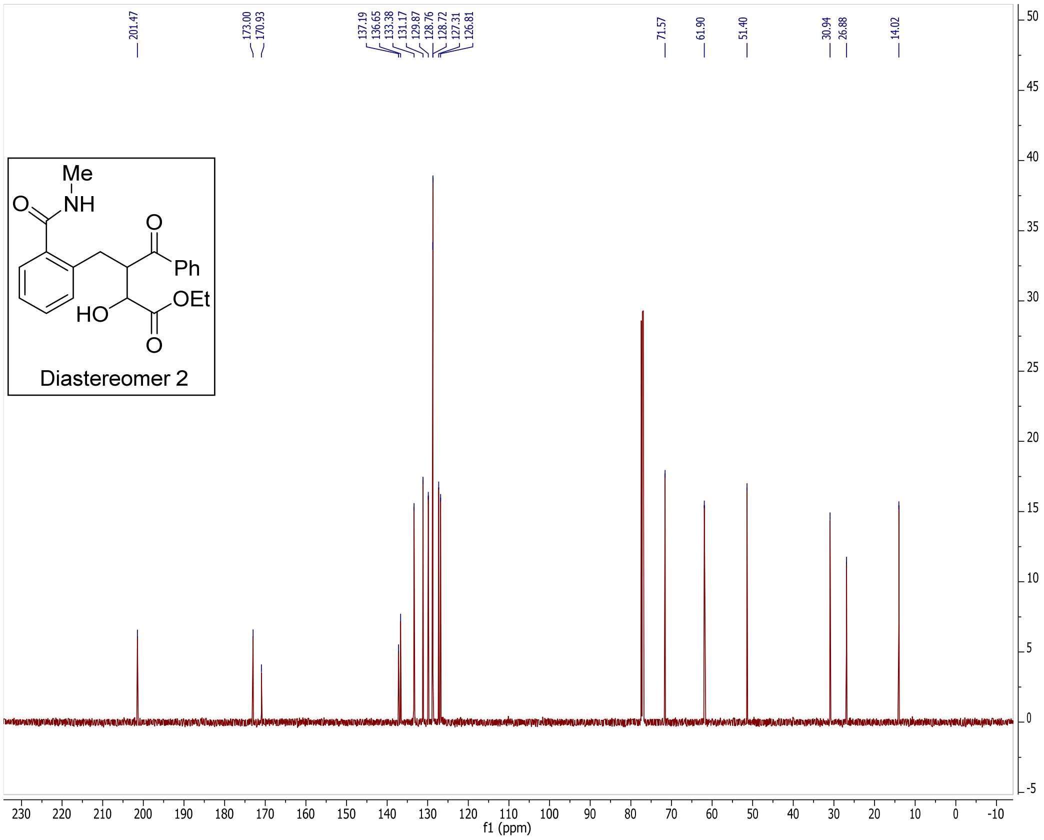
Me
NH
O
O
Ph
OEt
HO
O
Diastereomer 2
f1 (ppm)

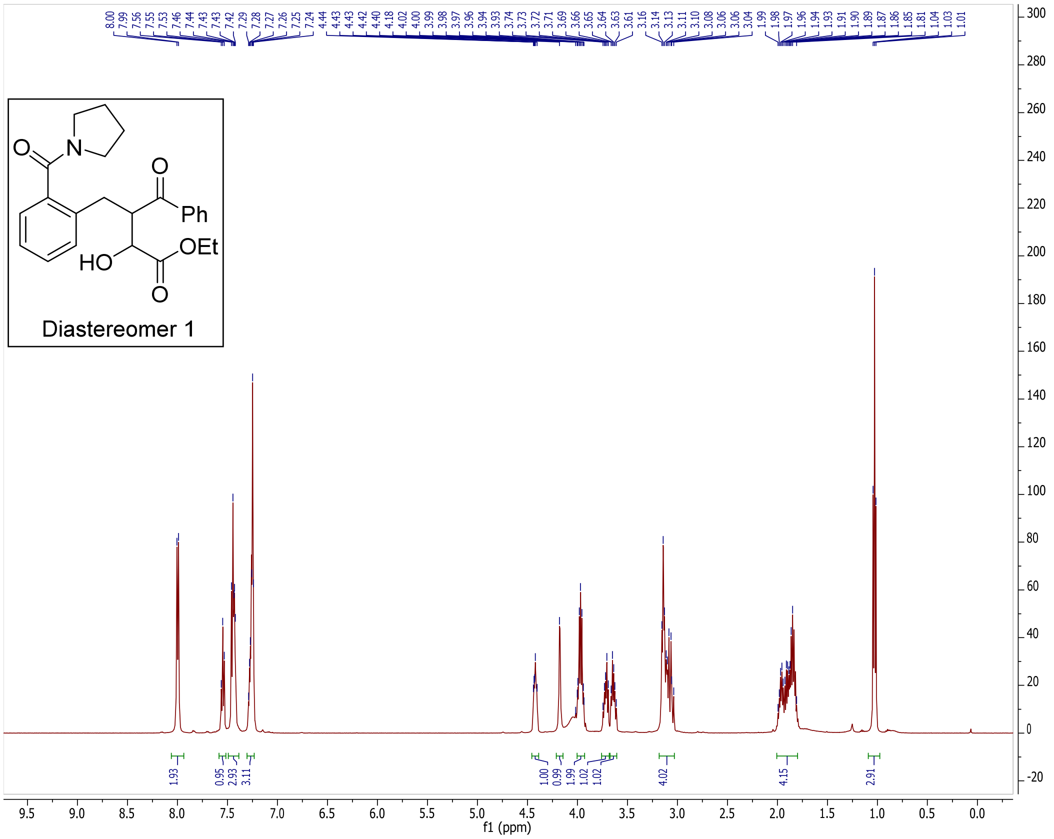

O
N
O
Ph
OEt
HO
O
Diastereomer 1
f1 (ppm)


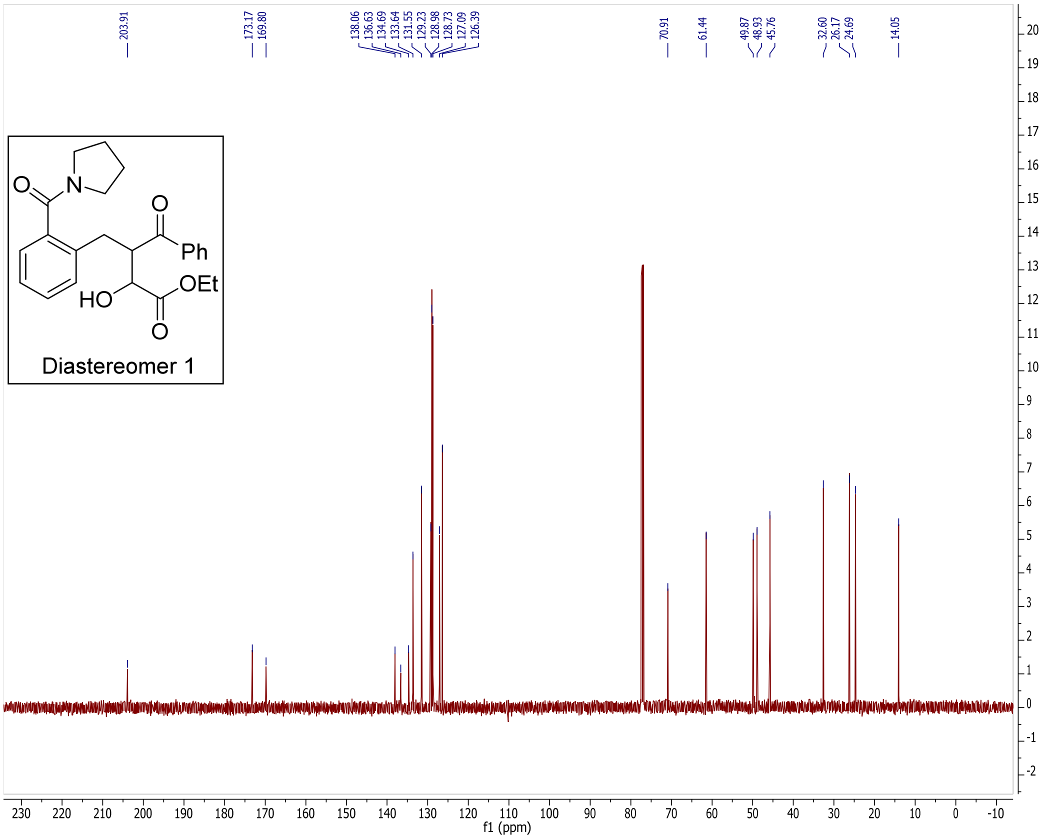

203.91
173.17
169.80
138.06
136.63
134.69
133.64
131.55
129.23
128.98
128.73
127.09
126.39
70.91
61.44
49.87
48.93
45.76
32.60
26.17
24.69
14.05
O
N
O
Ph
OEt
HO
O
Diastereomer 1
f1 (ppm)

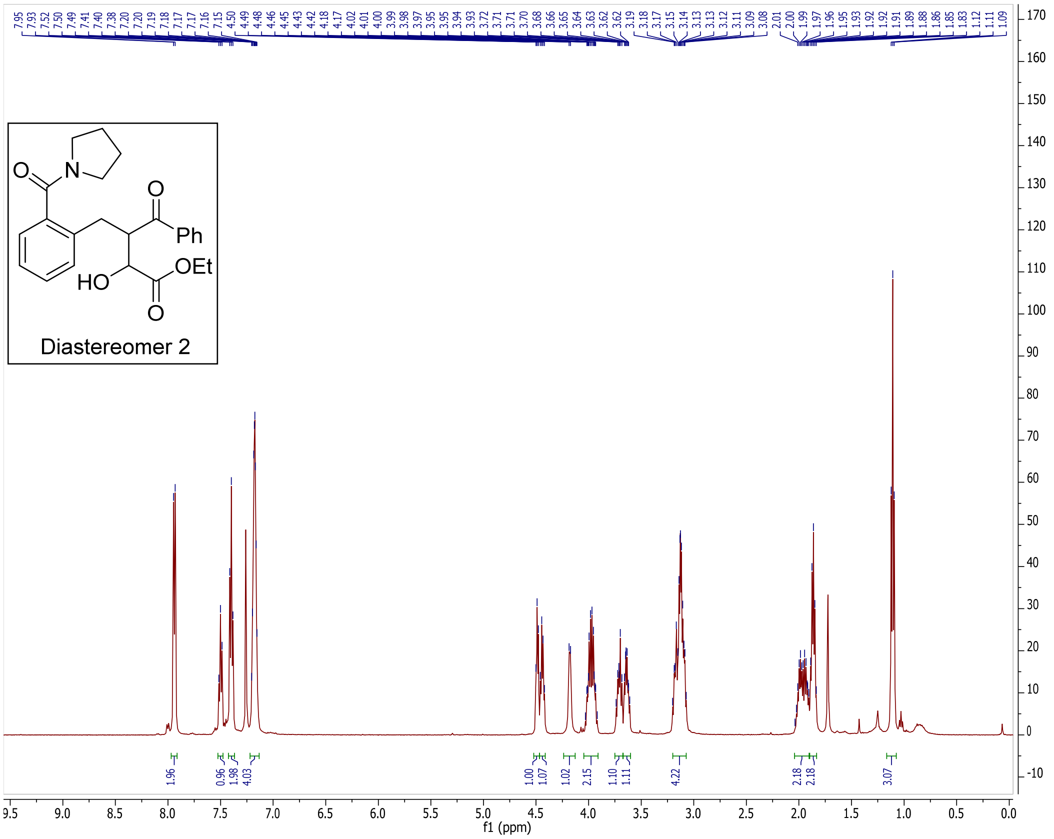
Diastereomer 2
f1 (ppm)

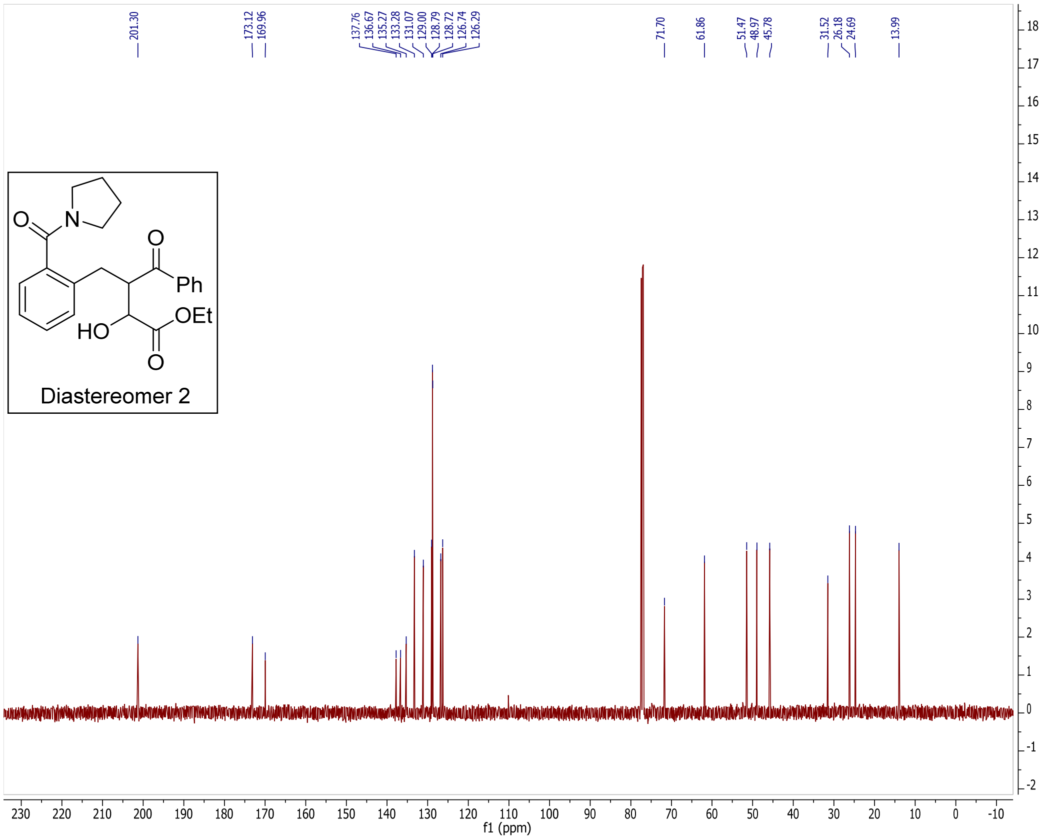
Diastereomer 2
f1 (ppm)

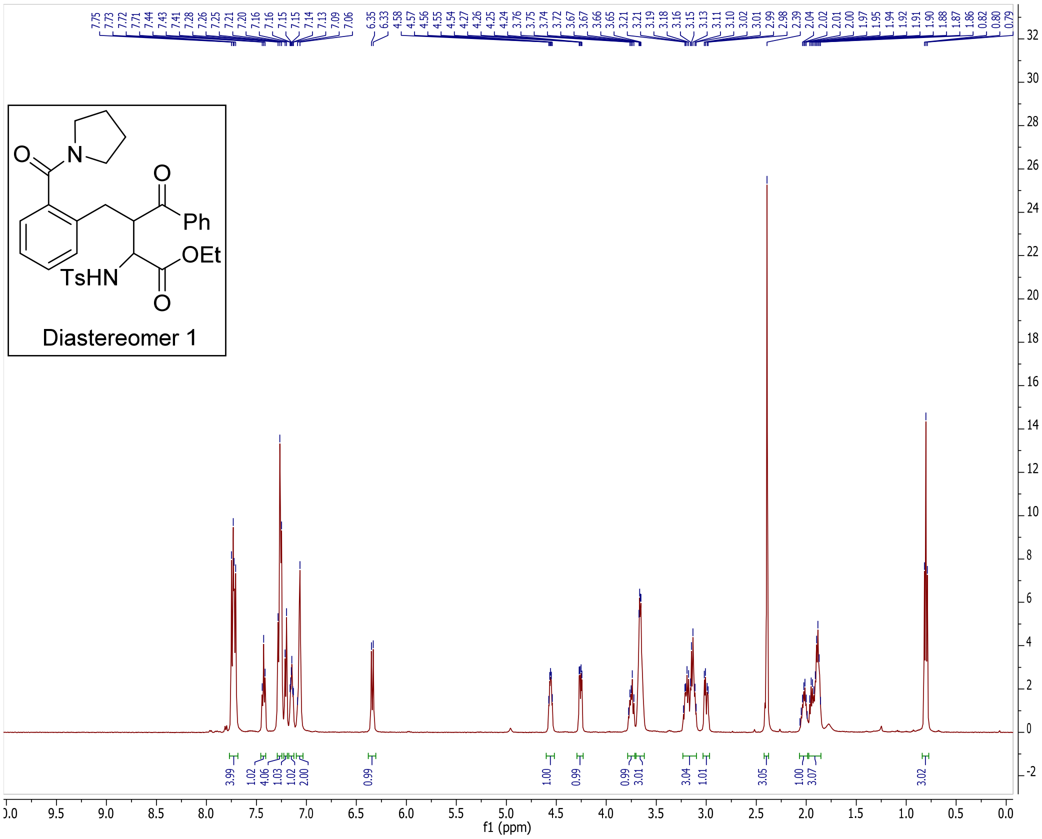

7.75 7.73 7.72 7.71 7.44 7.43 7.41 7.28 7.26 7.25 7.21 7.20 7.16 7.16 7.15 7.15 7.14 7.13 7.09 7.06 6.35 6.33 4.58 4.57 4.56 4.55 4.54 4.27 4.26 4.25 4.24 3.76 3.75 3.74 3.72 3.67 3.67 3.66 3.65 3.21 3.21 3.19 3.18 3.16 3.15 3.13 3.11 3.10 3.02 3.01 2.99 2.98 2.39 2.04 2.02 2.01 2.00 1.97 1.95 1.94 1.92 1.91 1.90 1.88 1.87 1.86 0.82 0.80 0.79
O
N
O
Ph
OEt
TsHN
O
Diastereomer 1
3.99 1.02 4.06 1.03 1.02 2.00 0.99 1.00 0.99 0.99 3.01 3.04 1.01 3.05 1.00 3.07 3.02
f1 (ppm)


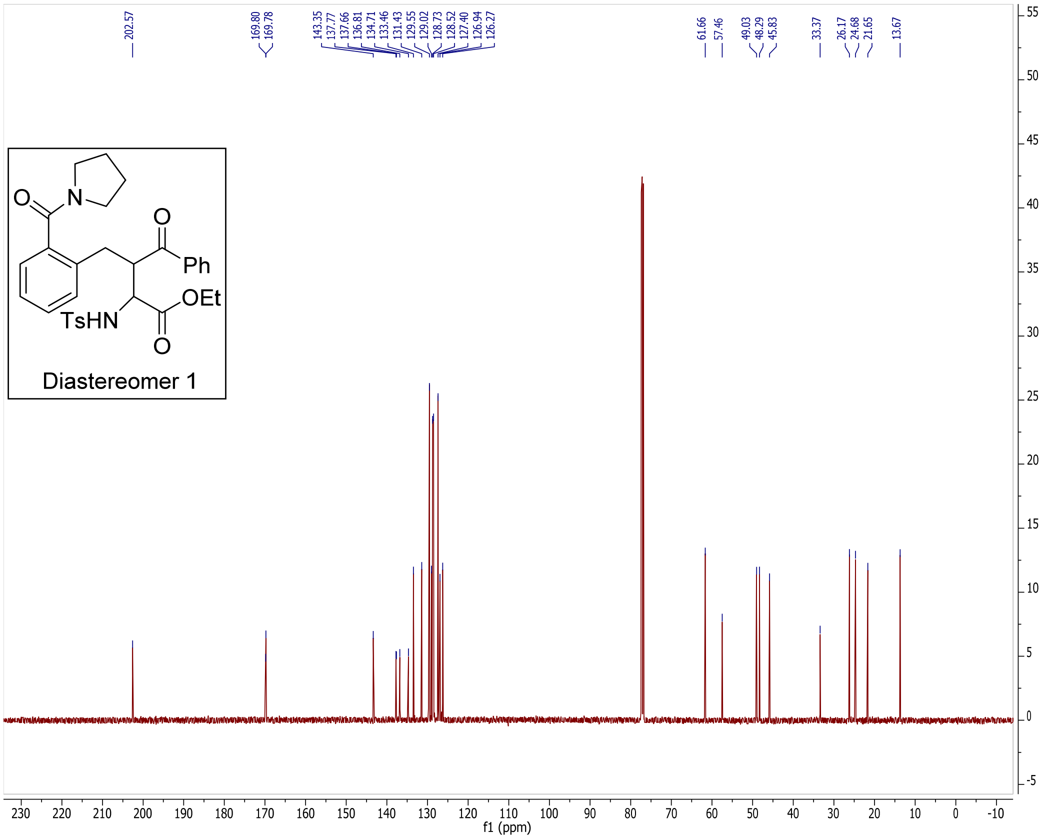

202.57 169.80 169.78 143.35 137.77 137.66 136.81 134.71 133.46 131.43 129.55 129.02 128.73 128.52 127.40 126.94 126.27 61.66 57.46 49.03 48.29 45.83 33.37 26.17 24.68 21.65 13.67
O
N
O
Ph
OEt
TsHN
O
Diastereomer 1
f1 (ppm)

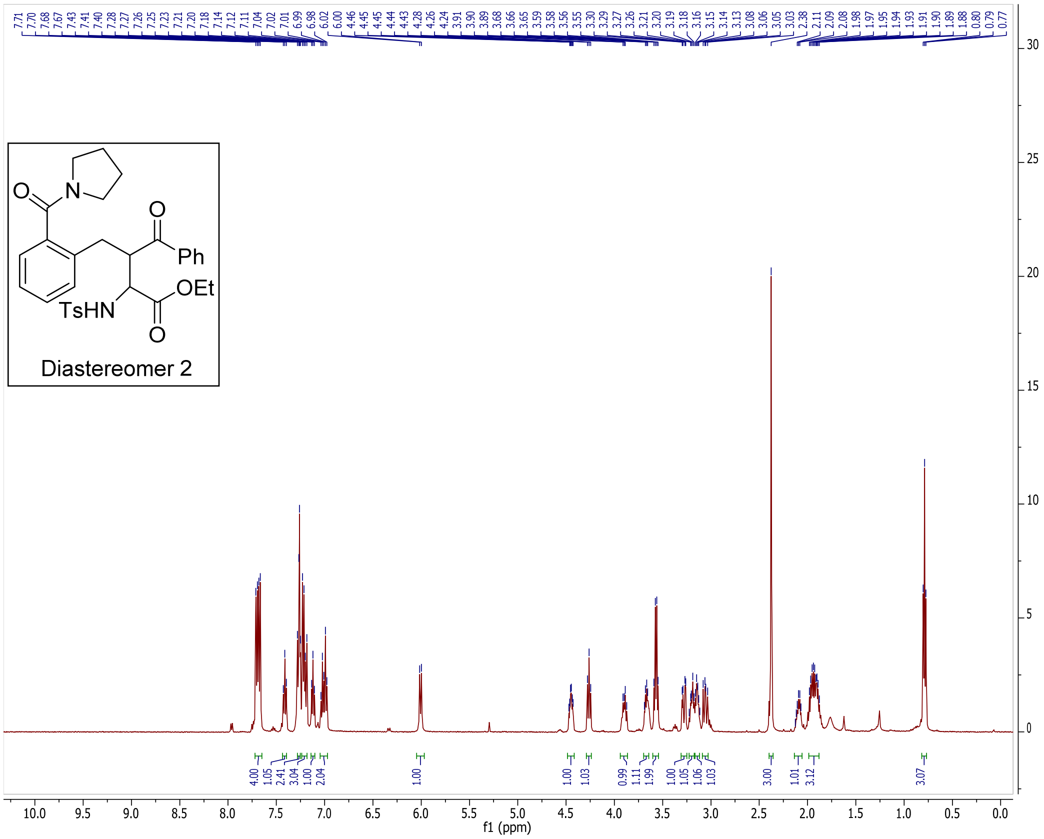
Diastereomer 2
O
N
O
Ph
OEt
TsHN
O
7.71 7.70 7.68 7.67 7.43 7.41 7.40 7.28 7.27 7.26 7.25 7.23 7.21 7.20 7.18 7.14 7.12 7.11 7.04 7.02 7.01 6.99 6.98 6.02 6.00 4.46 4.45 4.45 4.44 4.43 4.28 4.26 4.24 3.91 3.90 3.89 3.68 3.66 3.65 3.59 3.58 3.56 3.55 3.30 3.29 3.27 3.26 3.21 3.20 3.19 3.18 3.16 3.15 3.14 3.13 3.08 3.06 3.05 3.03 2.38 2.11 2.09 2.08 1.98 1.97 1.95 1.94 1.93 1.91 1.90 1.89 1.88 0.80 0.79 0.77
4.00 1.05 2.41 3.04 1.00 2.04 1.00 1.00 1.03 0.99 1.11 1.99 1.00 1.05 1.06 1.03 3.00 1.01 3.12 3.07
f1 (ppm)

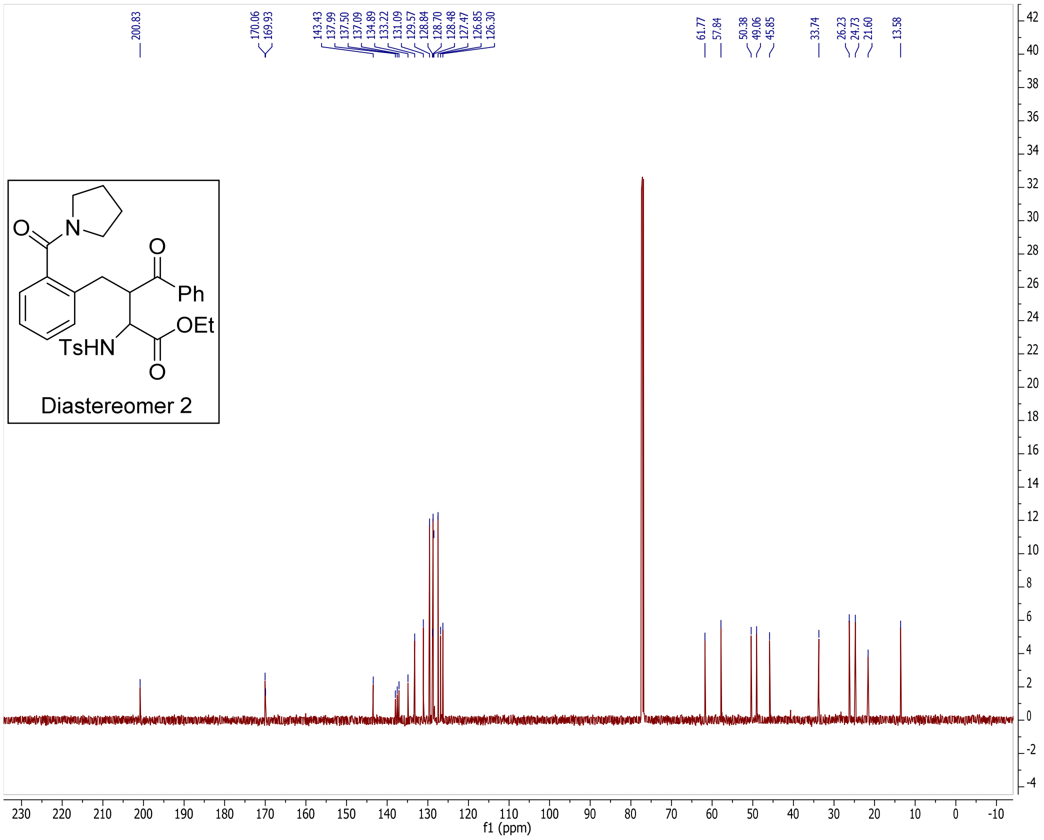
Diastereomer 2
O
N
O
Ph
OEt
TsHN
O
200.83
170.06 169.93
143.43 137.99 137.50 137.09 134.89 133.22 131.09 129.57 128.84 128.70 128.48 127.47 126.85 126.30
61.77 57.84 50.38 49.06 45.85 33.74 26.23 24.73 21.60 13.58
f1 (ppm)

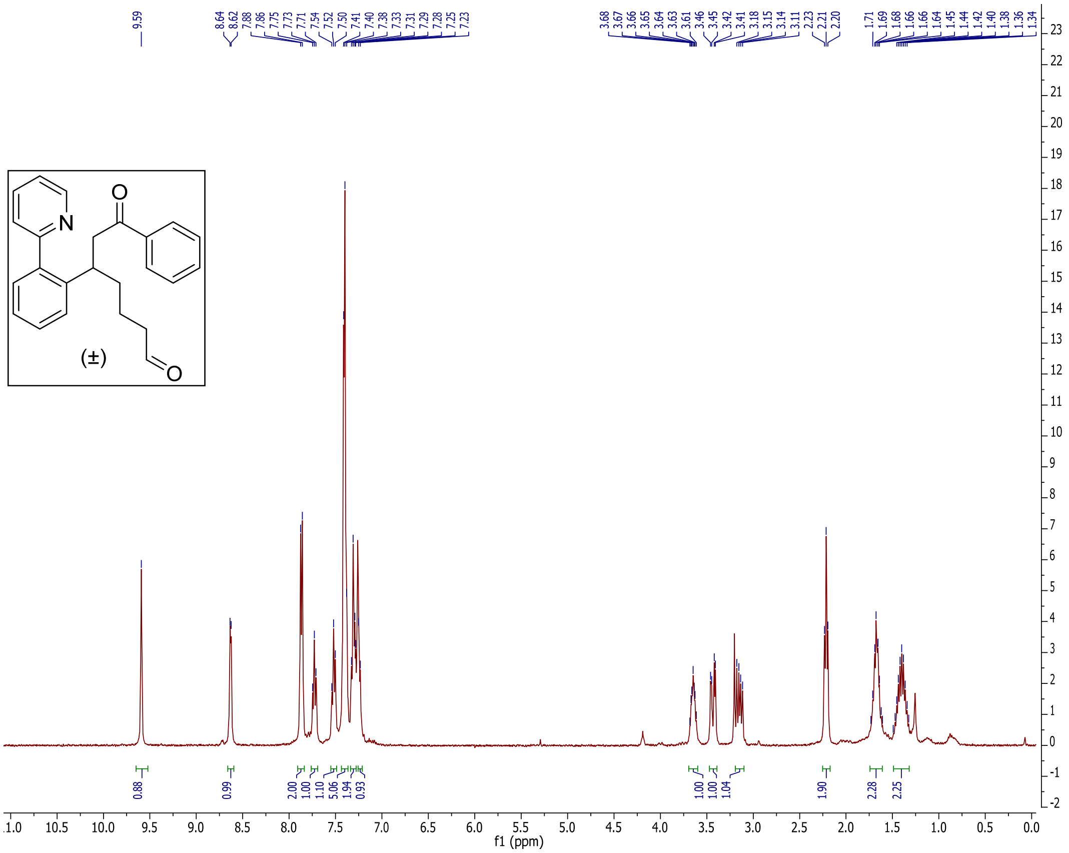

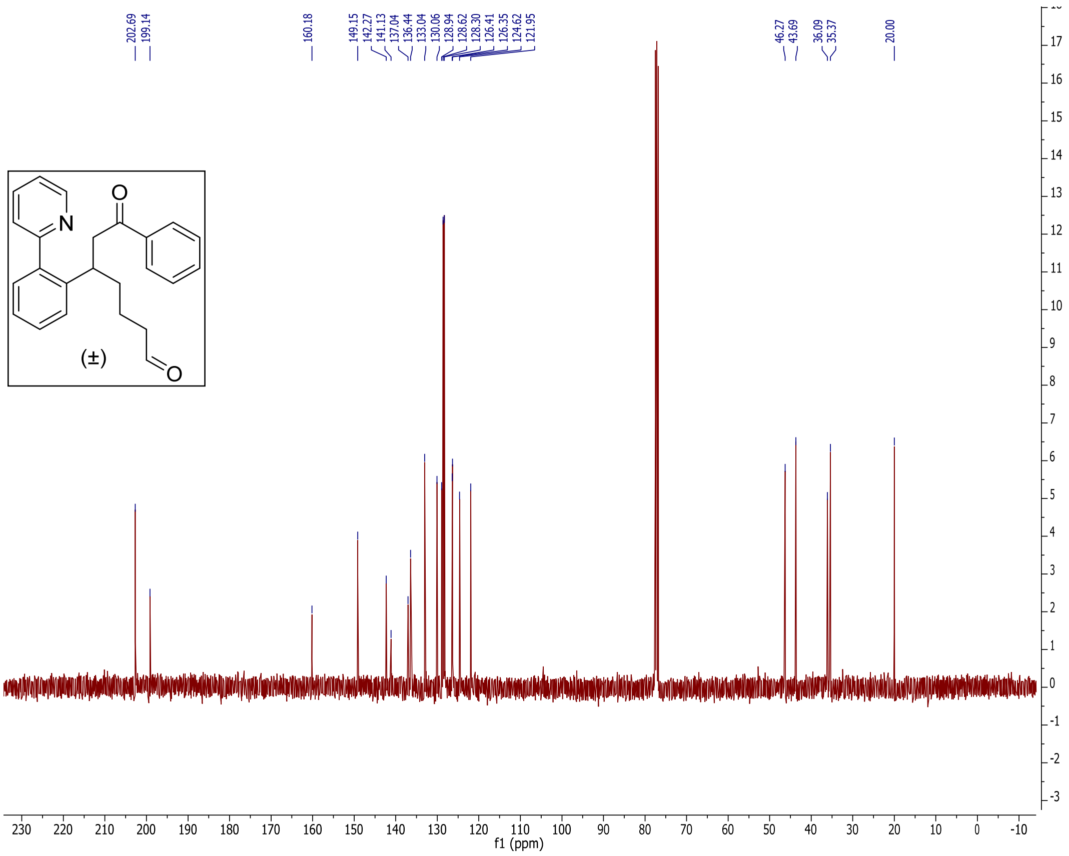

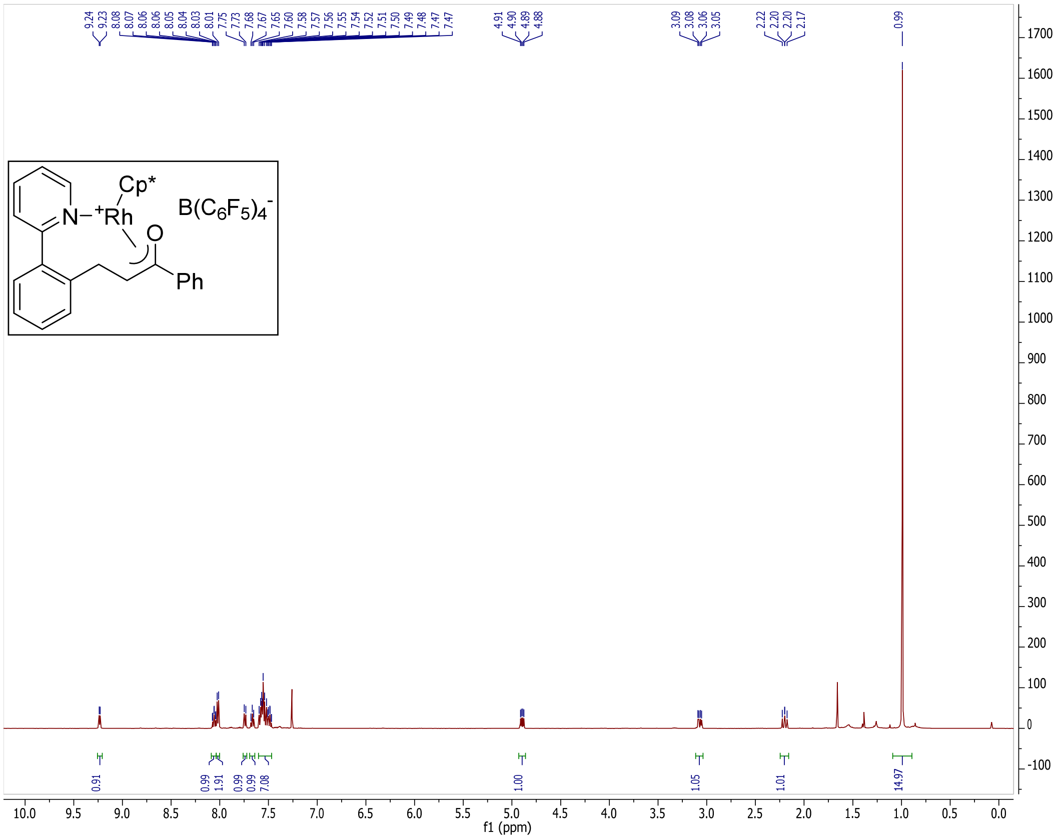

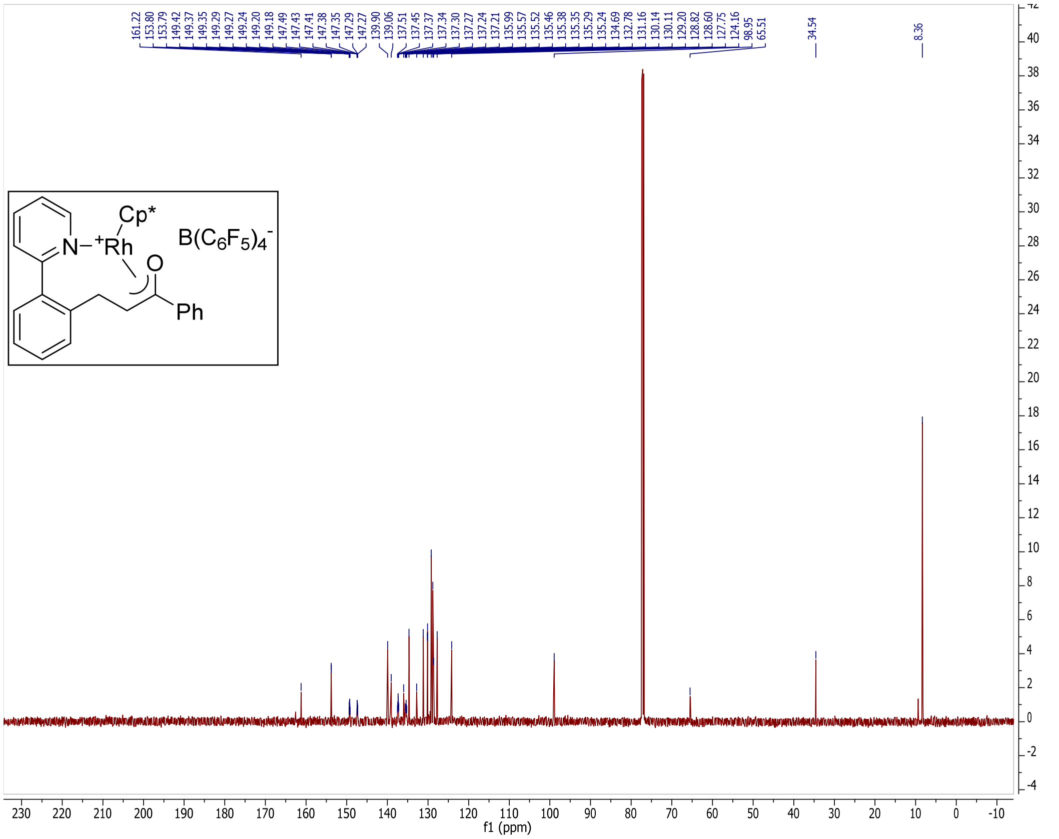

Supplement: Supplementary file 1 [file SC-007-C5SC04138D-s001.pdf]
